# Modeling actin-microtubule crosstalk in migrating cells

**DOI:** 10.1016/j.bpj.2025.09.029

**Published:** 2025-09-23

**Authors:** Pinaki Nayak, Anil Kumar Dasanna, Raja Paul, Heiko Rieger

**Affiliations:** 1 School of Mathematical and Computational Sciences, Indian Association for the Cultivation of Science, Kolkata, India; 2 Department of Physical Sciences, Indian Institute of Science Education and Research Mohali, Knowledge City, Manauli, India; 3 Center for Biophysics & Department for Theoretical Physics, Saarland University, Saarbrücken, Germany

## Abstract

Actin-microtubule crosstalk regulates the polarity and morphology of migrating cells and encompasses mechanical interactions, mediated by cross-linkers, molecular motors, and cytoskeletal regulators. Recent experiments indicate that local microtubule depolymerization promotes local actomyosin retraction, whereas local microtubule polymerization promotes local actin polymerization. Based on these observations, we develop a computational whole-cell model involving dynamic microtubules interacting mechanically and chemically with an active cell boundary. Specifically, the tips of microtubules send signals for local expansion or contraction to the active cell boundary, depending on whether they are in the growth or shrink phase. A rich, self-organized, dynamic behavior emerges, characterized by the repositioning of the microtubule-organizing center relative to the nucleus and the direction of migration. This also includes a variety of migration patterns, cell morphologies, and complex responses to obstacles in microfluidic and obstacle park environments. We demonstrate that microtubule length and numbers have a significant impact on these features, highlighting the need for new experimental investigations. Thus, the model provides a unified framework that explains a wide range of experimental observations and setups where actin-microtubule crosstalk plays a crucial role.

## Significance

The interplay between microtubule dynamics, centrosome positioning, cell polarization, and migration is fascinating and enigmatic. Recent experiments have demonstrated a strong correlation between microtubule growth and shrinkage and actin protrusion and actomyosin contraction, mediated by actin-microtubule crosstalk. In this study, we introduce, for the first time to our knowledge, a quantitative model of cell migration that accounts for actin-microtubule crosstalk and uncovers a rich, self-organized dynamic behavior, including cell polarization, centrosome positioning, migration patterns, cell morphologies, and intricate responses in microfluidic environments and synthetic obstacle arrays. Thus, our work represents a significant advancement in the modeling and understanding of cell migration in complex environments and provides a powerful method to incorporate actomyosin dynamics into computational models.

## Introduction

Cell migration is primarily driven by forces generated at the actin cortex underlying the cell membrane. The onset of migration requires the cell to be polarized by forming a protruding front edge and a contracting rear edge (1). Protrusions at the front originate from the increased actin polymerization supported by the focal adhesions formed in contact with the extracellular matrix (2,3,4). Membrane retraction at the rear is realized inside a cell by contractile forces arising from myosin activity and dissolution of focal adhesions (2,5,6). The formation of protrusion or membrane retraction is guided through the reorganization of the cytoskeleton by the delivery of molecular regulatory signals. Microtubules (MTs) are known to play an important role in the distribution of these regulatory signals leading to cell polarization during migration (7,8,9). The tips of growing MTs reach the protruding front edge of the cell to deliver actin polymerization signals that stabilize the protrusions (6,10,11). MT depolymerization induces the activation of RhoA, which increases myosin-II activity, increasing contractility and cell membrane retraction (12,13,14). Differential stability of MTs at the front and rear edge thus leads to symmetry breaking and polarization of the cell (6,15). MTs have also been suggested to play a critical role in the modulation of cell shape and stabilization or retraction of protrusions when the cell is navigating through obstacles, thereby dictating the cell migration path (6).

Centrosomes, being the primary MT organizing centers (MTOCs) in animal cells, can guide the choice of the front and rear edge of the cell, via their preferential position with respect to the nucleus. The centrosomal position, anterior or posterior to the nucleus, has been investigated in various cells, proposing mechanisms that may guide this choice. Cells undergoing mesenchymal migration are characterized by the formation of nascent focal adhesions with the extracellular matrix at the leading edge and the rupture of aged focal adhesions at the rear edge (16,17,18). The centrosome is placed ahead of the nucleus more often in cells migrating through stiff extracellular matrices (19). Fast-moving ameboid cells, characterized by reduced focal adhesions, are typically known to position their centrosome posterior to the nucleus during migration (20). Interestingly, recent experimental studies suggest that leukocytes can alternate between a centrosome-forward or nucleus-forward configuration while navigating a congested microenvironment (21). Cells may alter the position of the centrosome to modify the distribution of MTs, which in turn helps coordinate the polymerization and contraction signals in the actin cortex that drive cell movement. As MTs grow, they can extend toward the cell membrane or nucleus and undergo buckling. This buckling generates a pushing force that pushes the centrosome away from the point of contact with either the cell or nuclear membrane (22,23). MTs can also slide along the cell membrane or nucleus (24). Dynein motors present at the cortex or nuclear membrane attach to the sliding MTs and walk toward their minus end, effectively pulling the MTs and the centrosome (25,26). The position of the centrosome within a moving cell results from a complex balance of forces, which are generated through the interactions of the MTs with the centrosome, along with membrane remodeling driven by polarity signals from the MTs to the cortex. This raises several questions: how does the centrosome position itself anterior or posterior to the nucleus during migration? How do changes in MT dynamics influence the centrosome’s position? And, what impact do these alterations in the MT network have on actin-MT interactions and the cell’s overall migration?

Phenomenological and mechanistic acto-myosin models of cell migration have been the subject of extensive study (27,28,29,30,31,32). Models that focus on the microscopic details of actin polymerization and myosin motor activity are computationally intensive and challenging to generalize for studying cell migration in both two (2D) and three dimensions (3D) (33,34). In contrast, phenomenological models of cell migration often focus on cortical activity and membrane-substrate interactions but tend to neglect the contribution of MT-driven chemical signaling, which plays a key role in regulating cortical dynamics and initiating symmetry breaking (29,35,36,37,38).

Although it is well established that MT-actin crosstalk plays a crucial role in cell migration and other cellular functions (39), a mechanistic whole-cell model integrating MT dynamics and actin-generated forces to explore the self-organization of centrosome positioning, cellular shape changes, and migratory behavior is still lacking. In this work, we therefore introduce a phenomenological model based on the experimental observations reported in Refs. (6,7,10,13,15), which correlate MT growth and shrinkage with actin polymerization, depolymerization, and contraction. We examine how the position of the centrosome and the direction of migration are influenced by MT dynamics. Additionally, we explore how cells might leverage MT-actin crosstalk to switch between persistent migration and diffusive movement. Finally, we investigate how the positioning of the centrosome, either anterior or posterior to the nucleus, may guide the cell’s path when navigating narrow channels.

## Material and methods

### Computational model

We develop a mechanistic model of cell migration based on membrane protrusion and retraction coupled to the actomyosin cortex and cytoskeleton. Key processes include actin polymerization, myosin-driven contraction, actin polarization, focal adhesion dynamics, and membrane tension. MT depolymerization regulates actomyosin contraction via Rho GTPase signaling (6), whereas MT polymerization promotes actin-driven protrusions by transporting signaling molecules to the leading edge (6,10,39). These two effects form the basis of our whole-cell MT-actin crosstalk model during migration.

**Figure 1:**
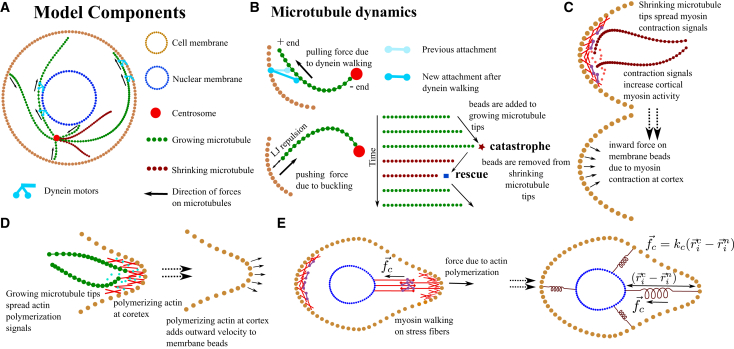
Schematic of the model. (*A*) Sketch of a migrating cell, showing membrane, nucleus, centrosome, MTs, actin, and dynein motors. (*B*) MTs grow by adding beads at the plus end and shrink by bead removal. Growing MTs can undergo catastrophe and shrink, whereas shrinking MTs can be rescued. Dynein, anchored to the cell and nuclear membranes, attaches to MTs and moves toward the minus end, exerting pulling forces on the centrosome. Steric interactions modeled as Lennard-Jones repulsive forces between MT beads and the membrane cause MT buckling and generate pushing forces. (*C*) Shrinking MTs transmit myosin contraction signals to the actin cortex, producing inward-directed forces on membrane beads. (*D*) Growing MT tips deliver actin polymerization signals, driving outward membrane bead displacement. (*E*) Myosin contractility across overlapping actin filaments couples the nuclear and cell membranes through a linear elastic force that resists membrane protrusion.

We model 2D mesenchymal migration using bead-spring loops to represent the semiflexible boundaries of the cell and nucleus, and bead-spring polymers for dynamic MTs that grow or shrink at their plus ends. MTs are anchored at the MTOC, assumed to coincide with the centrosome. Growing MTs apply pushing forces when contacting the membrane, whereas dyneins anchored at the cell and nuclear membranes exert pulling forces on MTs (40,41). A schematic of these model components is shown in Fig. 1*A* and *B*, with full mathematical details in the supporting material.

MT-actin crosstalk is incorporated through regulatory signals transmitted by MT tips. Shrinking MTs trigger local myosin activation, causing membrane contraction, whereas growing MTs promote actin polymerization, driving membrane protrusion. Rather than modeling the actin network explicitly, we represent their effects through effective forces on membrane beads: shrinking MT tips apply inward forces proportional to their local density (6), and growing tips induce outward velocities proportional to the number of tips near a membrane segment (29,37,42). In addition, contractile forces generated by myosin between the nucleus and protrusion edge create an elastic coupling (38,43,44,45), which we model by connecting nucleus and membrane beads with springs (see Fig. 1 *C*–*E* and details in the supporting material).

## Results and Discussion

### Migrating cell centrosome can lead or trail nucleus depending upon MT length

**Figure 2:**
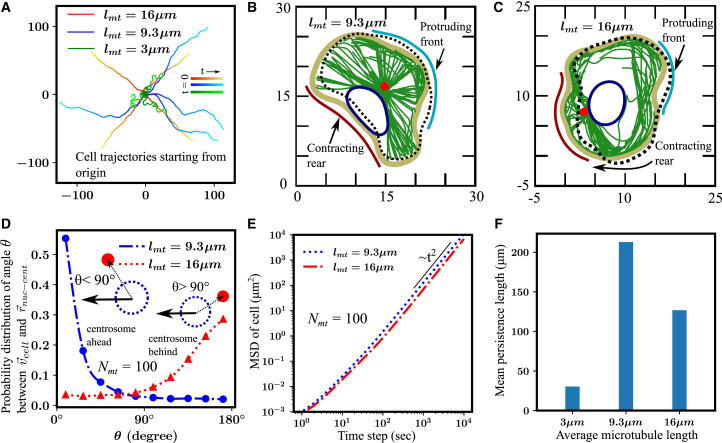
Centrosome position depends on MT length. (*A*) Cell trajectories show directed migration for $l_{mt}=9.3\;\mu m$ and 16μm but random motion for $l_{mt}=3\;\mu m$. (*B*) Simulations with $l_{mt}=9.3\;\mu m$ show the centrosome anterior to the nucleus; growing MT tips drive front protrusions, and cell rear is pulled via nucleus to membrane elastic coupling. (*C*) For $l_{mt}=16\;\mu m$, the centrosome shifts posterior; growing MTs promote front protrusions, whereas shrinking MTs trigger rear contraction. (*D*) Probability distribution of the angle between migration direction and centrosome-nucleus axis; $\theta <90^{{}^{\circ}}$ indicates centrosome ahead and $\theta >90^{{}^{\circ}}$ behind. Regular MTs (9.3μm) position the centrosome ahead and long MTs (16μm) behind. (*E*) Mean-square displacement shows $t^{2}$ scaling, indicating ballistic migration for $l_{mt}=9.3$–16μm. (*F*) Mean persistence length of migrating cells across different MT lengths, showing high persistence for $l_{mt}=9.3$–16μm (one-way ANOVA test *p*-value <0.0001).

First, we examine how centrosome positioning and migration characteristics in our model are influenced by MT properties, such as the average MT length. The trajectories of the cell centroid indicate that for average MT lengths of $l_{mt}=9.3-16\;\mu m$, the cell exhibited directed migration, whereas for an average MT length of $l_{mt}=3\;\mu m$ the cellular trajectories showed random motion (Fig. 2 *A*; see Fig. S1 and Videos S1 and S2). We then focused on the MT lengths that resulted in the directed migration of the cell (see Fig. 2 *B* and *C*). Our results showed that for an average MT length of $l_{mt}=9.3\;\mu m$ (which is two-thirds of the cell diameter, henceforth referred to as “regular MT”), the centrosome was predominantly positioned ahead of the nucleus (Fig. 2 *B* and *D* (blue line)). When the average MT length was increased to $l_{mt}=16\;\mu m$ (greater than the cell diameter, henceforth referred to as “long MT”), the centrosome preferentially remained behind the nucleus in the direction of migration (Fig. 2 *C* and *D* (red line)). To understand this preferential positioning of the centrosome in both cases, we examined the MT dynamics. MTs radiated from the centrosome in all directions, extending to both the cell and nuclear membranes. However, the presence of the nucleus obstructed the MTs from reaching the portion of the cell membrane behind it. Regular MTs were not long enough to slide along the nuclear membrane and extend to the rear portion of the cell membrane (Fig. 2 *B*). The growing MTs that reach the cell membrane have most of their length within the cytoplasm, with only a short segment (∼ 1.8μm per MT) near the MT tips remaining in close contact (distance less than $2\times 1.12\sigma_{\text{mt}}$) with the cell membrane (Fig. 2 *B*). The MT tips deliver actin polymerization signals to the membrane region near their tips. When an MT undergoes a catastrophe, its tip recedes from the membrane. For $l_{mt}=9.3\;\mu m$, only a few shrinking MT tips are observed near the membrane-cortex region (see Fig. S2 *A* and *B*). Hence, the contraction signals generated by shrinking MT tips play little role in cell polarization. In contrast, a large number of growing MTs are located at the membrane-cortex region closer to the centrosome (see Fig. S2 *A* and *B*). As a result, actin polymerization signals are mainly distributed in these areas with a high density of growing MT tips. Therefore, in this case, the difference in actin polymerization signals between membrane-cortex regions proximal and distal to the centrosome gives rise to cell polarization. As the cell advances, propelled by actin polymerization at the leading edge, the nucleus and rear regions are pulled forward through elastic coupling between the membrane and the nucleus (see Fig. S2 *A* and *B*). This way, asymmetry in the distribution of actin polymerization signals breaks the symmetry of the stationary cell. The regions receiving signals from growing MTs form the protruding front end, whereas areas devoid of MT tips form the rear. As a result, the cell migrates with the centrosome near the cell center and the nucleus positioned behind it toward the rear end of the cell. For regular MTs, we find that the probability distribution of the angle between the cell’s direction of motion and the vector from the nucleus to the centrosome peaks near zero (Fig. 2 *D* (blue line)), corroborating the result that the centrosome remains positioned ahead of the nucleus throughout migration.

Video S1. Persistent cell migration for *N*_*mt*_ = 100 and *l*_*mt*_ = 9.3 *μm*; persistent cell migration for *N*_*mt*_ = 50 and *l*_*mt*_ = 16 *μm*

Video S2. Diffusive cell migration for *N*_*mt*_ = 100 and *l*_*mt*_ = 3 *μm*; diffusive cell migration for *N*_*mt*_ = 200 and *l*_*mt*_ = 16 *μm*

Cells with long MTs ($l_{mt}=16\;\mu m$) are polarized with the centrosome positioned posterior to the nucleus. MT tips extending from the centrosome reach either the cell membrane or nuclear membrane and can glide along these membranes to eventually reach the distal end of the cell, behind the nucleus (Fig. 2 *C*). Since the average MT length exceeds the cell diameter, growing MTs tend to glide along the cell or nuclear membrane, with an average of 60 MTs maintaining more than 4μm of their length in close contact with membrane beads, until they reach the distal end of the cell (Fig. 2 *C*). Growing MTs that encounter the membrane near the centrosome bend and continue toward the distal end. Over time, these MT tips accumulate at the far end of the membrane, where they contribute to actin polymerization signaling. This results in a higher concentration of polymerization cues at the membrane region farthest from the centrosome (see Fig. S2 *C*).

In contrast, long MTs that reach the distal cell membrane may undergo catastrophe. These MTs typically have a substantial segment of their length (∼ 8μm per MT) in contact with and gliding along the cell membrane (Fig. 2 *C*). When catastrophe occurs, the MTs begin to shrink toward the centrosome, spreading contraction signals along the cortical regions they traverse. As the shrinking tips retract, they move away from the distal membrane and approach the centrosome. Because the chosen dynamic instability parameters ($f_{r}=0.04\;s^{-1}$) do not permit frequent rescue events, the contraction signals from shrinking MTs are predominantly concentrated in the cortical region near the centrosome (see Fig. S2 *C* and *D*). This process polarizes the cell, with protrusions forming at the distal membrane (farthest from the centrosome) and retraction occurring at the proximal membrane (closer to the centrosome) due to elevated cortical myosin activity. As a result, the cell migrates with the centrosome positioned posterior to the nucleus. For long MTs, the probability distribution of the angle between the cell’s direction of motion and the vector from the nucleus to the centrosome peaks near $180^{\circ }$ (Fig. 2 *D* (red line)), suggesting that the centrosome largely remains posterior to the nucleus during cell migration (5,20).

Next, we characterize the persistence of migrating cells for various average MT lengths, which correspond to different centrosome positioning. The mean-square displacement (MSD) of the cell centroid, scaled by the square of time, suggests ballistic motion ($MSD\;\propto \;t^{2}$) (Fig. 2 *E*). This indicates that the cell can move in a ballistic mode regardless of whether the centrosome is positioned anterior or posterior to the nucleus, as long as the cell remains polarized. We also examined how the persistence length of migrating cells changes with different MT lengths. For short MTs ($l_{mt}=3\;\mu m$), the mean persistence length was small (≈25μm), indicating that the cell frequently deviated from its direction of migration over short distances (Fig. 2 *F*). In contrast, the mean persistence length was significantly higher for regular and longer MTs ($l_{mt}=9.3$ and 16μm, respectively), showing that the cell was capable of directed migration with regular and long MTs. Interestingly, the persistence length was greatest for the regular MT length of $l_{mt}=9.3\;\mu m$, indicating that cells with this MT length exhibit the most consistent directionality during migration.

### Variation of MT numbers affects cell polarization and migration persistence

**Figure 3:**
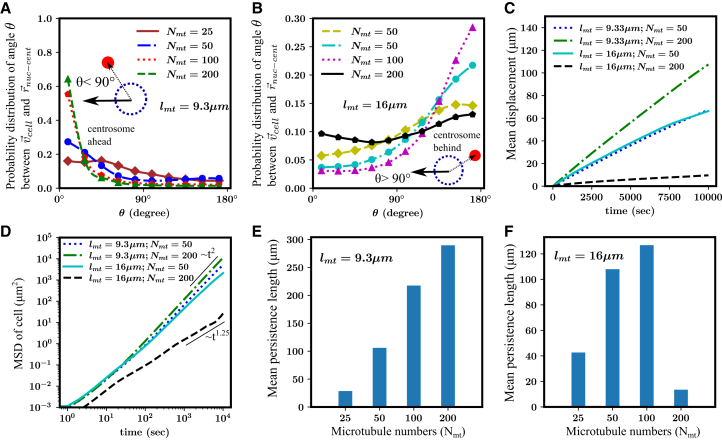
MT number affects persistence of cell migration. (*A and B*) Probability distribution of the angle between the direction of motion and the nucleus-to-centrosome vector for $l_{mt}=9.3\;\mu m$ and 16μm, for different MT numbers. (*C*) Mean displacement of the cell centroid as a function of time for varying MT numbers and average lengths. (*D*) Mean-square displacement of the cell over time for varying MT numbers and average lengths. An increase in the number of MTs, with an average MT length of $l_{mt}=16\;\mu m$, causes the cell to transition from ballistic motion ($MSD\;\propto \;t^{2}$) to super-diffusive motion ($MSD\;\propto \;t^{1.25}$). (*E*) Persistence length of migration for various MT numbers with $l_{mt}=9.3\;\mu m$. Persistence length increases steadily as the MT number increases. (*F*) Persistence length of migration for various MT numbers with $l_{mt}=16\;\mu m$. Persistence length of the cell initially increases with MT number but decreases when the MT number becomes high.

Next, we investigate how variations in MT number influence a cell’s migration characteristics. Experimental studies suggest that changes in MT abundance can either enhance or impede directed locomotion (46,47,48). For instance, increased MT numbers have been linked to greater persistence in dendritic cells and cancer metastasis (46,48). We ran simulations with varying numbers of regular and long MTs, with total MT counts ranging from $N_{mt}=25$ to 200. First, we examined the position of the centrosome for different values of $N_{mt}$ in cells containing either regular or long MTs (Fig. 3 *A* and *B*). For regular MTs, as MT numbers increase, the centrosome becomes more prominently positioned ahead of the nucleus, in the direction of migration (Fig. 3 *A*). With more MTs nucleating from the centrosome, a greater number of MTs reach the cell front, enhancing the delivery of actin polymerization signals (compare Fig. S2 *A* and *B*). Consequently, cells with more MTs exhibit increased actin polymerization activity at the front. However, due to the relatively short length of regular MTs, most are unable to circumvent the nuclear membrane and extend to the membrane region behind the nucleus. This limits the actin polymerization signals at the rear cell membrane, making cell polarization depend solely on MT growth, and independent of MT number. Our findings further show that as MT numbers increase, the angle between the cell’s direction of motion and the vector from the nucleus to the centrosome tends to be smaller (i.e., the centrosome is more often ahead of the nucleus) (Fig. 3 *A*). This suggests that in cells with regular MTs, higher MT numbers contribute to more stable protrusions at the membrane regions near the centrosome. The rear of the cell, receiving fewer polymerization signals, contracts through elastic coupling between the nucleus and the membrane. As a result, the cell tends to break symmetry and migrate, with the centrosome positioned ahead of the nucleus.

Our results demonstrate that cells with regular MTs exhibit more directed migration as the MT number increases. The displacement of the cell centroid was consistently greater for cells with $N_{mt}=200$ compared with $N_{mt}=50$ at all time points (Fig. 3 *C*). Analysis of the MSD of the cell centroid revealed ballistic movement ($MSD\;\approx t^{2}$) for cells with $N_{mt}=50-200$ (Fig. 3 *D*). Furthermore, the persistence length of migrating cells increased with MT numbers (Fig. 3 *E*), indicating that cells with more MTs maintained their polarization and were less likely to change the direction of migration. (46).

Next, we investigate the effect of MT numbers for long MTs ($l_{mt}=16\;\mu m$). For low to intermediate MT numbers ($N_{mt}=25-100$), the growing MT tips eventually reach the distal end of the membrane, where they deliver actin polymerization signals, and shrinking MTs spread contraction signals near the rear end of the cell. This establishes cell polarization through actin polymerization at the front supported by growing MT tips, and myosin contraction at the rear regulated by shrinking MT tips. When the MT number increased from 25 to 100, the probability distribution of the angle between the migration direction and the nucleus-to-centrosome vector shifts toward $180^{\circ }$ (Fig. 3 *B*), indicating a stronger cell polarization. However, when the MT number becomes very high (∼200), the polarity is reduced. A higher number of MTs leads to more growing tips occupying both the transverse and rear regions of the cell cortex, thereby reducing cell polarization. As a result, for $N_{mt}=200$, actin polymerization signals appear more dispersed rather than polarized as observed for $N_{mt}=50$ (see Fig. S2 *C* and *D*). With a large number of MTs, the cell no longer maintains a distinct anterior or posterior centrosome configuration during migration. The probability distribution of the angle between the migration direction and the nucleus-to-centrosome vector becomes more even, with no clear peak at any angle (Fig. 3 *B* (black line)). With a very large number of MTs ($N_{mt}=200$), the cell continuously shifts its front and rear based on the actin polymerization signals received along each segment of the membrane, whether proximal or distal to the centrosome (see Fig. S2 *D*).

Analysis of the cell trajectories reveals that cells with long MTs exhibit more directed migration when the MT numbers are between $N_{mt}=50-100$. The mean displacement of the cell centroid shows that cells with $N_{mt}=50$ travel greater distances over time compared with those with $N_{mt}=200$ (Fig. 3 *C*). The MSD of the cell centroid suggests that cells with $N_{mt}=50$ migrate ballistically ($MSD\;\approx \;t^{2}$), whereas cells with $N_{mt}=200$ exhibit super-diffusive motion ($MSD\;\approx \;t^{1.25}$) (Fig. 3 *D*). The persistence length of migrating cells increases with MT numbers within the range of $N_{mt}=25-100$. However, for $N_{mt}=200$, the persistence length sharply decreases to a very low value (Fig. 3 *F*). This shows that the persistence of directional locomotion increases with MT numbers for cells with long MTs ($l_{mt}=16\;\mu m$), consistent with findings from various experiments (46). However, a very large number of MTs can impair cell locomotion when the centrosome is located behind the nucleus (see Video S2).

### Anterior centrosome position improves directed migration in obstacle parks

We further examine the relationship between centrosome positioning and cell migration in obstacle parks, as explored experimentally in studies (e.g., Refs. (49,50)). These studies highlight that cells exhibiting directed migration on flat surfaces can become trapped when placed within obstacle parks. To understand how actin-MT crosstalk enables cells to navigate through restrictive geometries, we performed simulations with the cell placed in obstacle parks of varying obstacle sizes and spacings. To assess whether cell migration was directed or random, we calculated the local MSD of the cell centroid ($\Delta R^{2}\left(t_{i}\right)$) at regular time intervals of 300 s and examined its scaling exponent *α* as a function of the time lag ($\Delta R^{2}\left(t_{i},\tau_{k}\right)=A\tau_{k}^{\alpha }$) (49). Additionally, we assessed the standard deviation of the velocity angle ($\Delta \phi_{i}$) at the corresponding time intervals. Directed migration was defined as α>1.7 and $\Delta \phi_{i}<0.9$ (see supporting material for details) (49).

**Figure 4:**
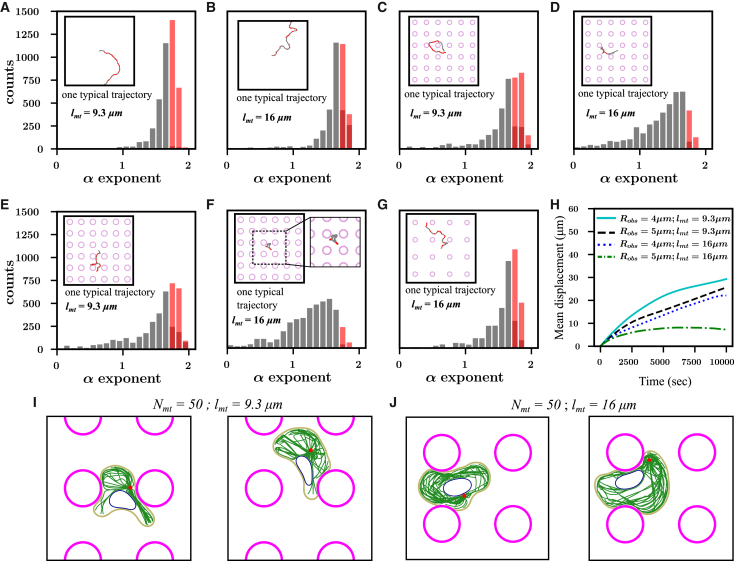
Cell migration through obstacles. (*A and B*) Distribution of local MSD exponent *α* for free cell migration for $l_{mt}=9.3\;\mu m$ and 16μm with 50 MTs. Gray bars represent counts for random migration, whereas red bars indicate counts for directed migration. (*C and D*) Distribution of exponent *α* for cell migration in obstacle park with obstacle radius $R_{obs}=4\;\mu m$ and obstacle spacing Δd=20μm for $l_{mt}=9.3\;\mu m$ and 16μm. (*E and F*) Distribution of *α* with larger obstacles having radius $R_{obs}=5\;\mu m$ and obstacle spacing Δd=20μm for $l_{mt}=9.3\;\mu m$ and 16μm. (*G*) Distribution of *α* with obstacle radius $R_{obs}=4\;\mu m$ and larger obstacle spacing Δd=30μm for $l_{mt}=16\;\mu m$. All trajectories shown in insets start at the center of the frame of size 60μm×60μm. Y-axis range is same for (*A*)–(*G*). (*H*) Mean displacement of the cell centroid over time for various obstacle sizes and MT lengths. (*I*) Snapshots of cell configuration 25 simulation minutes apart for the case shown in (*E*). Cell manages to squeeze through obstacles when centrosome is ahead of the nucleus. (*J*) Snapshots of cell configuration 25 simulation minutes apart for the case shown in (F). Cell remains stuck between obstacles when centrosome is behind the nucleus.

We first analyzed the trajectories of freely migrating cells. Our results indicate that freely migrating cells show significant counts of directed migration phases (α>1.7 and Δϕ<0.9), both for regular and long MTs (Fig. 4 *A* and *B*). When the cells were placed in an obstacle park (obstacle radius $R_{obs}=4\;\mu m$ with spacing Δd=20μm), the frequency of directed migration decreased (Fig. 4 *C* and *D*; see Video S3). However, cells with regular MTs exhibit more directed migration phases in the obstacle park compared with those with long MTs. In cells with regular MTs, the centrosome is positioned ahead of the nucleus, allowing forward-growing MTs to explore alternative paths when encountering obstacles. This enables the cells to extend protrusions into gaps between obstacles and navigate through without fully changing direction. In contrast, cells with long MTs position their nucleus ahead of the centrosome, which can block MT extension toward adjacent pores when an obstacle is encountered. As a result, cells with long MTs tend to avoid obstacles and narrow pores, leading to a significant decrease in directed migration phases. Increasing the obstacle size ($R_{obs}=5\;\mu m$) reduces the frequency of directed migration for both regular and long MT cells. However, cells with regular MTs are still able to maintain more directed migration compared with long MT cells, which are more likely to become trapped (see Fig. 4 *E* and *F*; see Video S4). On the other hand, increasing obstacle spacing to Δd=30μm leads to an increase in counts of directed migration phase (see Fig. 4 *G*). This indicates that when there is enough space for cells to pass through, they can maintain directed migration. Moreover, the mean displacement of the cell centroids revealed that cells with regular MTs, where the centrosome is positioned anterior to the nucleus, traveled greater distances in the obstacle park compared with long MT cells, regardless of obstacle size (Fig. 4 *H*–*J*).

Video S3. Cell migration in an obstacle park with obstacle spacing *d*_*obs*_ = 20 *μm* and obstacle radius *R*_*obs*_ = 4 *μm*

Video S4. Cell migration in an obstacle park with obstacle spacing *d*_*obs*_ = 20 *μm* and obstacle radius *R*_*obs*_ = 5 *μm*

### Cell morphology altered by length and number of MTs

**Figure 5:**
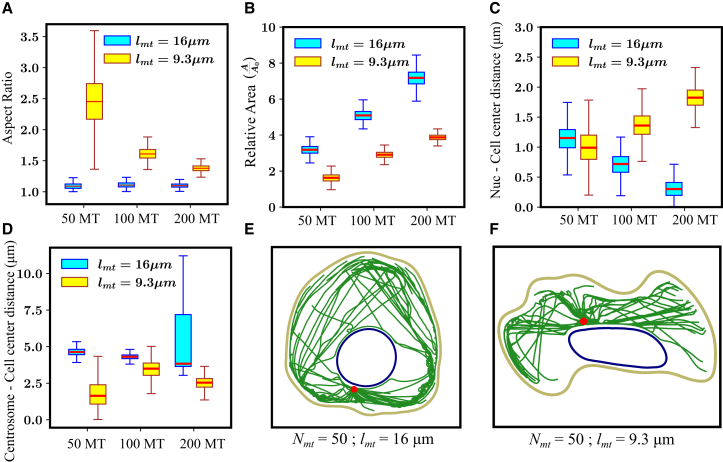
MT length and numbers influence cell morphology. (*A*) Aspect ratio of cells with various MT lengths and numbers. Cells with long MTs have an aspect ratio ∼1 (*circular shape*). Regular MTs having $l_{mt}=9.3\;\mu m$ lead to a high aspect ratio when the MT number is low ($N_{mt}=50$). (*B*) Area of the migrating cell relative to initial area ($A_{0}=\pi R_{cell}^{2}$) for various MT lengths and numbers. Cell area increases monotonically with MT numbers and average length. (*C*) Distance between the nucleus and the cell center for varying MT lengths and numbers. (*D*) Distance of centrosome from cell center for various MT lengths and numbers. (*E and F*) Snapshots of migrating cells for $l_{mt}=9.3\;\mu m$ and 16μm with $N_{mt}=50$.

Next, we examined how the actin-MT crosstalk influences the morphology of the migrating cells, quantified by the cell’s aspect ratio and spread area. When MTs are long ($l_{mt}=16\;\mu m$), the aspect ratio remains close to 1 for all MT numbers, suggesting the cell shape is nearly circular (Fig. 5 *A*). In this configuration, the centrosome is preferentially positioned behind the nucleus, near the rear of the cell. MT tips extend along both the nuclear and cell membranes, reaching the opposite end of the cell, where they deliver actin polymerization signals that drive membrane protrusions. The region closer to the centrosome has a higher density of MTs, helping to resist membrane contraction. This balance leads to a roughly circular cell shape. However, when the average MT length is shorter (e.g., regular MT length with $l_{mt}=9.3\;\mu m$), the aspect ratio varies between 1.5 and 3.5 for $N_{mt}=50$ (Fig. 5 *A*). As MT numbers increase ($N_{mt}=100,200$), the aspect ratio stabilizes around 1.5. This indicates that the cell shape deviates from a circular form when $l_{mt}=9.3\;\mu m$. In this scenario, the centrosome remains positioned ahead of the nucleus, closer to the front of the cell membrane. However, the MTs fail to navigate around the nuclear membrane to reach the opposite end of the cell. Consequently, actin polymerization signals are not delivered to the membrane region behind the nucleus. Thus, membrane protrusions only occur at the front and side regions of the cell membrane, where MTs are able to grow. In contrast, the rear of the membrane receives fewer MTs and experiences contraction forces from the membrane-nucleus coupling. The combination of extension at the front and sides, alongside contraction at the rear, leads to the cell deviating from its circular shape.

Next, we investigated the spread area of the cells for various MT numbers and lengths. Actin polymerization activity at the cortex, driven by MT delivered polymerization signals, increases the spread area of the cell. Our results indicated that cells with long MTs have a larger spread area compared with cells with regular MTs (Fig. 5 *B*). Regular MTs deliver actin polymerization signals only at the membrane regions close to the centrosome. However, long MTs deliver actin polymerization signals throughout the cell membrane, resulting in longer protrusions and increased spread area of the cell. As MT numbers increase, the actin polymerization signals at the actin cortex also increase. Thus, an increase in MT numbers corresponds to an increase in cell area for regular and long MTs.

Next, we analyzed the position of the nucleus relative to the cell center. For cells with regular MTs, the nucleus remains closest to the cell center when $N_{mt}=50$ (Fig. 5 *C*). As the number of MTs increases, the distance between the nucleus and the cell center also increases. In these cells, the centrosome is positioned anterior to the nucleus. As the MT count rises, the pushing force exerted on the nucleus grows stronger, shifting the nucleus toward the rear of the cell and away from the center (Fig. 5 *C*). In contrast, for cells with long MTs, the nucleus moves closer to the cell center as the number of MTs increases (Fig. 5 *C*). In these cases, the centrosome remains positioned posterior to the nucleus. With more MTs, the increased pushing force on the nucleus results in its movement toward the cell center.

Finally, we examine the distance of the centrosome from the cell center. For cells with regular MTs, the centrosome remains closer to the cell center (Fig. 5 *D*). When the centrosome is positioned anterior to the nucleus, our results indicate that it tends to stay near the cell center. The nucleus is slightly displaced toward the rear of the cell due to the pushing forces exerted by the MTs (Fig. 5 *E*). Many regular MTs buckle at the cell membrane, and the resulting forces push the centrosome toward the cell center. For cells with long MTs, most of the MTs slide along the cell or nuclear membrane to the cell front. Consequently, the resultant pushing force on the centrosome is insufficient to keep it near the center. As a result, the centrosome remains toward the rear of the cell, whereas the nucleus moves closer to the center (Fig. 5 *D* and *F*). As the number of long MTs increases, the pushing forces on the centrosome intensify, causing it to shift slightly closer to the center at $N_{mt}=100$ as compared with $N_{mt}=50$. However, with further increase in MT numbers, the cell loses its polarity, and the centrosome’s position becomes more random within the cell.

### MT actomyosin crosstalk influences cell path at Y-junctions

**Figure 6:**
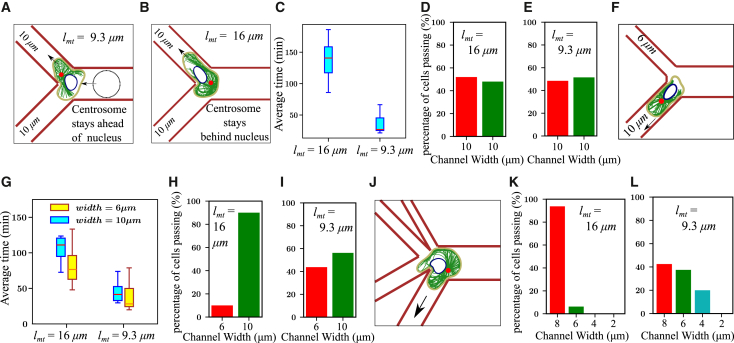
Cell migration in a Y-shaped channel (*A and B*) Snapshots show cells navigating Y-junction channels of equal width (10μm) for $l_{mt}=9.3\;\mu m$ and 16μm. In (*A*), the dotted line marks the initial cell position; arrows indicate migration direction. (*C*) Time required for cells with $N_{mt}=100$ to traverse the junction. (*D*) Percentage of cells with long MTs ($l_{mt}=16\;\mu m$) passing through each 10μmchannel. (*E*) Percentage of cells with regular MTs ($l_{mt}=9.3\;\mu m$) choosing each 10μmchannel. (*F*) Snapshot of a cell encountering a wide (10μm) and narrow (6μm) pore at the junction. (*G*) Migration time for cells with different MT lengths entering wide or narrow pores. (*H*) Percentage of cells with long MTs migrating through wide and narrow pores. (*I*) Percentage of cells with regular MTs selecting wide or narrow pores. (*J*) Snapshot of a cell in a device offering four channels of varying widths. (*K*) Percentage of cells with long MTs passing through each pore size. (*L*) Percentage of cells with regular MTs selecting among the different pore widths. All simulations were performed for $N_{mt}=100$.

Inspired by experimental observations linking cell migration paths to centrosome positioning at Y-junctions (21), we placed our model cell in a comparable setup, as depicted in Fig. 6 *A* and *B*. To replicate the chemotactic gradient applied in the experiments to drive forward migration, we impose a small forward velocity on the membrane beads. Initially, the channel widths are kept equal, larger than the nucleus diameter ($R_{nuc}=3\;\mu m$), but smaller than the cell diameter ($R_{cell}=7\;\mu m$), set at 10μm. We consider that a cell has moved into a channel when the entire cell has traveled at least 5.0μm into a channel. Our results show that migrating cells with long MTs and the centrosome positioned behind the nucleus take significantly longer to enter the channel compared with cells with regular MTs and the centrosome ahead of the nucleus (Fig. 6 *C*). Cells with regular MTs enter the channel with the centrosome positioned ahead of the nucleus. MTs growing from the centrosome can reach the protrusion tip without obstruction, enhancing actin polymerization and promoting faster entry into the channel (see Fig. S3 *A*). In contrast, cells with long MTs have the centrosome located behind the nucleus, which enters the channel first (see Fig. S3 *B*). The nucleus obstructs the entry of newly nucleated MTs into the protrusion, resulting in reduced actin polymerization at the front and slower overall entry into the channel. For these equal-width channels, the probability of moving into either edge of the Y-junction is close to 50% for both regular and long MTs (Fig. 6 *D* and *E*), and for regular MTs, the cell always took the path that the centrosome entered first, in agreement with the experimental observations (21).

Next, we simulated cells facing a Y-junction with wide (10μm) and narrow (6μm) channels (Fig. 6 *F*). Our results indicate that cells with long MTs take more time to move into either the narrow or wide channel compared with cells with regular MTs. However, when the width of the two channels is unequal, the time taken by cells with long MTs to choose a channel decreases (compare Fig. 6 *C* and *G*). This suggests that when one path is much more restrictive than the other, cells with their nucleus ahead of the centrosome retract their protrusion from the restrictive path faster. When both the path choices are similar, the cell takes more time to retract its protrusion from one path and move completely into the other channel.

For migrating cells with long MTs ($l_{mt}=16\;\mu m$) and the nucleus ahead of the centrosome, cells prefer to move into the wider channel (Fig. 6 *H*). This behavior is consistent with experimental observations suggesting that cells use their nucleus to gauge pore sizes and choose the path of least resistance (21). Cells with regular MTs ($l_{mt}=9.3\;\mu m$) and the centrosome positioned ahead of the nucleus exhibit a higher tendency to enter the narrow pore compared with cells with long MTs (Fig. 6 *I*). This result is in qualitative agreement with experimental observations of fibroblasts and dendritic cells navigating bifurcating channels of different widths (21). However, these cells still have a higher probability of moving into the wider pore over the narrower one (Fig. 6 *I*). This may be attributed to the fact that, with the centrosome ahead of the nucleus, growing MTs can easily extend into the protrusion within the smaller channel, facilitating actin polymerization. In contrast, cells with long MTs, where the nucleus is positioned ahead of the centrosome, experience blockage from the nucleus, preventing the MTs from reaching the smaller channel. As a result, membrane protrusions in the smaller channel are less stabilized and eventually retract.

Finally, we examined the behavior of cells at a junction with multiple path choices. We simulated cells with varying MT lengths within a geometry containing four paths, each with different channel widths (Fig. 6 *J*). Our results indicate that cells with long MTs ($l_{mt}=16\;\mu m$) explore all available paths and only enter channels with wider pores (channel width = 8μm and 6μm), whereas no cells moved into channels with very narrow pores (channel width = 4μm and 2μm) (Fig. 6 *K*; see Video S5). The majority of the cells (≈90% ) moved into the widest pore (width = 8μm), indicating a clear preference for the path of least resistance. In contrast, cells with regular MTs ($l_{mt}=9.3\;\mu m$) did not show a strong preference for the widest pore, with about ≈40% of cells moving into the 6μm channel (Fig. 6 *L*; see Video S5). Nearly 20% of cells moved into the 4μm channel, whereas no cells moved into the 2μm channel. These results suggest that with the centrosome positioned ahead of the nucleus, MTs can extend further into smaller pores, stabilizing protrusions and enabling the cell to squeeze through. Similar path selection behavior, consistent with our simulation results, has been observed in experimental studies across different cell types, where the MTOC is positioned either ahead of or behind the nucleus at channel junctions (21).

Video S5. Cell migration in path choice device with four channels of width: 2 *μm*, 4 *μm*, 6 *μm*, and 8 *μm*

## Conclusion

Motivated by recent experiments reporting strong correlations between centrosome positioning and migration characteristics and path choices, we presented in this work a mechanistic whole-cell model that integrates basic aspects of actin-MT crosstalk, namely growing (shrinking) MTs delivering polymerization (contraction) signals locally to actomyosin. Our model shows that the position of the centrosome, anterior or posterior to the nucleus, corresponds to different arrangements of the MT array within the cell. This provides different pathways for cell polarization through actin polymerization and myosin contraction signals. Our in silico results indicate that the position of the centrosome depends on the average lengths of the MTs (Fig. 2 *B*). Regular MTs, with an average length of two-thirds of the cell diameter, place the centrosome mostly ahead of the nucleus, whereas long MTs with an average length greater than the cell diameter place the centrosome behind the nucleus in the direction of migration.

The migrating cell can also change from ballistic to diffusive motion by adjusting its cortical actin dynamics through changes in the distribution of regulatory signals reaching the cortex. Earlier studies have reported that variations in MT numbers can alter the characteristics of cell migration (46,47,51). Our model also suggests that changes in the number of MTs can lead to different cell migratory behaviors. In a centrosome anterior to the nucleus configuration, an increase in the number of MTs leads to a significant increase in persistence (Fig. 3 *D*). In the centrosome posterior to the nucleus configuration, increasing MT numbers initially lead to ballistic migration, but the motion becomes super-diffusive when the number of MTs is too high (Fig. 3 *C*).

When migrating through complex tissue geometries, cells encounter obstacles formed by the surrounding extracellular matrix and neighboring tissues. At junctions, cells develop highly branched morphologies, extending protrusions to probe narrow channels and select an optimal path. Eventually, one protrusion stabilizes and guides the cell, whereas others are retracted (52). MTs are crucial in this selection: the successful protrusion is reinforced by MT-mediated delivery of actin polymerization signals, whereas competing protrusions retract following cortical contraction cues (6). Experimental studies have shown a marked increase in cell passage time and instances of cell fragmentation with disintegration of protrusions from cell body at Y-channel junctions after complete MT depolymerization (21). Complete depolymerization of MTs in our model caused migratory failure, and cells remained stuck at channel junctions (see Fig. S4 *A*–*C*).

Experimental studies on *Dictyostelium discoideum* and leukocytes navigating pillar arrays and Y-junctions suggest that centrosome positioning, either anterior or posterior to the nucleus, influences path choice (20,21). Cells also tend to migrate away from regions densely packed with obstacles (53). Our simulation results predict that with the centrosome anterior to the nucleus, cells could migrate more robustly in obstacle parks (Fig. 4 *C*). At a Y-junction, cells with the centrosome positioned anterior to the nucleus show only a slight preference for the wider channel, whereas cells with a posterior centrosome almost always migrate through the wider channel (Fig. 6 *G*). We also observed that increased cell membrane stiffness and a softer nucleus can improve cell migration in obstacle parks (see Fig. S5). Further, cells in obstacle parks were found to migrate toward regions of sparse obstacle density (see Fig. S6).

Our results indicate that changes in the physical properties of MTs, such as their average length and number, can influence cellular migration. Future experimental studies investigating how cells regulate MT organization to adapt their migratory behavior in both unconfined and confined geometries would provide valuable insights into this mechanism.

Our results offer insights into how immune cells navigate tissues, how cancer cells start to metastasize, and how cells migrate within synthetic confined geometries. This model represents an initial step toward integrating key aspects of actin-MT crosstalk into a mechanistic framework for cell migration, and it already captures a range of self-organized behaviors, including centrosome positioning, transitions between random and ballistic motion, and path selection in confined spaces. However, the model involves several simplifications: for instance, the closed-loop bead-spring representation of the membrane does not fully capture the complexity of real membranes, and parameters such as stretching and buckling stiffness cannot be directly mapped to experimental measurements. Similarly, dynein-MT interactions at the cortex are simplified through spring attachments between discrete beads. To match experimental observations, we selected parameter values that best reproduced known behaviors.

Our study focuses on the role of MT dynamics in establishing and maintaining cell polarization, as well as guiding path selection in both free and confined geometries when the cell encounters obstacles. The simulation timescales were chosen to effectively capture MT-driven processes relevant to polarization and path guidance under these conditions. However, the simulations do not capture the transition from ballistic to diffusive migration observed over longer timescales. Modeling this transition would require significantly more extensive and computationally demanding simulations.

Our model assumes a constant average MT length, which does not fully align with experimental observations. In dendritic cells, where the centrosome is positioned behind the nucleus, polarization is supported by longer, more stable MTs extending toward the leading edge and shorter, less stable MTs oriented toward the rear (6,13). This organization produces an elongated, elliptical morphology that our model cannot capture, as it lacks a mechanism for stable polarization with an asymmetric MT length distribution. Experimental studies have demonstrated feedback between actin polymerization and growing MT tips at the leading edge of migrating cells (54). MTs can be anchored to the actin cortex at the front or pushed back as a result of actin retrograde flow during polymerization. Our model does not incorporate mechanical anchoring of MT tips at the leading edge or the pushing forces exerted by actin retrograde flow. Including such feedback mechanisms could influence the spatial distribution of MT tips at the cell cortex.

Our model uses a fixed number of beads for the cell and nuclear membranes, along with a relatively large stiffness parameter between adjacent beads. This limits large variations in cell perimeter. Fibroblasts and neutrophils have been observed to maintain a relatively constant contact area and perimeter on soft substrates (55,56). However, their contact area and perimeter can increase significantly on stiff substrates (55,56,57). Our present model does not capture such large deviations in cell perimeter.

MT-mediated chemical signaling is not the sole pathway for cell polarization. Alternative mechanisms enabling polarization and subsequent migration in the absence of MTs have also been reported in the literature, which have not been accounted for in our model (58,59). Investigating mechanisms that enable such stable polarization and incorporating them into the model would be an interesting direction for future work.

Cells are also known to exhibit variations in MT stability in response to external chemical or mechanical cues (6,13,60,61). Moreover, factors such as the arrangement of the extracellular matrix and the density of adhesion molecules influence migratory behavior (62,63,64). Cells migrating in 3D extracellular matrix environments often display different characteristics compared with those migrating on 2D surfaces, a distinction that simple 2D models cannot fully represent. Future studies could explore how cells migrating in both 2D and 3D environments sense external cues, such as chemical gradients and matrix organization, to guide their migration. Understanding the role of MTs and actin-MT crosstalk in these sensing mechanisms would be an interesting area for further investigation.

## Acknowledgments

P.N. was supported by a fellowship from 10.13039/501100001412CSIR, India. H.R. acknowledges financial support by the 10.13039/501100001659German Research Foundation (10.13039/501100001659DFG), project number 468346334. R.P. thanks 10.13039/501100024236IACS for funding and computational facilities.

## Author contributions

H.R. conceived the study; P.N., A.K.D., R.P., and H.R. designed research and developed computational models; P.N. performed simulations; P.N., A.K.D., R.P., and H.R. analyzed data and wrote the paper. All authors contributed to editing and revisions of the paper.

## Declaration of interests

The authors declare no competing interests.

## Supporting citations

References (65,66,67,68,69,70,71,72,73,74,75,76,77,78,79,80,81,82,83,84) appear in the supporting material.

## Supporting material

Document S1. Figures S1–S6 and Table S1

Document S2. Article plus supporting material

## Footnotes

Supporting material can be found online at https://doi.org/10.1016/j.bpj.2025.09.029.
