# Supplementary material for: Modeling actin-microtubule crosstalk in migrating cells
Source: Biophys J. 2025 Sep 23;124(21):3742–54. doi: 10.1016/j.bpj.2025.09.029 (PMC12709440; doi:10.1016/j.bpj.2025.09.029)
Supplement: Document S1. Figures S1–S6 and Table S1 [file mmc1.pdf]

Biophysical Journal, Volume *124*

Supplemental information

Modeling actin-microtubule crosstalk in migrating cells

Pinaki Nayak, Anil Kumar Dasanna, Raja Paul, and Heiko Rieger

Supplemental Information

SUPPLEMENTAL FIGURES AND LEGENDS

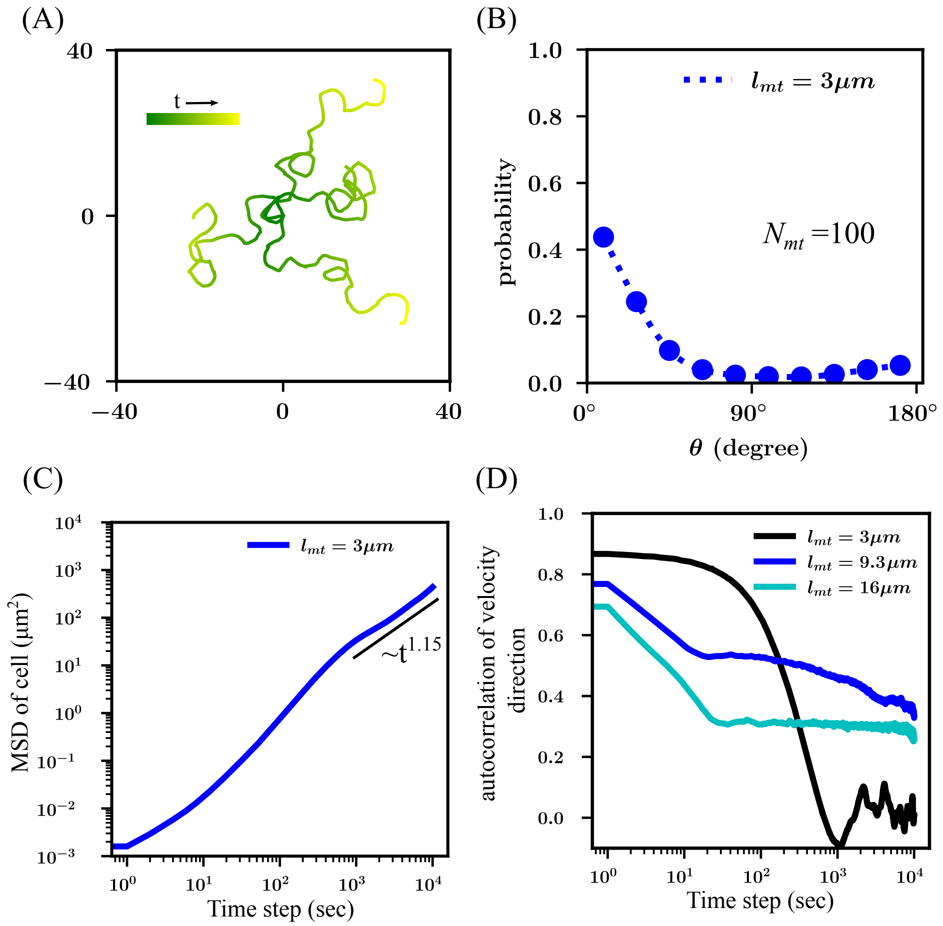

Figure S1. Short MTs frequently cause cells to change migration direction. **(A)** Trajectories of cell centroids with short average MT length of $l_{mt} = 3\mu m$ at an intermediate membrane stiffness of $k_{mem} = 3 \times 10^{-16}$J and MT stiffness of $k_{mt} = 5 \times 10^{-16}$J. **(B)** Probability distribution of angle between direction of cellular motion and nucleus to centrosome vector for $l_{mt} = 3\mu m$. **(C)** Mean square displacement of cells showing a $t^{1.15}$ dependence corresponding to super diffusive migration. **(D)** Velocity direction autocorrelation function shows velocity direction becomes uncorrelated with time for $l_{mt} = 3\mu m$.

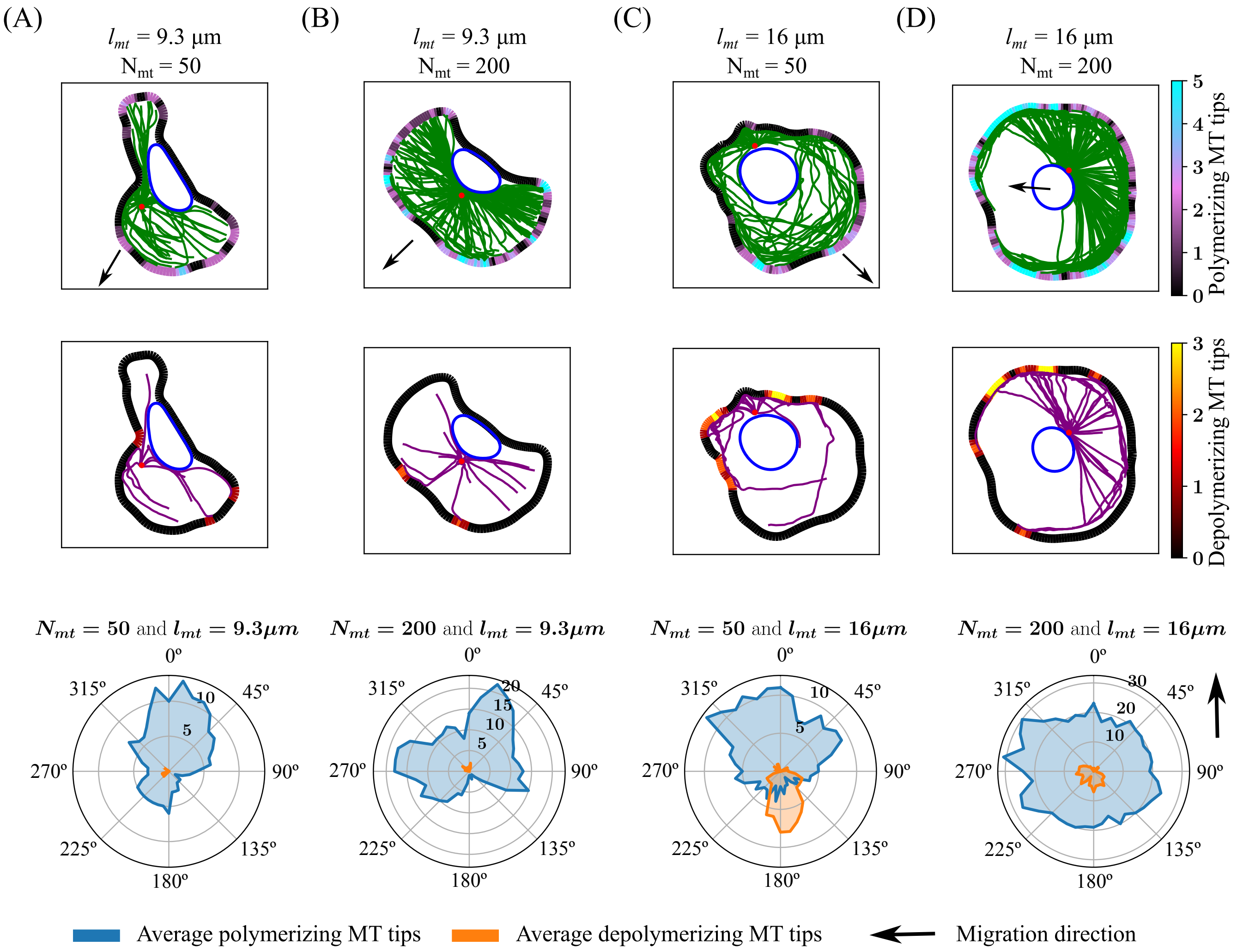

Figure S2. Distribution of growing and shrinking MT tips along the cell membrane : (A) Snapshots and distribution of growing and shrinking MT tips along the membrane for $N_{mt} = 50$ and $l_{mt} = 9.3\mu m$. **(B)** Snapshots and distribution of growing and shrinking MT tips along the membrane for $N_{mt} = 200$ and $l_{mt} = 9.3\mu m$. For regular MTs, the growing and shrinking MTs deposit polymerization and contraction signals mostly at the membrane region closer to the centrosome. **(C)** Snapshots and distribution of growing and shrinking MT tips along the membrane for $N_{mt} = 50$ and $l_{mt} = 16\mu m$. **(D)** Snapshots and distribution of growing and shrinking MT tips along the membrane for $N_{mt} = 200$ and $l_{mt} = 16\mu m$. For long MTs, the growing MT tips move to the region of the membrane away from the centrosome and shrinking MT tips are abundant along the membrane region closer to the centrosome.

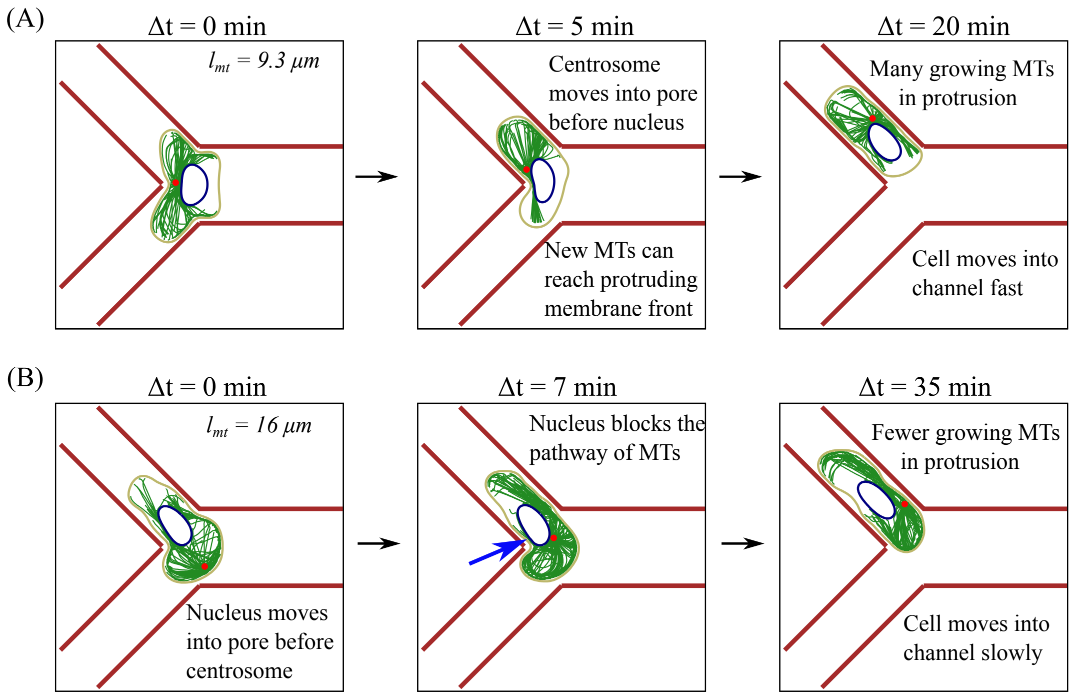

Figure S3. Migration of cell in Y channel : (A) Snapshots of cell moving into a symmetric Y channel with $l_{mt} = 9.3\mu m$. Centrosome is ahead of nucleus and new MT tips can reach protrusion tip without obstruction. (B) Snapshots of cell moving into a symmetric Y channel with $l_{mt} = 16\mu m$. Nucleus moves into the channel ahead of centrosome and obstructs new MT tips from reaching the protrusion tip.

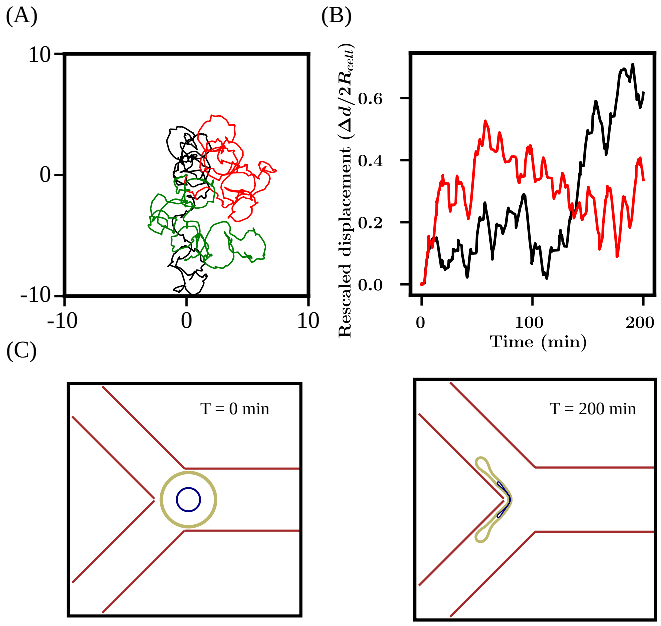

Figure S4. Very short or no MTs lead to migratory failure. (A) Typical trajectories of cell centroids with very short MTs ($l_{mt} = 1.0\ \mu m$) for $0 - 200\ min$. All trajectories start at the origin (0,0). **(B)** Cell centroid displacement from initial position scaled by $2R_{cell}$. **(C)** Initial and final configuration of cell without MTs at a Y junction channel. Loss of MTs leads to cell membrane collapse at Y junction.

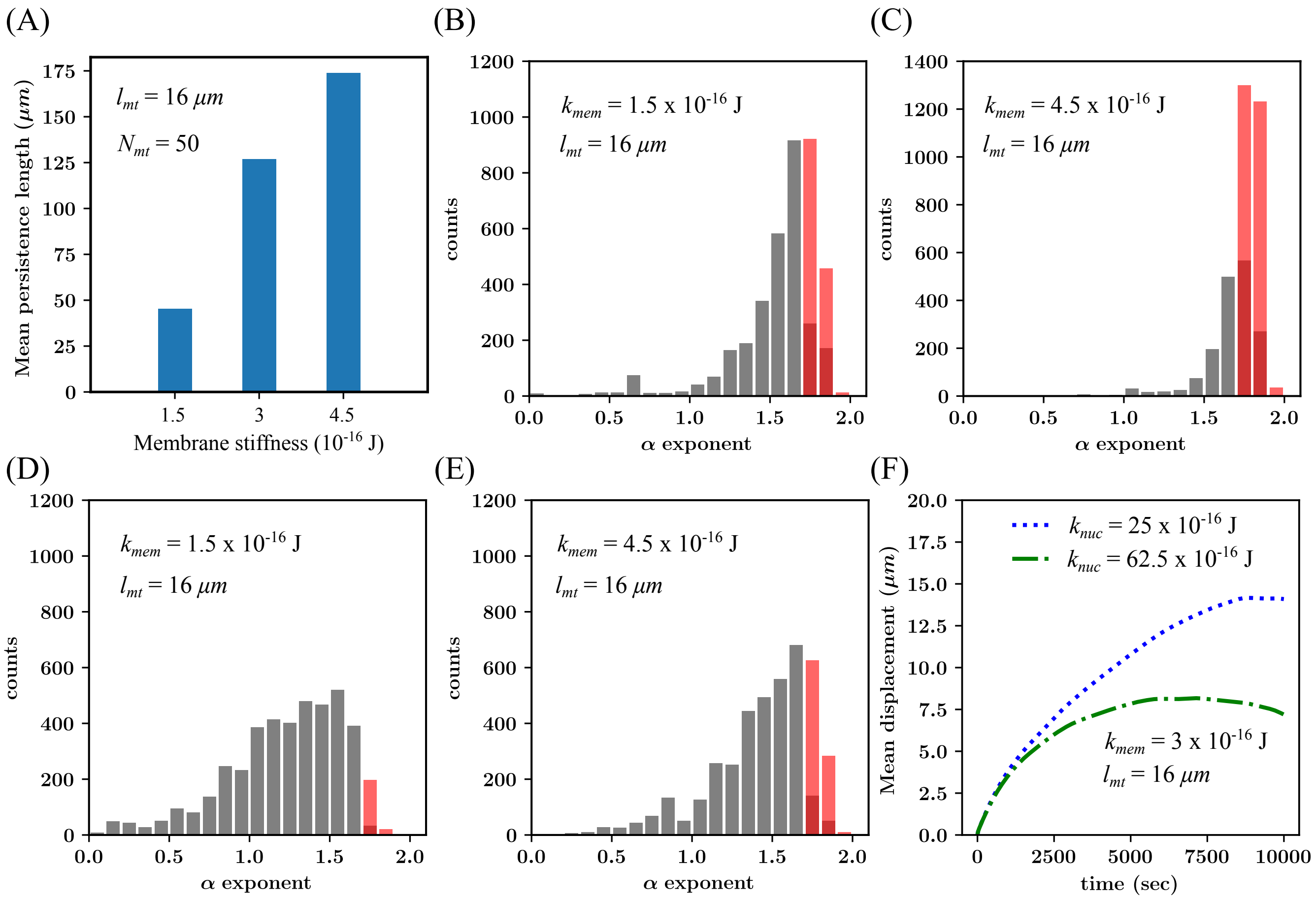

Figure S5. Membrane stiffness affects cell persistence. **(A)** Persistence length of cells with varying membrane stiffness for centrosome posterior to nucleus ($l_{mt} = 16\mu m$). **(B)** Distribution of local MSD exponent α for freely migrating cells with long MTs and $k_{mem} = 1.5 \times 10^{-16}$J. **(C)** Distribution of α for freely migrating cells with long MTs and $k_{mem} = 4.5 \times 10^{-16}$J. **(D)** Distribution of α for migrating cells in obstacle maze with long MTs and $k_{mem} = 1.5 \times 10^{-16}$J. **(E)** Distribution of α for migrating cells in obstacle maze with long MTs and $k_{mem} = 4.5 \times 10^{-16}$J. **(F)** Mean displacement of cells migrating in obstacle maze with long MTs and varying nucleus stiffness. All simulations were performed for $N_{mt} = 50$.

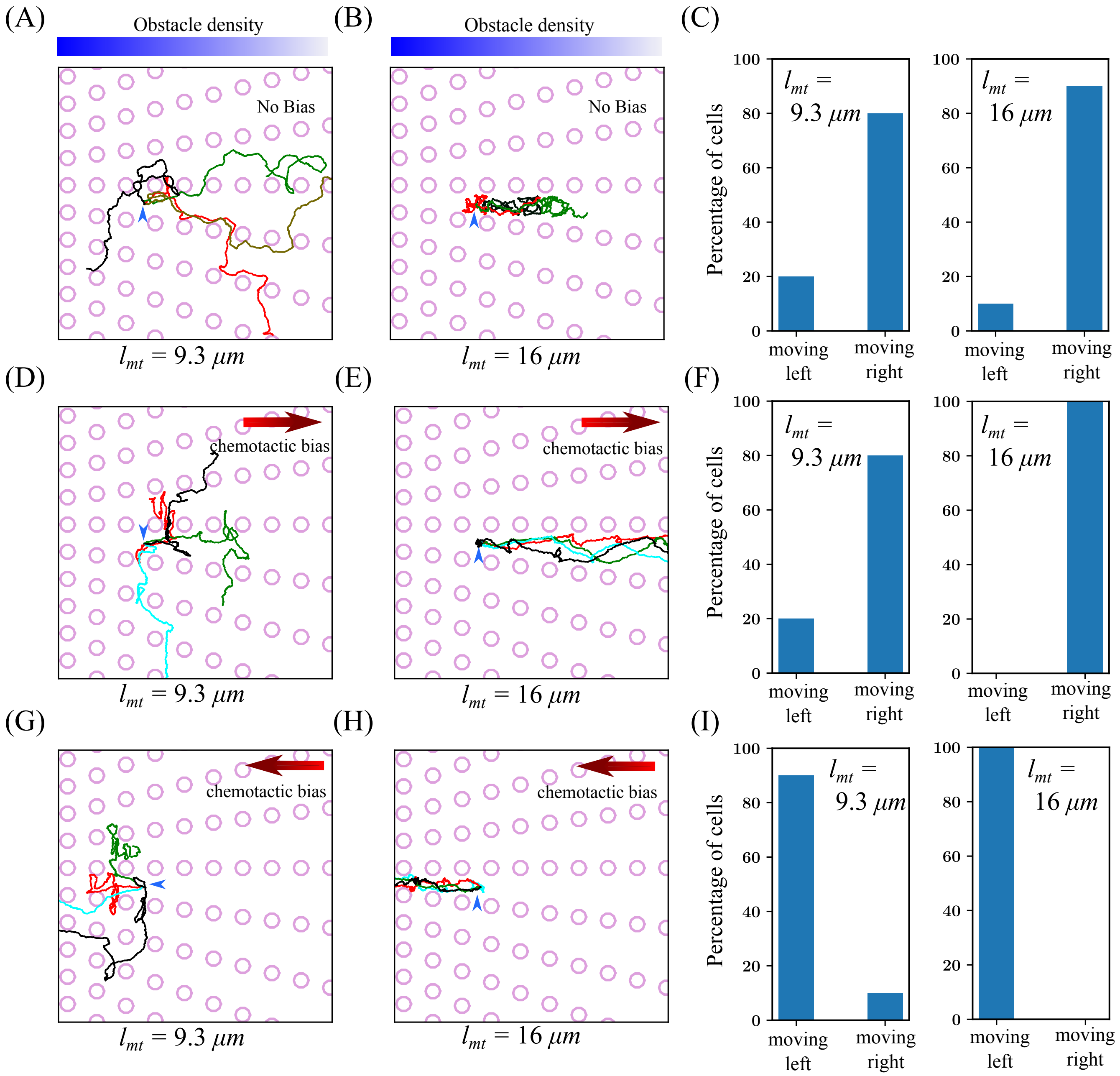

Figure S6. Cell migration in obstacle park with obstacle density gradient (A) Cell trajectories for cells with regular MTs and N_{mt} = 50, placed in an obstacle park with gradient obstacle density. **(B)** Cell trajectories for cells with long MTs in obstacle park with gradient obstacle density. **(C)** Percentage of cells moving left (towards higher obstacle density region) and cells moving right (lower obstacle density region) for regular and long MTs. **(D)** Cell trajectories for cells with regular MTs and N_{mt} = 50 in presence of a chemotactic bias towards the right (lower obstacle density region). **(E)** Cell trajectories for cells with long MTs and a chemotactic bias towards the right (lower obstacle density region). **(F)** Percentage of cells moving left and cells moving right for regular and long MTs in presence of chemotactic bias towards the right. **(G)** Cell trajectories for cells with regular MTs and a chemotactic bias along the left (higher obstacle density region). **(H)** Cell trajectories for cells with long MTs and a chemotactic bias along the left (higher obstacle density region). **(I)** Percentage of cells moving left and cells moving right for regular and long MTs, with a chemotactic bias towards the left (higher obstacle density region). Blue arrow indicates the initial position of the cell.

SUPPLEMENTAL TABLES

Symbol	Description	Value	Reference
N_m	Number of cell membrane beads	200	(1)
N_n	Number of nuclear membrane beads	200	(1)
N_{mt}	Number of MTs	50 - 200	(2–5)
N^m_{dyn}	Number of dynein motors on cell membrane	400	This study
N^n_{dyn}	Number of dynein motors on nuclear membrane	400,600	This study
R_{cell}	Initial radius of cell	7 μm	(6)
R_n	Initial radius of nucleus	3 μm	(7)
v_g	MT growth velocity	0.4 $\mu m s^{-1}$	(8, 9)
v_g	MT shrink velocity	0.8 $\mu m s^{-1}$	(9, 10)
f_c	MT catastrophe frequency	0.045-0.06285 s^{-1}	(10, 11)
f_r	MT rescue frequency	0.04 s^{-1}	(10, 11)
k_{mem}	Cell membrane stiffness	1.5-4.5 $\times 10^{-16}$J	(12, 13)
k^m_θ	Cell membrane bending rigidity	6.25 $\times 10^{-16}$J	(12)
l^m_0	Rest length of membrane spring attachments	0.22 μm	This study
ϵ_m	Strength of LJ interaction between membrane-nucleus	312.5 $\times 10^{-18}$J	This study
k_{nuc}	Nuclear membrane stiffness	$25 - 62.5 \times 10^{-16}$J	This study
k^n_θ	Nuclear membrane bending rigidity	3.125×10^{-16}J	This study
$2^{\frac{1}{6}}\sigma_m$	Cut-off distance for membrane-nucleus interaction	0.31μm	This study
k_{mt}	MT stretching stiffness	5 $\times 10^{-16}$J	(14)
k^{mt}_θ	MT bending rigidity	0.28 $\times 10^{-18}$J	(14)
l^{mt}_0	Rest length of MT spring attachments	0.2 μm	(15)
ϵ_{mt}	Strength of LJ interaction between MT-Cell/Nucleus	312.5 $\times 10^{-18}$J	This study
$2^{\frac{1}{6}}\sigma_{mt}$	Cut-off distance for MT-Cell/Nucleus LJ interaction	0.42μm	This study
k_d	Dynein spring attachment strength	0.125 $\times 10^{-18}$J	This study
v_{dyn}	Dynein walking speed on MTs	40$nm s^{-1}$	(16)
d_{cut}	Dynein attachment cut-off distance	1μm	(15)
P_{dyn}	Dynein attachment probability	0.5	This study
P_{turn}	Dynein turnover probability	0.5	This study
k_c	Nucleus to cell membrane linear coupling strength	0.3125$\times 10^{-18}$J	This study
$l_p S_0 r_0$	Actin polymerization velocity in absence of polymerization signals	0.1$\mu m s^{-1}$	This study
C_p	Actin polymerization velocity increment per growing MT tip	0.1$\mu m s^{-1}$	This study
F_0	Cortical acto-myosin contraction force in absence of contraction signals	40-70 pN	This study
C_d	Cortical contraction force increment per shrinking MT tip	70 pN	This study
ζ_c	Effective coefficient of viscosity of cell membrane beads	6.25$\times 10^{-4} N m^{-1} sec$	This study
ζ_n	Effective coefficient of viscosity of nucleus membrane beads	8$\times 10^{-4} N m^{-1} sec$	This study
ζ_{mt}	Effective coefficient of viscosity of MT beads	1.5$\times 10^{-4} N m^{-1} sec$	This study
ζ_{mt}	Effective coefficient of viscosity of centrosome	3$\times 10^{-4} N m^{-1} sec$	This study

Table S1: **Parameter values used in simulations.** The characteristic length scale was chosen as $l_0 = 0.283\mu m$ to evaluate the spring constant energies ($k_{energy} = \frac{1}{2} k l_0^2$).

SUPPORTING MOVIES

Movies depicting cell migration in free and restricted environments.

Video S1 Persistent cell migration for $N_{mt} = 100$ and $l_{mt} = 9.3\mu m$; Persistent cell migration for $N_{mt} = 50$ and $l_{mt} = 16\mu m$.

Video S2 Diffusive cell migration for $N_{mt} = 100$ and $l_{mt} = 3\mu m$; Diffusive cell migration for $N_{mt} = 200$ and $l_{mt} = 16\mu m$.

Video S3 Cell migration in an obstacle park with obstacle spacing $d_{obs} = 20\mu m$ and obstacle radius $R_{obs} = 4\mu m$.

Video S4 Cell migration in an obstacle park with obstacle spacing $d_{obs} = 20\mu m$ and obstacle radius $R_{obs} = 5\mu m$.

Video S5 Cell migration in path choice device with 4 channels of width $2\mu m$, $4\mu m$, $6\mu m$, $8\mu m$.

SUPPLEMENTAL MATERIALS AND METHODS

Short MTs lead to loss of persistence

To test the effect of shortened MTs on cell polarization, cells were simulated with a short average MT length of $l_{mt} = 3\mu m$. The cell centroid trajectories revealed that the cell became increasingly prone to turning, leading to a loss of persistence (Fig. **S1 A**; see **Video S2**). The centrosome stayed ahead of the nucleus in the direction of migration (Fig. **S1 B**). The mean squared displacement of the cell centroid showed a scaling of 1.15, which corresponds to a super-diffusive regime (Fig. **S1 C**). Analysis of the velocity direction autocorrelation function indicated that cell migration direction was correlated at small time scales (Fig. **S1 D**). With increasing time, the correlation of cell velocity decayed steeply to zero, indicating that the cell was prone to changing the direction of propagation with time.

Distribution of MT signals at the membrane-cortex region

Within a cell containing regular MTs of length $l_{\mathrm{mt}} = 9.3\ \mu$m, the membrane region near the centrosome receives the majority of both growing and shrinking MT tips (see Fig. **S2 A** and **S2 B**). Growing MT tips promote actin polymerization, leading to membrane protrusions, while shrinking tips transmit signals for actomyosin contraction. Because shrinking tips tend to retract from the membrane due to depolymerization, their numbers along the membrane are comparatively lower. This asymmetry leads to a higher concentration of actin polymerization activity near the centrosome, thereby establishing cell polarity. Under these conditions, the cell migrates with the centrosome positioned ahead of the nucleus. Increasing the number of MTs from $N_{\mathrm{mt}} = 50$ to $N_{\mathrm{mt}} = 200$ enhances the number of polymerizing MT tips near the centrosome. However, the number of shrinking tips does not increase significantly, as they continue to recede from the membrane due to depolymerization (compare Fig. **S2 A** and **S2 B**). As a result, cell polarization becomes more pronounced with a higher number of MTs for regular-length MTs.

For long MTs ($l_{\mathrm{mt}} = 16\ \mu$m) and an intermediate MT count ($N_{\mathrm{mt}} = 50$–100), growing MT tips are predominantly located in membrane regions away from the centrosome (see Fig. **S2 C**), while shrinking tips are more concentrated near the centrosome. This distribution establishes a polarity in which actin polymerization is stronger away from the centrosome, and contractile activity is higher closer to it. Under these conditions, the cell migrates with the centrosome positioned behind the nucleus and exhibits a high persistence length. When the number of MTs is increased to $N_{\mathrm{mt}} = 200$, the region near the centrosome also receives a considerable number of growing MT tips (see Fig. **S2 D**), leading to additional actin polymerization signals in that area. This results in a reduction of cell polarity and decreased migration persistence.

MT dynamics at Y junction dictates channel selection time

When a cell with regular MTs reaches a Y-junction, the centrosome is generally positioned between the leading edge and the nucleus (see Fig. **S3 A**). Microtubules emanating from the centrosome can enter protrusions in either branch of the junction without obstruction. The winning protrusion is selected stochastically: more microtubules move into the selected protrusion, while the other loses microtubules and is subsequently retracted. As the cell advances into the selected branch, the centrosome remains ahead of the nucleus, allowing newly growing microtubules to readily reach the tip of the protrusion and deliver actin polymerization signals. This results in rapid cell passage through the chosen channel.

In cells with long MTs at a Y-junction, the centrosome is positioned behind the nucleus. Growing MTs must slide past the nucleus to reach the protrusion tips. One protrusion is selected stochastically, and the nucleus moves into the selected protrusion ahead of the centrosome (see Fig. **S3 B**). The nucleus then acts as a barrier for newly growing MTs attempting to reach the protrusion tip. As a result, the cell takes more time to fully enter the selected channel (see Fig. **6 C**).

Severely short MTs impair cell migration

The effect of MT depolymerization on the migration was investigated by simulating cells with average MT length severely shortened to $l_{mt} = 1.0\mu m$. Our results indicated that the cell failed to establish and maintain any front-back polarization with severely short MTs. Trajectories of cell centroids in Fig. **S4 A-B** indicate that the cell centroid did not have a net displacement in the order of the cell diameter throughout the simulation time. We interpret this as an overall failure of the cell migration. Events of migration failure due to severely shortened MTs have been demonstrated in nocodazole-treated cells that depolymerize MTs (17).

The functional consequence of complete MT depolymerization for cells migrating in restricted geometries was checked by simulating cells without MTs at a Y junction. A small velocity was given to each cell membrane bead directed inwards into the channel, depending on which channel the bead was in. Cells at a symmetric Y junction (channel width = $10\mu m$) failed to migrate into either channel. The cell collapsed at the Y junction in the absence of MTs (Fig. **S4 C**).

Stiffer cell membrane-actin cortex and softer nucleus improves persistence in posterior centrosome configuration

The influence of the cell membrane and underlying actin cortex stiffness on the migration of the cell was investigated by varying the membrane spring constant k_{mem} in the simulations. For cells with regular MTs ($l_{mt} = 9\mu m$), no significant change in migration characteristics were observed. However, cells with long MTs ($l_{mt} = 16\mu m$) were found to migrate more persistently in unrestricted geometries with a stiffer cell membrane (Fig. **S5 A**). Analysis of the local msd exponent α revealed that, in the centrosome posterior to nucleus configuration, a stiff cell membrane leads to more directed run phases of the cell (Fig. **S5 B, C**). A stiffer cell membrane and actin cortex help in faster retraction of the cell rear through enhanced membrane tension and result in improved directed locomotion of the cell. In obstacle parks, cells with long MTs ($l_{mt} = 16\mu m$) were found to show improved migration with a stiffer cell membrane and underlying actin cortex. This was indicated by the presence of higher counts of directed run phases for $k_{mem} = 4.5 \times 10^{-16}$J as compared with $k_{mem} = 1.5 \times 10^{-16}$J (Fig. **S5 D, E**). Finally, a softer nucleus was found to improve the capability of the cells to squeeze through narrow pores between obstacles in obstacle parks. For obstacle radius $R_{obs} = 5\mu m$ and spacing $\Delta d = 20\mu m$, cells having long MTs with $k_{mem} = 3 \times 10^{-16}$J and $k_{nuc} = 62.5 \times 10^{-16}$J found it difficult to move through the narrow pores between obstacles resulting in stuck configurations of the cell. However, when the nucleus membrane stiffness was reduced to $k_{nuc} = 25 \times 10^{-16}$J the ability of the cells to migrate through the narrow spacings between obstacles improved, resulting in increased average displacement of the cell centroid with time (Fig. **S5 F**).

Unbiased cells move towards less dense regions in obstacle parks with varying density

Finally, we investigated the behavior of migrating cells within obstacle parks with varying obstacle densities. Cells with regular and long MTs mostly moved towards the region with a sparse distribution of obstacles, with few cells moving toward the region densely packed with obstacles (Fig. **S6 A-C**). This suggested that cells can use their MT-actin crosstalk to navigate towards less restrictive regions. For cells with regular MTs, a greater percentage ($\approx 20\%$) of cells moved towards the denser region as compared with cells with long MTs ($\approx 10\%$). However, cells with regular microtubules were able to move more robustly within the obstacle park, as was also seen for regularly spaced obstacle parks (see Fig. **4**).

Next, we introduced a chemotactic bias on the cell by adding a small velocity to the cell membrane beads in the direction of the bias. The migration of cells with regular MTs was found to be hampered when the chemotactic bias was in the direction of sparsely placed obstacles (Fig. **S6 D**). This indicated that the polarization of the cell due to microtubule-delivered signals failed to align with the chemotactic bias. Therefore, the cell demonstrated a reduction in its ability to navigate through the narrow spaces between obstacles. However, most cells moved towards the sparse obstacle density region. For long MTs, all the cells moved towards the sparse obstacle density region with the migration becoming more robust (Fig. **S6 E, F**).
The direction of the chemotactic bias was then reversed towards the densely packed region. Cells with regular microtubules mostly moved towards the densely packed region (Fig. **S6 G**). A small percentage ($\approx 10\%$) of cells moved towards the sparsely packed region opposite to the applied chemotactic gradient. Cells with long MTs only moved towards the densely packed region, indicating that long MT cells can align themselves better to the applied chemotactic gradient (Fig. **S6 H, I**).

DETAILS OF THE MODEL

A mechanistic model of cell migration primarily involves membrane protrusion and retraction, which are coupled to the actomyosin cortex and actin cytoskeleton. This process includes actin polymerization, myosin-driven membrane contraction, polarization of actin filaments, focal adhesion kinetics, and membrane surface tension. Research has shown that MT

depolymerization regulates actomyosin contraction through the modulation of Rho GTPase signaling pathways (18). On the other hand, MT polymerization can regulate actin polymerization and the expansion of protrusions by transporting intracellular cargo and signaling molecules to the leading edge of migrating cells (18–20). We use these two observations as key components to develop a mechanistic whole-cell model of MT-actin crosstalk during cell migration.

We focus on two-dimensional mesenchymal cell migration, using the following basic model elements: 1) bead-spring loops to represent the semi-flexible boundary of the cell and its (circular) nucleus, 2) bead-spring semi-flexible polymers for dynamic MTs (MTs), which can grow and shrink at their plus ends through the addition and removal of beads, respectively. These MTs are anchored at the MT organizing center (MTOC), which we assume to coincide with the cell's centrosome, and 3) growing MTs exert pushing forces on the membrane when they come into contact, while dyneins, anchored at the cell and nucleus membranes, can attach to MTs and generate pulling forces on them. A sketch of these standard parts of our model (c.f. (21, 22)) is shown in Fig. **1**). As new model elements, we incorporate MT-actin crosstalk by introducing a regulatory cue that modulates increased actin polymerization at the cortex or membrane contraction due to myosin-II activity, which is locally delivered to the cortex at the MT tips. Shrinking MTs deliver cues for myosin activity, leading to local contraction, while growing MTs deliver cues for actin polymerization, resulting in local expansion. Instead of modeling the actin network explicitly, we represent local contraction and expansion through their effective action on the individual beads that represent the cell boundary. First, actin-MT crosstalk due to shrinking MTs leads to myosin-generated stress in the actin cortex, causing membrane retraction via the RhoA GEF LFc signaling pathway (18). We model this event chain by introducing an effective inward force on a membrane bead that is close to the tip of a shrinking MT. The net inward force on a membrane bead is proportional to the number of shrinking MT tips in the vicinity of the membrane bead (see Fig. **1**). Second, actin-MT crosstalk due to growing MTs leads to the formation of membrane protrusions or lamellipodia due to actin polymerization against a membrane. This induces a retrograde actin flow toward the nucleus, which is opposed by focal adhesions connecting actin filaments across the membrane to the substrate. To describe this sequence of events, we examine a model of lamellipodial protrusion guiding confined cell migration (23). In this model, the local actin polymerization force acting on a segment of the cell membrane is linked to both the actin polymerization rate and the average orientation of actin filaments near the membrane (23–26). Consequently, the actin polymerization force generates an outward-directed velocity component on the membrane beads, which is influenced by the polymerization signals transmitted from the growing microtubule (MT) tips to the actin cortex (c.f. Fig. **1**). To incorporate actin-MT crosstalk, we assume that the outward velocity imparted to the membrane bead along the outward normal is proportional to the number of growing MT tips near the membrane bead. Additionally, myosin-generated contraction forces, mediated by actin filaments between the protrusion's leading edge and the nucleus, counteract the expansion of the protrusion, resulting in an effective elastic coupling between the position of the nucleus and the protrusion edge (23). We model this elastic coupling by linking the beads of the nucleus boundary to those of the cell boundary with elastic springs, as illustrated in Fig. **1**.

Membrane dynamics

The cell and nuclear membrane are modeled as a closed chain of N_{mem} bead spring units (see Fig. **1 A**) (27, 28). Each bead is connected to two neighboring beads on either side via a spring, and an angular potential is considered between the angle formed by every three adjacent beads. The nuclear membrane beads also have a Lennard-Jones interaction with the cell membrane beads within a cut-off distance such that the interaction forces are always repulsive.

$$U^m_{sp} = \frac{1}{2} k_{mem} (l^m - l^m_0)^2 \tag{1}$$

$$U^m_{\theta} = \frac{1}{2} k^m_{\theta} (1 - \cos(\theta - \theta_0)) \tag{2}$$

$$U_{m-n} = 4\epsilon_m \left[\left(\frac{\sigma_m}{r_{m-n}} \right)^{12} - \left(\frac{\sigma_m}{r_{m-n}} \right)^{6} \right] \tag{3}$$

Here, l^m denotes the stretched length of the spring joining two adjacent membrane beads, l^m_0 denotes the rest length of the spring joining two adjacent membrane beads, and r_{m-n} denotes the distance between a membrane bead and nuclear membrane bead. The equation of motion for a cell or nuclear membrane bead then follows as,

$$\dot{\vec{r}}^{c/n}_i = \frac{\vec{F}_{pot}}{\zeta_{c/n}} + \sqrt{\frac{2k_bT}{\zeta_{c/n}}} \eta(t) \tag{4}$$

$\vec{F}_{pot}$ represents the sum total of all forces on the beads due to the potentials considered in Eq. 1-3. $\zeta_{c/n}$ is the coefficient of viscosity of the membrane/nucleus beads and $\eta(t)$ is a Gaussian noise with zero mean and $\langle \eta(t)\eta(t') \rangle = \delta(t - t')$.

MT dynamics

MTs are modeled as open-ended bead spring units with their minus ends anchored at the centrosome (14). The MT beads are connected to adjacent beads on either side via a spring, and an angular potential is considered between the angle formed between three adjacent beads in an MT. The dynamic instability of the MT is incorporated into the model by adding (or removing) beads at the plus end of a growing (or shrinking) MT at intervals of t_{mt} timesteps (see Fig. **1 B**). New beads are added to growing MTs in a given time step only if the new bead falls within the cell and outside the nucleus. The dynamic instability of MTs is governed by four parameters, namely, growth velocity (v_g), shrink velocity (v_s), catastrophe frequency (f_c), and rescue frequency (f_r) (10, 29). An increase in catastrophe frequency (f_c), or a decrease in rescue frequency (f_r), reduces the average MT length. Similarly, an increase in rescue frequency (f_r) or a decrease in catastrophe frequency (f_c) increases the average MT length in the cell.
The membrane beads and MT beads have Lennard-Jones interaction between them within a cut-off distance such that the interaction forces are always repulsive.

$$U_{sp}^{mt} = \frac{1}{2} k_{mt} (l^{mt} - l_0^{mt})^2 \tag{5}$$

$$U_{\theta}^{mt} = \frac{1}{2} k_{\theta}^{mt} (1 - \cos(\theta - \theta_0)) \tag{6}$$

$$U_{mt-m/n} = 4\epsilon_{mt} \left[\left(\frac{\sigma_{mt}}{r_{mt-m/n}} \right)^{12} - \left(\frac{\sigma_{mt}}{r_{mt-m/n}} \right)^{6} \right] \tag{7}$$

Here, l^{mt} denotes the stretched length of the spring joining two adjacent MT beads, l_0^{mt} denotes the rest length of the spring joining two adjacent MT beads, $r_{mt-m/n}$ denotes the distance between a MT bead and a membrane bead (or a nuclear membrane bead). The equation of motion for an MT bead reads,

$$\dot{\vec{r}}_i^{mt} = \frac{\vec{F}_{pot}^{mt}}{\zeta_{mt}} + \sqrt{\frac{2k_bT}{\zeta_{mt}}}\eta(t) \tag{8}$$

The parameters chosen for microtubule dynamics were such that microtubule polymerization did not lead to the formation of stable membrane protrusions.

Dynein dynamics

Dynein motors are placed randomly on membrane beads (or nuclear membrane beads) and scanned for MT beads near them. Upon encountering MT beads within a cut-off distance d_{cut}, dynein motors form spring-like bonds with them. After each time step, the dynein motors can shift to the adjoining MT bead toward the negative end of the MT if the adjoining bead is within the cutoff radius d_{cut}. The dynein walking speed on the MTs is given by v_{dyn}. Dynein pulling forces are modeled as simple spring forces between the membrane (or nuclear membrane) beads and MT beads.

$$U_d = \frac{1}{2} k_d d^2 \tag{9}$$

Where d is the distance between the centers of the membrane (or nuclear membrane) bead and the MT bead.

Actin dynamics

We modeled the effects of actin network dynamics as protrusive and contractile forces on the membrane beads. The protrusive forces caused by actin polymerization are considered to add a component to the velocity of the membrane beads directed in the outward normal direction (23). This outward component of the velocity depends on the average local orientation S_{act} of the actin filaments in the vicinity of the membrane bead and the local actin polymerization rate r_p. The net outward velocity added to the membrane beads is then $l_a S_{act} r_p$, where l_a is the diameter of individual actin filaments. Mechanotransduction of forces from the cell membrane to the nuclear membrane is known to happen due to the interconnection of actin filaments and intermediate filaments with the nuclear membrane (30, 31). Myosin motors walk on counter-oriented actin filaments in the crossover region between the cell membrane and the nuclear membrane to generate a contraction force (32). An increase in actin polymerization activity at the cell front increases the retrograde flow of actin. This results in more active myosin motors in the crossover region between the protrusion tip and the nucleus, leading to an increase in contraction forces. The strength of this

contractile force depends on the actin retrograde flow velocity v_i^r at the protruding membrane bead. To evaluate the net velocity change of the protruding membrane beads due to cortical actin activity, we follow (23), and equate the net change in bead velocity along the outward direction to the difference between the outward actin polymerization velocity and inward retrograde flow velocity.

$$\dot{r}_i^c = l_a S_{act} r_p - v_i^r \tag{10}$$

The retrograde flow velocity can be assumed to be driven by the membrane tension f_τ and the contraction force f_c at the membrane due to coupling with the nucleus through actin filaments and myosin motors. It can be shown that f_c and f_τ vary linearly with the length of the protrusion (23, 25, 26, 33).

$$\vec{v}_i^r = -\frac{k_c}{\zeta_c}(\vec{r}_i^c - \vec{r}_i^n) \tag{11}$$

where k_c represents the strength of the uniform linear coupling between the cell membrane beads and nuclear membrane beads. The protrusion length is the distance $(\vec{r}_i^c - \vec{r}_i^n)$ between the cell membrane bead and the nuclear membrane bead. The cell membrane attaches to the substrate through the formation of focal adhesions. The formation and dissolution of focal adhesions modulate the interaction between the membrane. This interaction is included in our model as an effective viscous drag on the membrane beads whose strength is given by the coefficient ζ_c (23, 34). We also consider a local contractile force F_c due to myosin activity in the cell cortex along the cell membrane that does not couple with the nucleus, directed opposite to the outward normal at the membrane. The final equation of motion for the membrane beads then reads as follows:

$$\dot{\vec{r}}_i^c = -\frac{k_c}{\zeta_c}(\vec{r}_i^c - \vec{r}_i^n) + l_p S_{act} r_p \hat{n}_i - \frac{F_c}{\zeta_c}\hat{n}_i + \frac{\vec{F_{pot}}}{\zeta_c} + \sqrt{\frac{2k_bT}{\zeta_c}}\eta(t) \tag{12}$$

$\hat{n}_i$ is a unit vector pointing along the outward normal along the membrane bead. F_{pot} is the sum of all the forces due to the interaction potentials between the membrane and the nucleus and MT beads. We assume that the values of S_{act} and r_p depend on actin polymerization cues that are supplied to the region near the membrane beads by polymerizing MTs and their values are calculated according to,

$$l_p S_{act} r_p = l_p S_0 r_0 + C_p n_{mt}^{pol} \tag{13}$$

Where $l_p S_0 r_0$ is the component of the outward polarization velocity in the absence of polarization cues delivered by the growing MT tip, n_{mt}^{pol} is the number of polymerizing MTs in the vicinity of the membrane bead and C_p is the component of protrusion velocity added per polymerizing MT. The strength of the contraction force F_c, due to myosin activity at the cortex, is considered to depend on contraction cues provided by depolymerizing MT tips near the membrane beads and varies according to :

$$F_c = F_0 + C_d n_{mt}^d \tag{14}$$

Where F_0 is the strength of the contraction force in the absence of any MT-supplied contraction cue. C_d is the net contraction force added per depolymerizing MT present near the membrane bead, and n_{mt}^d is the number of depolymerizing MTs near the membrane bead. The distance of the membrane bead from a MT tip, within which the dynamics of actin polymerization or acto-myosin contraction at the cortex can be influenced by the MT tip delivered signal, was chosen as $2 \times 1.12\sigma_{mt}$. Finally, the equation of motion of the nucleus beads reads,

$$\dot{\vec{r}}_i^n = -\frac{k_c}{\zeta_n}(\vec{r}_i^n - \vec{r}_i^c) + \frac{F_{pot}^n}{\zeta_n} + \sqrt{\frac{2k_bT}{\zeta_n}}\eta(t) \tag{15}$$

Where F_{pot}^n represents the sum of all the forces on the nucleus bead due to the steric and dynein interactions considered in our model.

SIMULATION METHOD

All simulations were initialized with a circular cell and nucleus and the centrosome was placed randomly within the cell. All MTs were initialized to be growing and their directions of growth were chosen randomly. The simulation time step was taken to be $0.1 sec$. The beads were added or removed from the MT tips after each N_{pol} time step according to their growth or shrinkage state. The probabilities for MT catastrophe or rescue were calculated after every N_{pol} time steps and the MT state was updated

accordingly. N_{pol} was taken as 10 timesteps. The number of growing or shrinking MT tips near a membrane bead was checked at every time step and actin polymerization or cortical contraction signal strengths were calculated. Dynein motors were placed on the cell membrane and nuclear membrane beads randomly, and positions of attachments were searched on nearby MTs at every time step. Upon finding possible sites of attachments, dynein motor bonds were established between the membrane and MT beads with a certain probability P_{dyn}. Established dynein bonds were checked for turnover and bonds were detached according to the turnover probability P_{turn} at every time step. For the force dynamics, we considered a relaxation timestep of $0.001 sec$. Forces on every component in the model were calculated at every relaxation time step and their positions were updated accordingly.

The codes were developed in C using OpenMP. The data analysis and plots were done in Python and Gnuplot. The computational time for a single simulation running on 20 processors (Intel Xeon CPU, having a clock speed of 2 GHz and RAM 64 GB) was in the range of 10-20 hours.

DATA ANALYSIS

Persistence length

The local directional persistence, or the ability of the cell to maintain its direction of motion, is quantified as $p = cos\theta$ with θ being the angle between the instantaneous velocity directions at two time steps. The persistence length l_p can then be calculated from $p = e^{-\frac{d}{l_p}}$, where d is the displacement of the cell between the two time steps. The local persistence length l_p was evaluated at consecutive time steps throughout the motion of the cell and its average was calculated as the mean persistence length $\langle l_p \rangle$ (35).

Local mean square displacement and velocity standard deviation analysis of cell trajectories

Local mean square displacements were evaluated by considering a rolling time window of $N_t = 30$ points at consecutive time steps 300 seconds apart. At every time step t_i, the local mean square displacement ΔR_i^2 was evaluated as a function of the time lag $\tau_m = m\delta t$ as (36),

$$\Delta R_i^2(t_i, \tau_m) = \frac{1}{N_t - m + 1} \sum_{j=-\frac{N_t}{2}}^{j=\frac{N_t}{2}-m} \Big[\mathbf{R}(t_{i+j} + m\delta t) - \mathbf{R}(t_{i+j})\Big]^2 \tag{16}$$

where δt is the time between two rolling window points and $\mathbf{R}_i = (X_i, Y_i)$ are the coordinates of the cell centroid. The total duration of the rolling window is $T = N_t \delta t$.

The standard deviation of the velocity was calculated at consecutive time steps from the values of the velocity direction $\phi_i(t_i)$ as (36),

$$\Delta\phi(t_i) = \left[\frac{1}{N_t - m + 1} \sum_{j=-\frac{N_t}{2}}^{j=\frac{N_t}{2}-m} (\phi(t_{i+j} + m\delta t) - \phi(t_{i+j}))^2\right]^{\frac{1}{2}} \tag{17}$$

the value of m was chosen such that $m\delta t = T/4$.

The local mean square displacement ΔR_i^2 was assumed to scale with the time lag τ_m as,

$$\Delta R_i^2 = A\tau_m^\alpha \tag{18}$$

For $\alpha = 1$, the motion of the cell is completely diffusive, and for $\alpha = 2$, the motion of the cell is ballistic. We considered the motion of the cell in two separate phases. For $\alpha > 1.7$ and $\Delta\phi < 0.9$, the cell migration was considered to be in the directed motion phase; otherwise, the cell motion was considered to be in the random migration phase.

Evaluation of cell polarization by MT signals

To evaluate the distribution of MT tips along the membrane in Fig. **S2 B**, we counted the number of MT tips associated with membrane beads. Average MT numbers along the cell membrane were computed by identifying MT tips located near a

membrane bead, such that the bead lies within the range where polymerization and contraction signals can be transmitted. A rolling average was then applied over 60 consecutive snapshots spanning a 60-second interval. The data were plotted based on the angular position of each membrane bead relative to the direction of migration.

SUPPORTING REFERENCES

1. Lee, S. Y., P. W. A. Schönhöfer, and S. C. Glotzer, 2023. Complex motion of steerable vesicular robots filled with active colloidal rods. *Scientific Reports* 13:22773.

2. Koonce, M. P., M. P. Koonce, A. Khodjakov, and A. Khodjakov, 2002. Dynamic microtubules in Dictyostelium. *Journal of Muscle Research and Cell Motility* 23:613–619.

3. Ryder, M. I., R. N. Weinreb, and R. Niederman, 1988. Microtubule-granule relationships in motile human polymorphonuclear leukocytes. *Anatomical Record-advances in Integrative Anatomy and Evolutionary Biology* 221:679–686.

4. Schliwa, M., K. B. Pryzwansky, and U. Euteneuer, 1982. Centrosome splitting in neutrophils: an unusual phenomenon related to cell activation and motility. *Cell* 31:705–717.

5. Schulze, E., and M. Kirschner, 1986. Microtubule dynamics in interphase cells. *The Journal of cell biology* 102:1020–1031.

6. Murphy, P., 1976. Morphology and Cellular Physiology of Neutrophil Granulocytes, Springer US, Boston, MA, 17–31.

7. Lammerding, J., 2011. Mechanics of the Nucleus, John Wiley & Sons, Ltd, 783–807.

8. van Haren, J., and T. Wittmann, 2019. Microtubule Plus End Dynamics "-" Do We Know How Microtubules Grow? *BioEssays* 41:1800194.

9. Zwetsloot, A. J., G. Tut, and A. Straube, 2018. Measuring microtubule dynamics. *Essays in Biochemistry* 62:725–735.

10. Kirschner, M., and T. Mitchison, 1986. Beyond self-assembly: From microtubules to morphogenesis. *Cell* 45:329–342.

11. Walker, R. A., E. T. O'Brien, N. K. Pryer, M. F. Soboeiro, W. A. Voter, H. P. Erickson, and E. D. Salmon, 1988. Dynamic instability of individual microtubules analyzed by video light microscopy: rate constants and transition frequencies. *Journal of Cell Biology* 107:1437–1448.

12. Vutukuri, H. R., M. Hoore, C. Abaurrea-Velasco, L. van Buren, A. Dutto, T. Auth, D. A. Fedosov, G. Gompper, and J. Vermant, 2020. Active particles induce large shape deformations in giant lipid vesicles. *Nature* 586:52–56.

13. Wang, C., Y.-k. Guo, W.-d. Tian, and K. Chen, 2019. Shape transformation and manipulation of a vesicle by active particles. *The Journal of Chemical Physics* 150:044907.

14. Soheilypour, M., M. Peyro, S. Peter, and M. Mofrad, 2015. Buckling Behavior of Individual and Bundled Microtubules. *Biophysical Journal* 108:1718–1726.

15. Oelz, D. B., U. del Castillo, V. I. Gelfand, and A. Mogilner, 2018. Microtubule Dynamics, Kinesin-1 Sliding, and Dynein Action Drive Growth of Cell Processes. *Biophysical Journal* 115:1614–1624.

16. Ezber, Y., V. Belyy, S. Can, and A. Yildiz, 2020. Dynein harnesses active fluctuations of microtubules for faster movement. *Nature Physics* 16:312–316.

17. Liao, G., T. Nagasaki, and G. G. Gundersen, 1995. Low concentrations of nocodazole interfere with fibroblast locomotion without significantly affecting microtubule level: implications for the role of dynamic microtubules in cell locomotion. *Journal of Cell Science* 108:3473–3483.

18. Kopf, A., J. Renkawitz, R. Hauschild, I. Girkontaite, K. Tedford, J. Merrin, O. Thorn-Seshold, D. Trauner, H. Häcker, K. D. Fischer, E. Kiermaier, and M. Sixt, 2020. Microtubules control cellular shape and coherence in amoeboid migrating cells. *Journal of Cell Biology* 219:e201907154.

19. Dogterom, M., and G. H. Koenderink, 2019. Actin–microtubule crosstalk in cell biology. *Nature Reviews Molecular Cell Biology* 20:38–54.

20. Henty-Ridilla, J. L., A. Rankova, J. A. Eskin, K. Kenny, and B. L. Goode, 2016. Accelerated actin filament polymerization from microtubule plus ends. *Science* 352:1004–1009.

21. Hornak, I., and H. Rieger, 2020. Stochastic Model of T Cell Repolarization during Target Elimination (I). *Biophysical Journal* 118:1733–1748.

22. Hornak, I., and H. Rieger, 2022. Stochastic model of T cell repolarization during target elimination (II). *Biophysical Journal* 121:1246–1265.

23. Flommersfeld, J., S. Stöberl, O. Shah, J. O. Rädler, and C. P. Broedersz, 2024. Geometry-Sensitive Protrusion Growth Directs Confined Cell Migration. *Phys. Rev. Lett.* 132:098401.

24. Lavi, I., M. Piel, A.-M. Lennon-Duménil, R. Voituriez, and N. S. Gov, 2016. Deterministic patterns in cell motility. *Nature Physics* 12:1146–1152.

25. Sens, P., 2020. Stick–slip model for actin-driven cell protrusions, cell polarization, and crawling. *Proceedings of the National Academy of Sciences* 117:24670–24678.

26. Ron, J. E., P. Monzo, N. C. Gauthier, R. Voituriez, and N. S. Gov, 2020. One-dimensional cell motility patterns. *Phys. Rev. Res.* 2:033237.

27. Abaurrea-Velasco, C., T. Auth, and G. Gompper, 2019. Vesicles with internal active filaments: self-organized propulsion controls shape, motility, and dynamical response. *New Journal of Physics* 21:123024.

28. Wang, H., Z. Jia, and Y. Fang, 2024. Chemo-mechanical model of cell polarization initiated by structural polarity. *Soft Matter* 20:8407–8419.

29. Dogterom, M., and S. Leibler, 1993. Physical Aspects of the Growth and Regulation of Microtubule Structures. *Physical Review Letters* 70(9):1347–1350.

30. Stöberl, S., J. Flommersfeld, M. M. Kreft, M. Benoit, C. P. Broedersz, and J. O. Rädler, 2024. Nuclear deformation and dynamics of migrating cells in 3D confinement reveal adaptation of pulling and pushing forces. *Science advances* 10:eadm9195.

31. Maniotis, A. J., C. S. Chen, and D. E. Ingber, 1997. Demonstration of mechanical connections between integrins, cytoskeletal filaments, and nucleoplasm that stabilize nuclear structure. *Proceedings of the National Academy of Sciences* 94:849–854.

32. Lou, S. S., A. Diz-Muñoz, O. D. Weiner, D. A. Fletcher, and J. A. Theriot, 2015. Myosin light chain kinase regulates cell polarization independently of membrane tension or Rho kinase. *Journal of Cell Biology* 209:275–288.

33. Brückner, D. B., M. Schmitt, A. Fink, G. Ladurner, J. Flommersfeld, N. Arlt, E. Hannezo, J. O. Rädler, and C. P. Broedersz, 2022. Geometry Adaptation of Protrusion and Polarity Dynamics in Confined Cell Migration. *Phys. Rev. X* 12:031041.

34. Kim, D.-H., and D. Wirtz, 2013. Focal adhesion size uniquely predicts cell migration. *The FASEB Journal* 27:1351–1361.

35. Shaebani, M. R., R. Jose, L. Santen, L. Stankevicins, and F. Lautenschläger, 2020. Persistence-Speed Coupling Enhances the Search Efficiency of Migrating Immune Cells. *Phys. Rev. Lett.* 125:268102.

36. Arcizet, D., S. Capito, M. Gorelashvili, C. Leonhardt, M. Vollmer, S. Youssef, S. Rappl, and D. Heinrich, 2012. Contact-controlled amoeboid motility induces dynamic cell trapping in 3D-microstructured surfaces. *Soft Matter* 8:1473–1481.
