# Supplementary material for: Modeling actin-microtubule crosstalk in migrating cells
Source: Biophys J. 2025 Sep 23;124(21):3742–54. doi: 10.1016/j.bpj.2025.09.029 (PMC12709440; doi:10.1016/j.bpj.2025.09.029)
Supplement: Document S2. Article plus supporting material [file mmc7.pdf]

Biophysical Journal
Article

Modeling actin-microtubule crosstalk in migrating cells

Pinaki Nayak,[1] Anil Kumar Dasanna,[2] Raja Paul,[1] and Heiko Rieger[3,*]
[1]School of Mathematical and Computational Sciences, Indian Association for the Cultivation of Science, Kolkata, India; [2]Department of Physical Sciences, Indian Institute of Science Education and Research Mohali, Knowledge City, Manauli, India; and [3]Center for Biophysics & Department for Theoretical Physics, Saarland University, Saarbrücken, Germany

ABSTRACT Actin-microtubule crosstalk regulates the polarity and morphology of migrating cells and encompasses mechanical interactions, mediated by cross-linkers, molecular motors, and cytoskeletal regulators. Recent experiments indicate that local microtubule depolymerization promotes local actomyosin retraction, whereas local microtubule polymerization promotes local actin polymerization. Based on these observations, we develop a computational whole-cell model involving dynamic microtubules interacting mechanically and chemically with an active cell boundary. Specifically, the tips of microtubules send signals for local expansion or contraction to the active cell boundary, depending on whether they are in the growth or shrink phase. A rich, self-organized, dynamic behavior emerges, characterized by the repositioning of the microtubule-organizing center relative to the nucleus and the direction of migration. This also includes a variety of migration patterns, cell morphologies, and complex responses to obstacles in microfluidic and obstacle park environments. We demonstrate that microtubule length and numbers have a significant impact on these features, highlighting the need for new experimental investigations. Thus, the model provides a unified framework that explains a wide range of experimental observations and setups where actin-microtubule crosstalk plays a crucial role.

SIGNIFICANCE The interplay between microtubule dynamics, centrosome positioning, cell polarization, and migration is fascinating and enigmatic. Recent experiments have demonstrated a strong correlation between microtubule growth and shrinkage and actin protrusion and actomyosin contraction, mediated by actin-microtubule crosstalk. In this study, we introduce, for the first time to our knowledge, a quantitative model of cell migration that accounts for actin-microtubule crosstalk and uncovers a rich, self-organized dynamic behavior, including cell polarization, centrosome positioning, migration patterns, cell morphologies, and intricate responses in microfluidic environments and synthetic obstacle arrays. Thus, our work represents a significant advancement in the modeling and understanding of cell migration in complex environments and provides a powerful method to incorporate actomyosin dynamics into computational models.

INTRODUCTION

Cell migration is primarily driven by forces generated at the actin cortex underlying the cell membrane. The onset of migration requires the cell to be polarized by forming a protruding front edge and a contracting rear edge (1). Protrusions at the front originate from the increased actin polymerization supported by the focal adhesions formed in contact with the extracellular matrix (2–4). Membrane retraction at the rear is realized inside a cell by contractile forces arising from myosin activity and dissolution of focal adhesions (2,5,6). The formation of protrusion or membrane retraction is guided through the reorganization of the cytoskeleton by the delivery of molecular regulatory signals. Microtubules (MTs) are known to play an important role in the distribution of these regulatory signals leading to cell polarization during migration (7–9). The tips of growing MTs reach the protruding front edge of the cell to deliver actin polymerization signals that stabilize the protrusions (6,10,11). MT depolymerization induces the activation of RhoA, which increases myosin-II activity, increasing contractility and cell membrane retraction (12–14). Differential stability of MTs at the front and rear edge thus leads to symmetry breaking and polarization of the cell (6,15). MTs have also been suggested to play a critical role in the modulation of cell shape and stabilization or retraction of

Submitted April 29, 2025, and accepted for publication September 18, 2025.
*Correspondence: h.rieger@mx.uni-saarland.de
Editor: Timo Betz.
https://doi.org/10.1016/j.bpj.2025.09.029
© 2025 The Author(s). Published by Elsevier Inc. on behalf of Biophysical Society.
This is an open access article under the CC BY license (http://creativecommons.org/licenses/by/4.0/).

protrusions when the cell is navigating through obstacles, thereby dictating the cell migration path (6).

Centrosomes, being the primary MT organizing centers (MTOCs) in animal cells, can guide the choice of the front and rear edge of the cell, via their preferential position with respect to the nucleus. The centrosomal position, anterior or posterior to the nucleus, has been investigated in various cells, proposing mechanisms that may guide this choice. Cells undergoing mesenchymal migration are characterized by the formation of nascent focal adhesions with the extracellular matrix at the leading edge and the rupture of aged focal adhesions at the rear edge (16–18). The centrosome is placed ahead of the nucleus more often in cells migrating through stiff extracellular matrices (19). Fast-moving ameboid cells, characterized by reduced focal adhesions, are typically known to position their centrosome posterior to the nucleus during migration (20). Interestingly, recent experimental studies suggest that leukocytes can alternate between a centrosome-forward or nucleus-forward configuration while navigating a congested microenvironment (21). Cells may alter the position of the centrosome to modify the distribution of MTs, which in turn helps coordinate the polymerization and contraction signals in the actin cortex that drive cell movement. As MTs grow, they can extend toward the cell membrane or nucleus and undergo buckling. This buckling generates a pushing force that pushes the centrosome away from the point of contact with either the cell or nuclear membrane (22,23). MTs can also slide along the cell membrane or nucleus (24). Dynein motors present at the cortex or nuclear membrane attach to the sliding MTs and walk toward their minus end, effectively pulling the MTs and the centrosome (25,26). The position of the centrosome within a moving cell results from a complex balance of forces, which are generated through the interactions of the MTs with the centrosome, along with membrane remodeling driven by polarity signals from the MTs to the cortex. This raises several questions: how does the centrosome position itself anterior or posterior to the nucleus during migration? How do changes in MT dynamics influence the centrosome's position? And, what impact do these alterations in the MT network have on actin-MT interactions and the cell's overall migration?

Phenomenological and mechanistic acto-myosin models of cell migration have been the subject of extensive study (27–32). Models that focus on the microscopic details of actin polymerization and myosin motor activity are computationally intensive and challenging to generalize for studying cell migration in both two (2D) and three dimensions (3D) (33,34). In contrast, phenomenological models of cell migration often focus on cortical activity and membrane-substrate interactions but tend to neglect the contribution of MT-driven chemical signaling, which plays a key role in regulating cortical dynamics and initiating symmetry breaking (29,35–38).

Although it is well established that MT-actin crosstalk plays a crucial role in cell migration and other cellular functions (39), a mechanistic whole-cell model integrating MT dynamics and actin-generated forces to explore the self-organization of centrosome positioning, cellular shape changes, and migratory behavior is still lacking. In this work, we therefore introduce a phenomenological model based on the experimental observations reported in Refs. (6,7,10,13,15), which correlate MT growth and shrinkage with actin polymerization, depolymerization, and contraction. We examine how the position of the centrosome and the direction of migration are influenced by MT dynamics. Additionally, we explore how cells might leverage MT-actin crosstalk to switch between persistent migration and diffusive movement. Finally, we investigate how the positioning of the centrosome, either anterior or posterior to the nucleus, may guide the cell's path when navigating narrow channels.

MATERIAL AND METHODS

Computational model

We develop a mechanistic model of cell migration based on membrane protrusion and retraction coupled to the actomyosin cortex and cytoskeleton. Key processes include actin polymerization, myosin-driven contraction, actin polarization, focal adhesion dynamics, and membrane tension. MT depolymerization regulates actomyosin contraction via Rho GTPase signaling (6), whereas MT polymerization promotes actin-driven protrusions by transporting signaling molecules to the leading edge (6,10,39). These two effects form the basis of our whole-cell MT-actin crosstalk model during migration.

We model 2D mesenchymal migration using bead-spring loops to represent the semiflexible boundaries of the cell and nucleus, and bead-spring polymers for dynamic MTs that grow or shrink at their plus ends. MTs are anchored at the MTOC, assumed to coincide with the centrosome. Growing MTs apply pushing forces when contacting the membrane, whereas dyneins anchored at the cell and nuclear membranes exert pulling forces on MTs (40,41). A schematic of these model components is shown in Fig. 1*A* and *B*, with full mathematical details in the supporting material.

MT-actin crosstalk is incorporated through regulatory signals transmitted by MT tips. Shrinking MTs trigger local myosin activation, causing membrane contraction, whereas growing MTs promote actin polymerization, driving membrane protrusion. Rather than modeling the actin network explicitly, we represent their effects through effective forces on membrane beads: shrinking MT tips apply inward forces proportional to their local density (6), and growing tips induce outward velocities proportional to the number of tips near a membrane segment (29,37,42). In addition, contractile forces generated by myosin between the nucleus and protrusion edge create an elastic coupling (38,43–45), which we model by connecting nucleus and membrane beads with springs (see Fig. 1 *C–E* and details in the supporting material).

RESULTS AND DISCUSSION

Migrating cell centrosome can lead or trail nucleus depending upon MT length

First, we examine how centrosome positioning and migration characteristics in our model are influenced by MT properties, such as the average MT length. The trajectories of the cell centroid indicate that for average MT lengths of

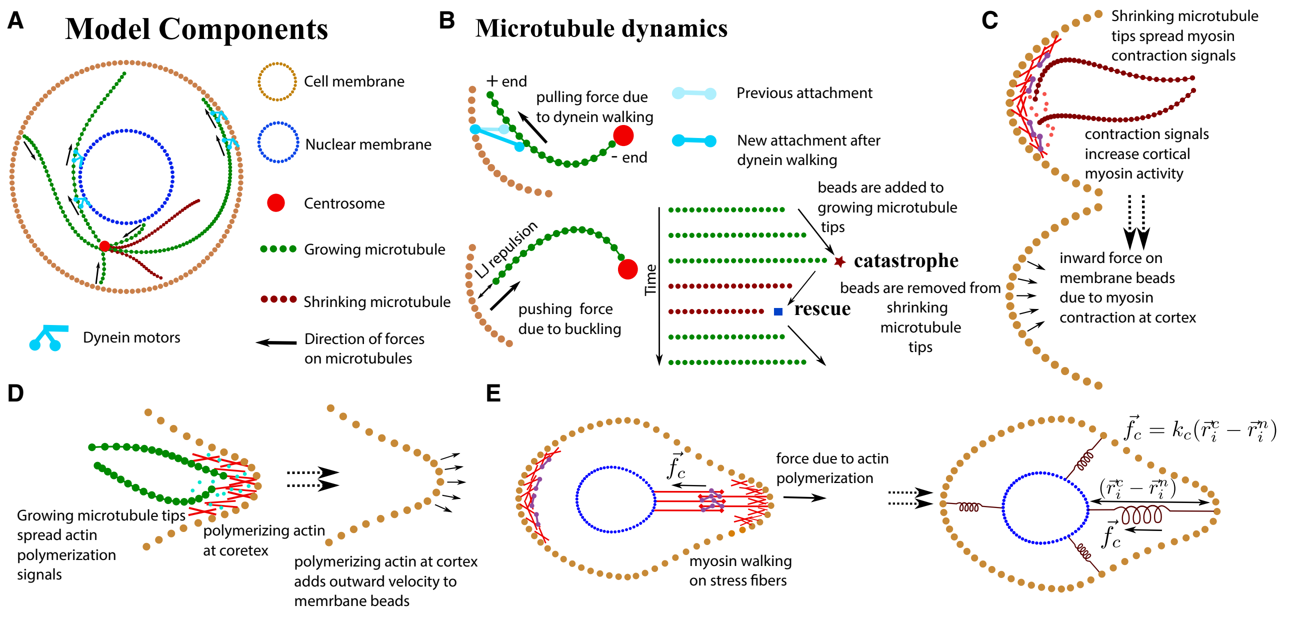

FIGURE 1 Schematic of the model. (*A*) Sketch of a migrating cell, showing membrane, nucleus, centrosome, MTs, actin, and dynein motors. (*B*) MTs grow by adding beads at the plus end and shrink by bead removal. Growing MTs can undergo catastrophe and shrink, whereas shrinking MTs can be rescued. Dynein, anchored to the cell and nuclear membranes, attaches to MTs and moves toward the minus end, exerting pulling forces on the centrosome. Steric interactions modeled as Lennard-Jones repulsive forces between MT beads and the membrane cause MT buckling and generate pushing forces. (*C*) Shrinking MTs transmit myosin contraction signals to the actin cortex, producing inward-directed forces on membrane beads. (*D*) Growing MT tips deliver actin polymerization signals, driving outward membrane bead displacement. (*E*) Myosin contractility across overlapping actin filaments couples the nuclear and cell membranes through a linear elastic force that resists membrane protrusion.

$l_{mt} = 9.3 - 16\ \mu m$, the cell exhibited directed migration, whereas for an average MT length of $l_{mt} = 3\ \mu m$ the cellular trajectories showed random motion (Fig. 2 *A*; see Fig. S1 and Videos S1 and S2). We then focused on the MT lengths that resulted in the directed migration of the cell (see Fig. 2 *B* and *C*). Our results showed that for an average MT length of $l_{mt} = 9.3\ \mu m$ (which is two-thirds of the cell diameter, henceforth referred to as "regular MT"), the centrosome was predominantly positioned ahead of the nucleus (Fig. 2 *B* and *D* (blue line)). When the average MT length was increased to $l_{mt} = 16\ \mu m$ (greater than the cell diameter, henceforth referred to as "long MT"), the centrosome preferentially remained behind the nucleus in the direction of migration (Fig. 2 *C* and *D* (red line)). To understand this preferential positioning of the centrosome in both cases, we examined the MT dynamics. MTs radiated from the centrosome in all directions, extending to both the cell and nuclear membranes. However, the presence of the nucleus obstructed the MTs from reaching the portion of the cell membrane behind it. Regular MTs were not long enough to slide along the nuclear membrane and extend to the rear portion of the cell membrane (Fig. 2 *B*). The growing MTs that reach the cell membrane have most of their length within the cytoplasm, with only a short segment ($\sim 1.8\ \mu$m per MT) near the MT tips remaining in close contact (distance less than $2 \times 1.12\sigma_{\rm mt}$) with the cell membrane (Fig. 2 *B*). The MT tips deliver actin polymerization signals to the membrane region near their tips. When an MT undergoes a catastrophe, its tip recedes from the membrane. For $l_{mt} = 9.3\ \mu m$, only a few shrinking MT tips are observed near the membrane-cortex region (see Fig. S2 *A* and *B*). Hence, the contraction signals generated by shrinking MT tips play little role in cell polarization. In contrast, a large number of growing MTs are located at the membrane-cortex region closer to the centrosome (see Fig. S2 *A* and *B*). As a result, actin polymerization signals are mainly distributed in these areas with a high density of growing MT tips. Therefore, in this case, the difference in actin polymerization signals between membrane-cortex regions proximal and distal to the centrosome gives rise to cell polarization. As the cell advances, propelled by actin polymerization at the leading edge, the nucleus and rear regions are pulled forward through elastic coupling between the membrane and the nucleus (see Fig. S2 *A* and *B*). This way, asymmetry in the distribution of actin polymerization signals breaks the symmetry of the stationary cell. The regions receiving signals from growing MTs form the protruding front end, whereas areas devoid of MT tips form the rear. As a result, the cell migrates with the centrosome near the cell center and the nucleus positioned behind it toward the rear end of the cell. For regular MTs, we find that the probability distribution of the angle between the cell's direction of motion and the vector from the nucleus to the centrosome peaks near zero (Fig. 2 *D* (blue line)), corroborating the result that the centrosome remains positioned ahead of the nucleus throughout migration.

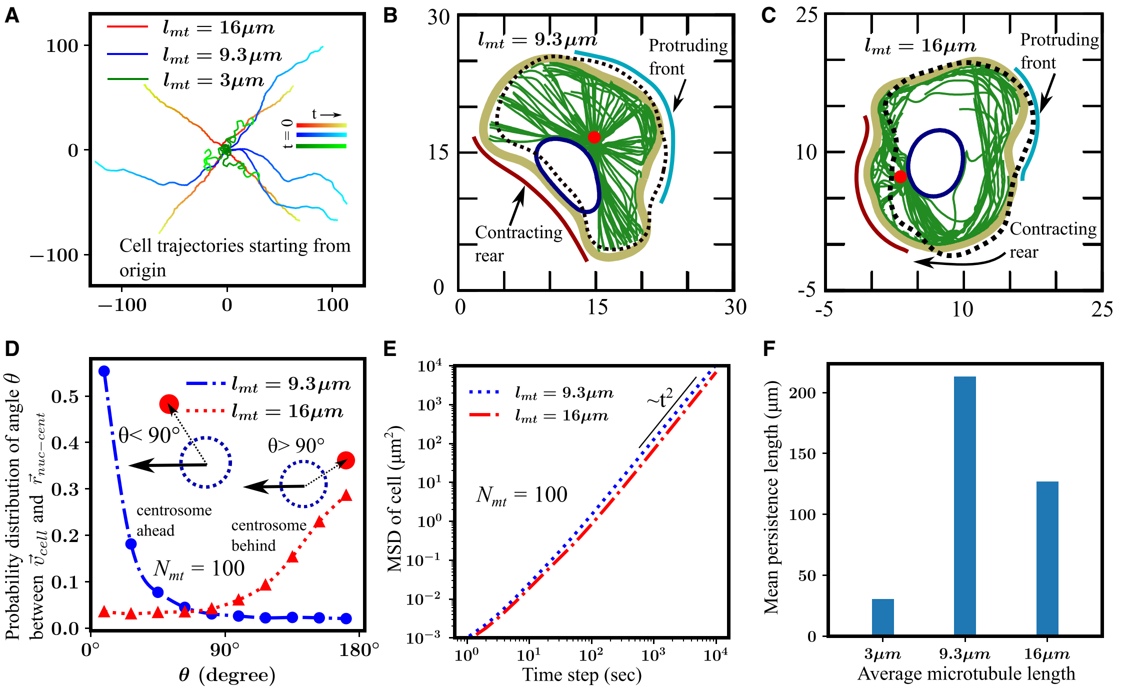

FIGURE 2 Centrosome position depends on MT length. (*A*) Cell trajectories show directed migration for $l_{mt} = 9.3$ μm and 16 μm but random motion for $l_{mt} = 3$ μm. (*B*) Simulations with $l_{mt} = 9.3$ μm show the centrosome anterior to the nucleus; growing MT tips drive front protrusions, and cell rear is pulled via nucleus to membrane elastic coupling. (*C*) For $l_{mt} = 16$ μm, the centrosome shifts posterior; growing MTs promote front protrusions, whereas shrinking MTs trigger rear contraction. (*D*) Probability distribution of the angle between migration direction and centrosome-nucleus axis; $\theta < 90^{\circ}$ indicates centrosome ahead and $\theta > 90^{\circ}$ behind. Regular MTs (9.3 μm) position the centrosome ahead and long MTs (16 μm) behind. (*E*) Mean-square displacement shows t^2 scaling, indicating ballistic migration for $l_{mt} = 9.3$–16 μm. (*F*) Mean persistence length of migrating cells across different MT lengths, showing high persistence for $l_{mt} = 9.3$–16 μm (one-way ANOVA test *p*-value <0.0001).

Cells with long MTs ($l_{mt} = 16\ \mu m$) are polarized with the centrosome positioned posterior to the nucleus. MT tips extending from the centrosome reach either the cell membrane or nuclear membrane and can glide along these membranes to eventually reach the distal end of the cell, behind the nucleus (Fig. 2 *C*). Since the average MT length exceeds the cell diameter, growing MTs tend to glide along the cell or nuclear membrane, with an average of 60 MTs maintaining more than 4 μm of their length in close contact with membrane beads, until they reach the distal end of the cell (Fig. 2 *C*). Growing MTs that encounter the membrane near the centrosome bend and continue toward the distal end. Over time, these MT tips accumulate at the far end of the membrane, where they contribute to actin polymerization signaling. This results in a higher concentration of polymerization cues at the membrane region farthest from the centrosome (see Fig. S2 *C*).

In contrast, long MTs that reach the distal cell membrane may undergo catastrophe. These MTs typically have a substantial segment of their length ($\sim 8\ \mu m$ per MT) in contact with and gliding along the cell membrane (Fig. 2 *C*). When catastrophe occurs, the MTs begin to shrink toward the centrosome, spreading contraction signals along the cortical regions they traverse. As the shrinking tips retract, they move away from the distal membrane and approach the centrosome. Because the chosen dynamic instability parameters ($f_r = 0.04\ s^{-1}$) do not permit frequent rescue events, the contraction signals from shrinking MTs are predominantly concentrated in the cortical region near the centrosome (see Fig. S2 *C* and *D*). This process polarizes the cell, with protrusions forming at the distal membrane (farthest from the centrosome) and retraction occurring at the proximal membrane (closer to the centrosome) due to elevated cortical myosin activity. As a result, the cell migrates with the centrosome positioned posterior to the nucleus. For long MTs, the probability distribution of the angle between the cell's direction of motion and the vector from the nucleus to the centrosome peaks near 180° (Fig. 2 *D* (red line)), suggesting that the centrosome largely remains posterior to the nucleus during cell migration (5,20).

Next, we characterize the persistence of migrating cells for various average MT lengths, which correspond to different centrosome positioning. The mean-square displacement (MSD) of the cell centroid, scaled by the square of time, suggests ballistic motion ($MSD \propto t^2$) (Fig. 2 *E*). This indicates that the cell can move in a ballistic mode regardless of whether the centrosome is positioned anterior or posterior to the nucleus, as long as the cell remains polarized. We also examined how the persistence length of migrating cells changes with different MT lengths. For short MTs ($l_{mt} = 3\ \mu m$), the mean persistence length was small ($\approx 25\ \mu m$), indicating that the cell frequently

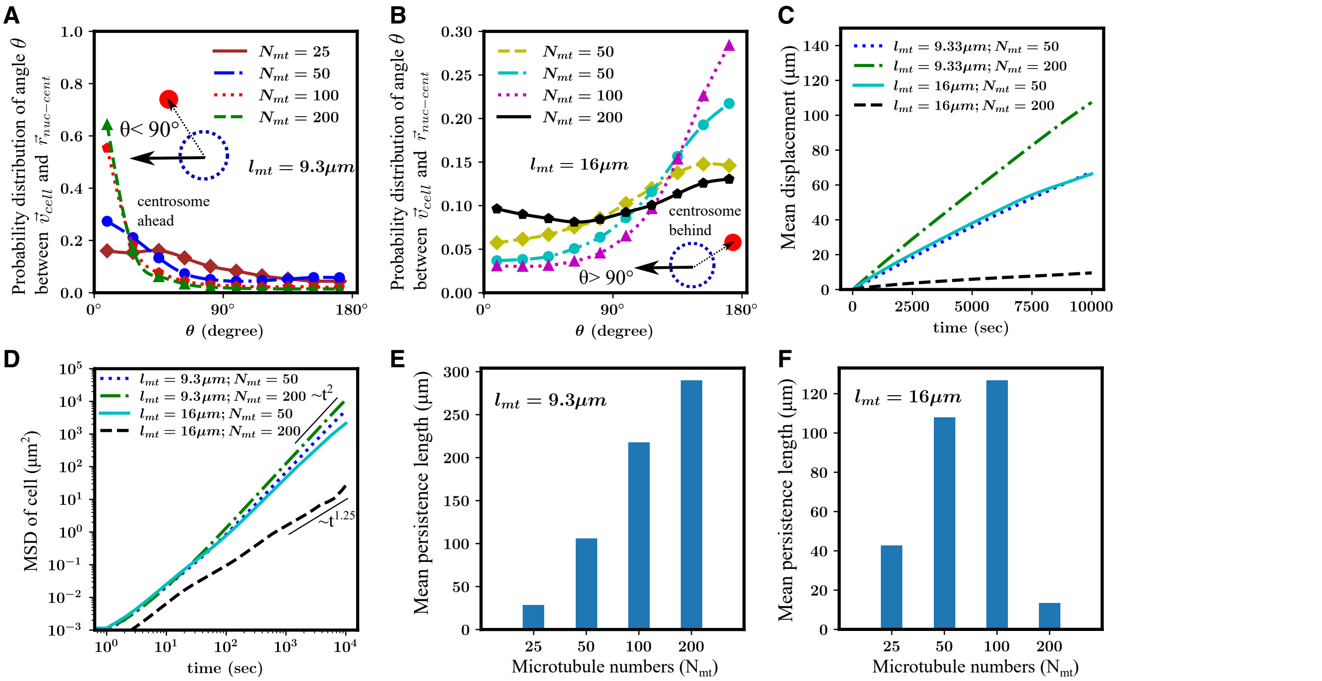

FIGURE 3 MT number affects persistence of cell migration. (*A and B*) Probability distribution of the angle between the direction of motion and the nucleus-to-centrosome vector for $l_{mt} = 9.3\ \mu m$ and $16\ \mu m$, for different MT numbers. (*C*) Mean displacement of the cell centroid as a function of time for varying MT numbers and average lengths. (*D*) Mean-square displacement of the cell over time for varying MT numbers and average lengths. An increase in the number of MTs, with an average MT length of $l_{mt} = 16\ \mu m$, causes the cell to transition from ballistic motion ($MSD \propto t^2$) to super-diffusive motion ($MSD \propto t^{1.25}$). (*E*) Persistence length of migration for various MT numbers with $l_{mt} = 9.3\ \mu m$. Persistence length increases steadily as the MT number increases. (*F*) Persistence length of migration for various MT numbers with $l_{mt} = 16\ \mu m$. Persistence length of the cell initially increases with MT number but decreases when the MT number becomes high.

deviated from its direction of migration over short distances (Fig. 2 *F*). In contrast, the mean persistence length was significantly higher for regular and longer MTs ($l_{mt} = 9.3$ and $16\ \mu m$, respectively), showing that the cell was capable of directed migration with regular and long MTs. Interestingly, the persistence length was greatest for the regular MT length of $l_{mt} = 9.3\ \mu m$, indicating that cells with this MT length exhibit the most consistent directionality during migration.

Variation of MT numbers affects cell polarization and migration persistence

Next, we investigate how variations in MT number influence a cell's migration characteristics. Experimental studies suggest that changes in MT abundance can either enhance or impede directed locomotion (46–48). For instance, increased MT numbers have been linked to greater persistence in dendritic cells and cancer metastasis (46,48). We ran simulations with varying numbers of regular and long MTs, with total MT counts ranging from $N_{mt} = 25$ to 200. First, we examined the position of the centrosome for different values of N_{mt} in cells containing either regular or long MTs (Fig. 3 *A* and *B*). For regular MTs, as MT numbers increase, the centrosome becomes more prominently positioned ahead of the nucleus, in the direction of migration (Fig. 3 *A*). With more MTs nucleating from the centrosome, a greater number of MTs reach the cell front, enhancing the delivery of actin polymerization signals (compare Fig. S2 *A* and *B*). Consequently, cells with more MTs exhibit increased actin polymerization activity at the front. However, due to the relatively short length of regular MTs, most are unable to circumvent the nuclear membrane and extend to the membrane region behind the nucleus. This limits the actin polymerization signals at the rear cell membrane, making cell polarization depend solely on MT growth, and independent of MT number. Our findings further show that as MT numbers increase, the angle between the cell's direction of motion and the vector from the nucleus to the centrosome tends to be smaller (i.e., the centrosome is more often ahead of the nucleus) (Fig. 3 *A*). This suggests that in cells with regular MTs, higher MT numbers contribute to more stable protrusions at the membrane regions near the centrosome. The rear of the cell, receiving fewer polymerization signals, contracts through elastic coupling between the nucleus and the membrane. As a result, the cell tends to break symmetry and migrate, with the centrosome positioned ahead of the nucleus.

Our results demonstrate that cells with regular MTs exhibit more directed migration as the MT number increases. The displacement of the cell centroid was consistently greater for cells with $N_{mt} = 200$ compared with

$N_{mt} = 50$ at all time points (Fig. 3 *C*). Analysis of the MSD of the cell centroid revealed ballistic movement ($MSD \approx t^2$) for cells with $N_{mt} = 50 - 200$ (Fig. 3 *D*). Furthermore, the persistence length of migrating cells increased with MT numbers (Fig. 3 *E*), indicating that cells with more MTs maintained their polarization and were less likely to change the direction of migration. (46).

Next, we investigate the effect of MT numbers for long MTs ($l_{mt} = 16\,\mu m$). For low to intermediate MT numbers ($N_{mt} = 25 - 100$), the growing MT tips eventually reach the distal end of the membrane, where they deliver actin polymerization signals, and shrinking MTs spread contraction signals near the rear end of the cell. This establishes cell polarization through actin polymerization at the front supported by growing MT tips, and myosin contraction at the rear regulated by shrinking MT tips. When the MT number increased from 25 to 100, the probability distribution of the angle between the migration direction and the nucleus-to-centrosome vector shifts toward 180° (Fig. 3 *B*), indicating a stronger cell polarization. However, when the MT number becomes very high (~ 200), the polarity is reduced. A higher number of MTs leads to more growing tips occupying both the transverse and rear regions of the cell cortex, thereby reducing cell polarization. As a result, for $N_{mt} = 200$, actin polymerization signals appear more dispersed rather than polarized as observed for $N_{mt} = 50$ (see Fig. S2 *C* and *D*). With a large number of MTs, the cell no longer maintains a distinct anterior or posterior centrosome configuration during migration. The probability distribution of the angle between the migration direction and the nucleus-to-centrosome vector becomes more even, with no clear peak at any angle (Fig. 3 *B* (black line)). With a very large number of MTs ($N_{mt} = 200$), the cell continuously shifts its front and rear based on the actin polymerization signals received along each segment of the membrane, whether proximal or distal to the centrosome (see Fig. S2 *D*).

Analysis of the cell trajectories reveals that cells with long MTs exhibit more directed migration when the MT numbers are between $N_{mt} = 50 - 100$. The mean displacement of the cell centroid shows that cells with $N_{mt} = 50$ travel greater distances over time compared with those with $N_{mt} = 200$ (Fig. 3 *C*). The MSD of the cell centroid suggests that cells with $N_{mt} = 50$ migrate ballistically ($MSD \approx t^2$), whereas cells with $N_{mt} = 200$ exhibit super-diffusive motion ($MSD \approx t^{1.25}$) (Fig. 3 *D*). The persistence length of migrating cells increases with MT numbers within the range of $N_{mt} = 25 - 100$. However, for $N_{mt} = 200$, the persistence length sharply decreases to a very low value (Fig. 3 *F*). This shows that the persistence of directional locomotion increases with MT numbers for cells with long MTs ($l_{mt} = 16\,\mu m$), consistent with findings from various experiments (46). However, a very large number of MTs can impair cell locomotion when the centrosome is located behind the nucleus (see Video S2).

Anterior centrosome position improves directed migration in obstacle parks

We further examine the relationship between centrosome positioning and cell migration in obstacle parks, as explored experimentally in studies (e.g., Refs. (49,50)). These studies highlight that cells exhibiting directed migration on flat surfaces can become trapped when placed within obstacle parks. To understand how actin-MT crosstalk enables cells to navigate through restrictive geometries, we performed simulations with the cell placed in obstacle parks of varying obstacle sizes and spacings. To assess whether cell migration was directed or random, we calculated the local MSD of the cell centroid ($\Delta R^2(t_i)$) at regular time intervals of 300 s and examined its scaling exponent α as a function of the time lag ($\Delta R^2(t_i, \tau_k) = A\tau_k^{\alpha}$) (49). Additionally, we assessed the standard deviation of the velocity angle ($\Delta \phi_i$) at the corresponding time intervals. Directed migration was defined as $\alpha > 1.7$ and $\Delta \phi_i < 0.9$ (see supporting material for details) (49).

We first analyzed the trajectories of freely migrating cells. Our results indicate that freely migrating cells show significant counts of directed migration phases ($\alpha > 1.7$ and $\Delta \phi < 0.9$), both for regular and long MTs (Fig. 4 *A* and *B*). When the cells were placed in an obstacle park (obstacle radius $R_{obs} = 4\,\mu m$ with spacing $\Delta d = 20\,\mu m$), the frequency of directed migration decreased (Fig. 4 *C* and *D*; see Video S3). However, cells with regular MTs exhibit more directed migration phases in the obstacle park compared with those with long MTs. In cells with regular MTs, the centrosome is positioned ahead of the nucleus, allowing forward-growing MTs to explore alternative paths when encountering obstacles. This enables the cells to extend protrusions into gaps between obstacles and navigate through without fully changing direction. In contrast, cells with long MTs position their nucleus ahead of the centrosome, which can block MT extension toward adjacent pores when an obstacle is encountered. As a result, cells with long MTs tend to avoid obstacles and narrow pores, leading to a significant decrease in directed migration phases. Increasing the obstacle size ($R_{obs} = 5\,\mu m$) reduces the frequency of directed migration for both regular and long MT cells. However, cells with regular MTs are still able to maintain more directed migration compared with long MT cells, which are more likely to become trapped (see Fig. 4 *E* and *F*; see Video S4). On the other hand, increasing obstacle spacing to $\Delta d = 30\,\mu m$ leads to an increase in counts of directed migration phase (see Fig. 4 *G*). This indicates that when there is enough space for cells to pass through, they can maintain directed migration. Moreover, the mean displacement of the cell centroids revealed that cells with regular MTs, where the centrosome is positioned anterior to the nucleus, traveled greater distances in the obstacle park compared with long MT cells, regardless of obstacle size (Fig. 4 *H–J*).

Cell morphology altered by length and number of MTs

Next, we examined how the actin-MT crosstalk influences the morphology of the migrating cells, quantified by the cell's aspect ratio and spread area. When MTs are long ($l_{mt} = 16\,\mu m$), the aspect ratio remains close to 1 for all MT numbers, suggesting the cell shape is nearly circular (Fig. 5 *A*). In this configuration, the centrosome is preferentially positioned behind the nucleus, near the rear of the cell. MT tips extend along both the nuclear and cell membranes, reaching the opposite end of the cell, where they deliver actin polymerization signals that drive membrane protrusions. The region closer to the centrosome has a higher density of MTs, helping to resist membrane contraction. This balance leads to a roughly circular cell shape. However, when the average MT length is shorter (e.g., regular MT length with $l_{mt} = 9.3\,\mu m$), the aspect ratio varies between 1.5 and 3.5 for $N_{mt} = 50$ (Fig. 5 *A*). As MT numbers increase ($N_{mt} = 100, 200$), the aspect ratio stabilizes around 1.5. This indicates that the cell shape deviates from a circular form when $l_{mt} = 9.3\,\mu m$. In this scenario, the centrosome remains positioned ahead of the nucleus, closer to the front of the cell membrane. However, the MTs fail to navigate around the nuclear membrane to reach the opposite end of the cell. Consequently, actin polymerization signals are not delivered to the membrane region behind the nucleus. Thus, membrane protrusions only occur at the front and side regions of the cell membrane, where MTs are able to grow. In contrast, the rear of the membrane receives fewer MTs and experiences contraction forces from the membrane-nucleus coupling. The combination of extension at the front and sides, alongside contraction at the rear, leads to the cell deviating from its circular shape.

Next, we investigated the spread area of the cells for various MT numbers and lengths. Actin polymerization activity at the cortex, driven by MT delivered polymerization signals, increases the spread area of the cell. Our results indicated that cells with long MTs have a larger spread area compared with cells with regular MTs (Fig. 5 *B*). Regular MTs deliver actin polymerization signals only at the membrane regions close to the centrosome. However, long MTs deliver actin polymerization signals throughout the cell membrane, resulting in longer protrusions and increased spread area of the cell. As MT numbers increase, the actin polymerization signals at the actin cortex also increase. Thus, an increase in MT numbers corresponds to an increase in cell area for regular and long MTs.

Next, we analyzed the position of the nucleus relative to the cell center. For cells with regular MTs, the nucleus remains closest to the cell center when $N_{mt} = 50$ (Fig. 5 *C*). As the number of MTs increases, the distance between the nucleus and the cell center also increases. In these cells, the centrosome is positioned anterior to the nucleus. As the MT count rises, the pushing force exerted on the nucleus grows stronger, shifting the nucleus toward the rear of the cell and away from the center (Fig. 5 *C*). In contrast, for cells with long MTs, the nucleus moves closer to the cell center as the number of MTs increases (Fig. 5 *C*). In these cases, the centrosome remains positioned posterior to the nucleus. With more MTs, the increased pushing force on the nucleus results in its movement toward the cell center.

Finally, we examine the distance of the centrosome from the cell center. For cells with regular MTs, the centrosome remains closer to the cell center (Fig. 5 *D*). When the centrosome is positioned anterior to the nucleus, our results indicate that it tends to stay near the cell center. The nucleus is slightly displaced toward the rear of the cell due to the pushing forces exerted by the MTs (Fig. 5 *E*). Many regular MTs buckle at the cell membrane, and the resulting forces push the centrosome toward the cell center. For cells with long MTs, most of the MTs slide along the cell or nuclear membrane to the cell front. Consequently, the resultant pushing force on the centrosome is insufficient to keep it near the center. As a result, the centrosome remains toward the rear of the cell, whereas the nucleus moves closer to the center (Fig. 5 *D* and *F*). As the number of long MTs increases, the pushing forces on the centrosome intensify, causing it to shift slightly closer to the center at $N_{mt} = 100$ as compared with $N_{mt} = 50$. However, with further increase in MT numbers, the cell loses its polarity, and the centrosome's position becomes more random within the cell.

MT actomyosin crosstalk influences cell path at Y-junctions

Inspired by experimental observations linking cell migration paths to centrosome positioning at Y-junctions (21), we placed our model cell in a comparable setup, as depicted in Fig. 6 *A* and *B*. To replicate the chemotactic gradient applied in the experiments to drive forward migration, we impose a small forward velocity on the membrane beads. Initially, the channel widths are kept equal, larger than the nucleus diameter ($R_{nuc} = 3\,\mu m$), but smaller than the cell diameter ($R_{cell} = 7\,\mu m$), set at $10\,\mu m$. We consider that a cell has moved into a channel when the entire cell has traveled at least $5.0\mu m$ into a channel. Our results show that migrating cells with long MTs and the centrosome positioned behind the nucleus take significantly longer to enter the channel compared with cells with regular MTs and the centrosome ahead of the nucleus (Fig. 6 *C*). Cells with regular MTs enter the channel with the centrosome positioned ahead of the nucleus. MTs growing from the centrosome can reach the protrusion tip without obstruction, enhancing actin polymerization and promoting faster entry into the channel (see Fig. S3 *A*). In contrast, cells with long MTs have the centrosome located behind the nucleus, which enters the channel first (see Fig. S3 *B*). The nucleus obstructs the entry of newly nucleated MTs into the protrusion, resulting in

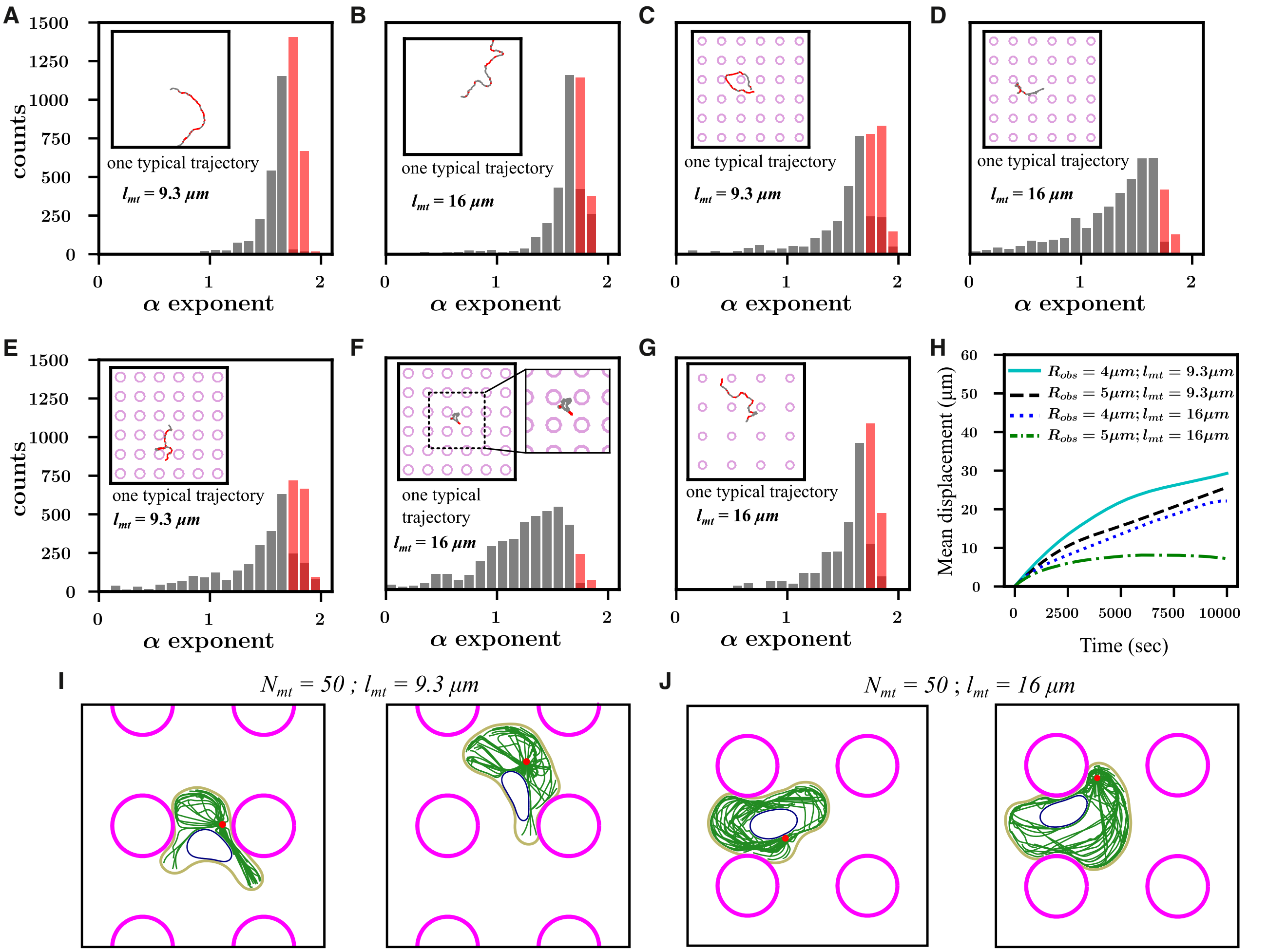

FIGURE 4 Cell migration through obstacles. (*A and B*) Distribution of local MSD exponent α for free cell migration for $l_{mt} = 9.3\ \mu m$ and $16\ \mu m$ with 50 MTs. Gray bars represent counts for random migration, whereas red bars indicate counts for directed migration. (*C and D*) Distribution of exponent α for cell migration in obstacle park with obstacle radius $R_{obs} = 4\ \mu m$ and obstacle spacing $\Delta d = 20\ \mu m$ for $l_{mt} = 9.3\ \mu m$ and $16\ \mu m$. (*E and F*) Distribution of α with larger obstacles having radius $R_{obs} = 5\ \mu m$ and obstacle spacing $\Delta d = 20\ \mu m$ for $l_{mt} = 9.3\ \mu m$ and $16\ \mu m$. (*G*) Distribution of α with obstacle radius $R_{obs} = 4\ \mu m$ and larger obstacle spacing $\Delta d = 30\ \mu m$ for $l_{mt} = 16\ \mu m$. All trajectories shown in insets start at the center of the frame of size $60\ \mu m \times 60\ \mu m$. Y-axis range is same for (*A*)–(*G*). (*H*) Mean displacement of the cell centroid over time for various obstacle sizes and MT lengths. (*I*) Snapshots of cell configuration 25 simulation minutes apart for the case shown in (*E*). Cell manages to squeeze through obstacles when centrosome is ahead of the nucleus. (*J*) Snapshots of cell configuration 25 simulation minutes apart for the case shown in (F). Cell remains stuck between obstacles when centrosome is behind the nucleus.

reduced actin polymerization at the front and slower overall entry into the channel. For these equal-width channels, the probability of moving into either edge of the Y-junction is close to 50% for both regular and long MTs (Fig. 6 *D* and *E*), and for regular MTs, the cell always took the path that the centrosome entered first, in agreement with the experimental observations (21).

Next, we simulated cells facing a Y-junction with wide ($10\ \mu m$) and narrow ($6\ \mu m$) channels (Fig. 6 *F*). Our results indicate that cells with long MTs take more time to move into either the narrow or wide channel compared with cells with regular MTs. However, when the width of the two channels is unequal, the time taken by cells with long MTs to choose a channel decreases (compare Fig. 6 *C* and *G*). This suggests that when one path is much more restrictive than the other, cells with their nucleus ahead of the centrosome retract their protrusion from the restrictive path faster. When both the path choices are similar, the cell takes more time to retract its protrusion from one path and move completely into the other channel.

For migrating cells with long MTs ($l_{mt} = 16\ \mu m$) and the nucleus ahead of the centrosome, cells prefer to move into the wider channel (Fig. 6 *H*). This behavior is consistent with experimental observations suggesting that cells use their nucleus to gauge pore sizes and choose the path of least resistance (21). Cells with regular MTs ($l_{mt} = 9.3\ \mu m$) and the centrosome positioned ahead of the nucleus exhibit a higher tendency to enter the narrow pore compared with cells with long MTs (Fig. 6 *I*). This result is in qualitative agreement with experimental observations of fibroblasts

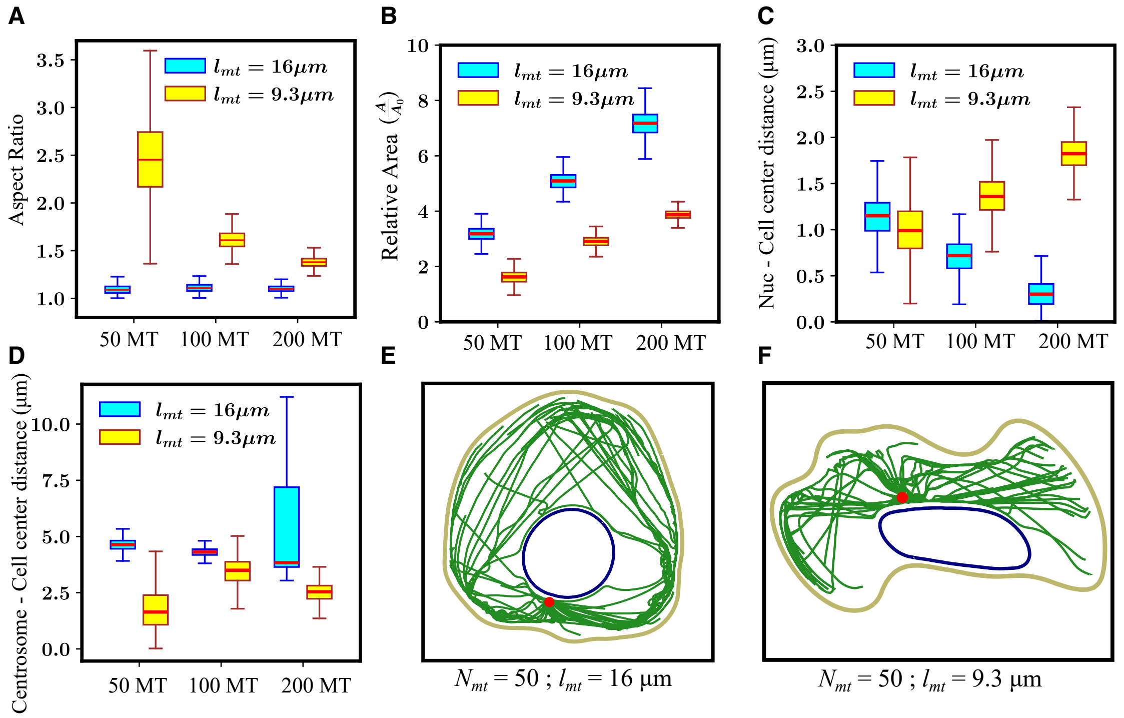

FIGURE 5 MT length and numbers influence cell morphology. (*A*) Aspect ratio of cells with various MT lengths and numbers. Cells with long MTs have an aspect ratio ~ 1 (*circular shape*). Regular MTs having $l_{mt} = 9.3\ \mu m$ lead to a high aspect ratio when the MT number is low ($N_{mt} = 50$). (*B*) Area of the migrating cell relative to initial area ($A_0 = \pi R_{cell}^2$) for various MT lengths and numbers. Cell area increases monotonically with MT numbers and average length. (*C*) Distance between the nucleus and the cell center for varying MT lengths and numbers. (*D*) Distance of centrosome from cell center for various MT lengths and numbers. (*E and F*) Snapshots of migrating cells for $l_{mt} = 9.3\ \mu m$ and $16\ \mu m$ with $N_{mt} = 50$.

and dendritic cells navigating bifurcating channels of different widths (21). However, these cells still have a higher probability of moving into the wider pore over the narrower one (Fig. 6 *I*). This may be attributed to the fact that, with the centrosome ahead of the nucleus, growing MTs can easily extend into the protrusion within the smaller channel, facilitating actin polymerization. In contrast, cells with long MTs, where the nucleus is positioned ahead of the centrosome, experience blockage from the nucleus, preventing the MTs from reaching the smaller channel. As a result, membrane protrusions in the smaller channel are less stabilized and eventually retract.

Finally, we examined the behavior of cells at a junction with multiple path choices. We simulated cells with varying MT lengths within a geometry containing four paths, each with different channel widths (Fig. 6 *J*). Our results indicate that cells with long MTs ($l_{mt} = 16\ \mu m$) explore all available paths and only enter channels with wider pores (channel width $= 8\ \mu m$ and $6\ \mu m$), whereas no cells moved into channels with very narrow pores (channel width $= 4\ \mu m$ and $2\ \mu m$) (Fig. 6 *K*; see Video S5). The majority of the cells ($\approx 90\%$) moved into the widest pore (width $= 8\ \mu m$), indicating a clear preference for the path of least resistance. In contrast, cells with regular MTs ($l_{mt} = 9.3\ \mu m$) did not show a strong preference for the widest pore, with about $\approx 40\%$ of cells moving into the $6\mu m$ channel (Fig. 6 *L*; see Video S5). Nearly 20% of cells moved into the $4\mu m$ channel, whereas no cells moved into the $2\mu m$ channel. These results suggest that with the centrosome positioned ahead of the nucleus, MTs can extend further into smaller pores, stabilizing protrusions and enabling the cell to squeeze through. Similar path selection behavior, consistent with our simulation results, has been observed in experimental studies across different cell types, where the MTOC is positioned either ahead of or behind the nucleus at channel junctions (21).

CONCLUSION

Motivated by recent experiments reporting strong correlations between centrosome positioning and migration characteristics and path choices, we presented in this work a mechanistic whole-cell model that integrates basic aspects of actin-MT crosstalk, namely growing (shrinking) MTs delivering polymerization (contraction) signals locally to actomyosin. Our model shows that the position of the centrosome, anterior or posterior to the nucleus, corresponds to different arrangements of the MT array within the cell. This provides different pathways for cell polarization through actin polymerization and myosin contraction signals. Our in silico results indicate that the position of the centrosome depends on the average lengths of the MTs

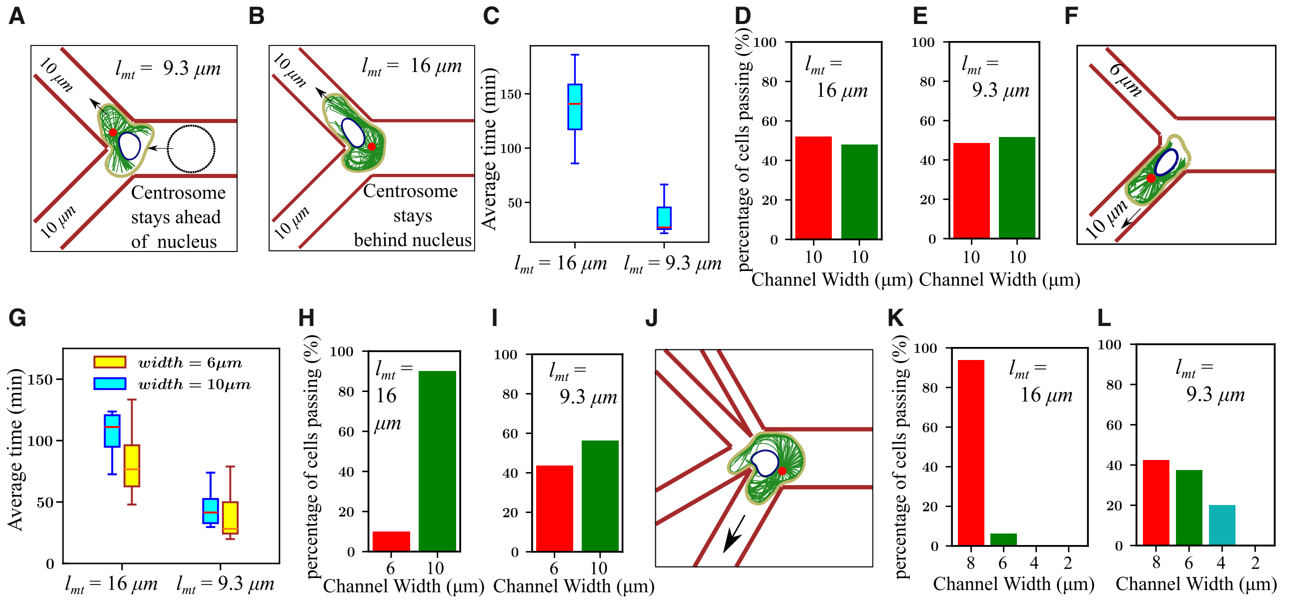

FIGURE 6 Cell migration in a Y-shaped channel (*A and B*) Snapshots show cells navigating Y-junction channels of equal width (10 μm) for $l_{mt} = 9.3$ μm and 16 μm. In (*A*), the dotted line marks the initial cell position; arrows indicate migration direction. (*C*) Time required for cells with $N_{mt} = 100$ to traverse the junction. (*D*) Percentage of cells with long MTs ($l_{mt} = 16$ μm) passing through each 10 *μm channel*. (*E*) Percentage of cells with regular MTs ($l_{mt} = 9.3$ μm) choosing each 10 *μm channel*. (*F*) Snapshot of a cell encountering a wide (10 μm) and narrow (6 μm) pore at the junction. (*G*) Migration time for cells with different MT lengths entering wide or narrow pores. (*H*) Percentage of cells with long MTs migrating through wide and narrow pores. (*I*) Percentage of cells with regular MTs selecting wide or narrow pores. (*J*) Snapshot of a cell in a device offering four channels of varying widths. (*K*) Percentage of cells with long MTs passing through each pore size. (*L*) Percentage of cells with regular MTs selecting among the different pore widths. All simulations were performed for $N_{mt} = 100$.

(Fig. 2 *B*). Regular MTs, with an average length of two-thirds of the cell diameter, place the centrosome mostly ahead of the nucleus, whereas long MTs with an average length greater than the cell diameter place the centrosome behind the nucleus in the direction of migration.

The migrating cell can also change from ballistic to diffusive motion by adjusting its cortical actin dynamics through changes in the distribution of regulatory signals reaching the cortex. Earlier studies have reported that variations in MT numbers can alter the characteristics of cell migration (46,47,51). Our model also suggests that changes in the number of MTs can lead to different cell migratory behaviors. In a centrosome anterior to the nucleus configuration, an increase in the number of MTs leads to a significant increase in persistence (Fig. 3 *D*). In the centrosome posterior to the nucleus configuration, increasing MT numbers initially lead to ballistic migration, but the motion becomes super-diffusive when the number of MTs is too high (Fig. 3 *C*).

When migrating through complex tissue geometries, cells encounter obstacles formed by the surrounding extracellular matrix and neighboring tissues. At junctions, cells develop highly branched morphologies, extending protrusions to probe narrow channels and select an optimal path. Eventually, one protrusion stabilizes and guides the cell, whereas others are retracted (52). MTs are crucial in this selection: the successful protrusion is reinforced by MT-mediated delivery of actin polymerization signals, whereas competing protrusions retract following cortical contraction cues (6). Experimental studies have shown a marked increase in cell passage time and instances of cell fragmentation with disintegration of protrusions from cell body at Y-channel junctions after complete MT depolymerization (21). Complete depolymerization of MTs in our model caused migratory failure, and cells remained stuck at channel junctions (see Fig. S4 *A–C*).

Experimental studies on *Dictyostelium discoideum* and leukocytes navigating pillar arrays and Y-junctions suggest that centrosome positioning, either anterior or posterior to the nucleus, influences path choice (20,21). Cells also tend to migrate away from regions densely packed with obstacles (53). Our simulation results predict that with the centrosome anterior to the nucleus, cells could migrate more robustly in obstacle parks (Fig. 4 *C*). At a Y-junction, cells with the centrosome positioned anterior to the nucleus show only a slight preference for the wider channel, whereas cells with a posterior centrosome almost always migrate through the wider channel (Fig. 6 *G*). We also observed that increased cell membrane stiffness and a softer nucleus can improve cell migration in obstacle parks (see Fig. S5). Further, cells in obstacle parks were found to migrate toward regions of sparse obstacle density (see Fig. S6).

Our results indicate that changes in the physical properties of MTs, such as their average length and number, can influence cellular migration. Future experimental studies investigating how cells regulate MT organization to adapt

their migratory behavior in both unconfined and confined geometries would provide valuable insights into this mechanism.

Our results offer insights into how immune cells navigate tissues, how cancer cells start to metastasize, and how cells migrate within synthetic confined geometries. This model represents an initial step toward integrating key aspects of actin-MT crosstalk into a mechanistic framework for cell migration, and it already captures a range of self-organized behaviors, including centrosome positioning, transitions between random and ballistic motion, and path selection in confined spaces. However, the model involves several simplifications: for instance, the closed-loop bead-spring representation of the membrane does not fully capture the complexity of real membranes, and parameters such as stretching and buckling stiffness cannot be directly mapped to experimental measurements. Similarly, dynein-MT interactions at the cortex are simplified through spring attachments between discrete beads. To match experimental observations, we selected parameter values that best reproduced known behaviors.

Our study focuses on the role of MT dynamics in establishing and maintaining cell polarization, as well as guiding path selection in both free and confined geometries when the cell encounters obstacles. The simulation timescales were chosen to effectively capture MT-driven processes relevant to polarization and path guidance under these conditions. However, the simulations do not capture the transition from ballistic to diffusive migration observed over longer timescales. Modeling this transition would require significantly more extensive and computationally demanding simulations.

Our model assumes a constant average MT length, which does not fully align with experimental observations. In dendritic cells, where the centrosome is positioned behind the nucleus, polarization is supported by longer, more stable MTs extending toward the leading edge and shorter, less stable MTs oriented toward the rear (6,13). This organization produces an elongated, elliptical morphology that our model cannot capture, as it lacks a mechanism for stable polarization with an asymmetric MT length distribution. Experimental studies have demonstrated feedback between actin polymerization and growing MT tips at the leading edge of migrating cells (54). MTs can be anchored to the actin cortex at the front or pushed back as a result of actin retrograde flow during polymerization. Our model does not incorporate mechanical anchoring of MT tips at the leading edge or the pushing forces exerted by actin retrograde flow. Including such feedback mechanisms could influence the spatial distribution of MT tips at the cell cortex.

Our model uses a fixed number of beads for the cell and nuclear membranes, along with a relatively large stiffness parameter between adjacent beads. This limits large variations in cell perimeter. Fibroblasts and neutrophils have been observed to maintain a relatively constant contact area and perimeter on soft substrates (55,56). However, their contact area and perimeter can increase significantly on stiff substrates (55–57). Our present model does not capture such large deviations in cell perimeter.

MT-mediated chemical signaling is not the sole pathway for cell polarization. Alternative mechanisms enabling polarization and subsequent migration in the absence of MTs have also been reported in the literature, which have not been accounted for in our model (58,59). Investigating mechanisms that enable such stable polarization and incorporating them into the model would be an interesting direction for future work.

Cells are also known to exhibit variations in MT stability in response to external chemical or mechanical cues (6,13,60,61). Moreover, factors such as the arrangement of the extracellular matrix and the density of adhesion molecules influence migratory behavior (62–64). Cells migrating in 3D extracellular matrix environments often display different characteristics compared with those migrating on 2D surfaces, a distinction that simple 2D models cannot fully represent. Future studies could explore how cells migrating in both 2D and 3D environments sense external cues, such as chemical gradients and matrix organization, to guide their migration. Understanding the role of MTs and actin-MT crosstalk in these sensing mechanisms would be an interesting area for further investigation.

ACKNOWLEDGMENTS

P.N. was supported by a fellowship from CSIR, India. H.R. acknowledges financial support by the German Research Foundation (DFG), project number 468346334. R.P. thanks IACS for funding and computational facilities.

AUTHOR CONTRIBUTIONS

H.R. conceived the study; P.N., A.K.D., R.P., and H.R. designed research and developed computational models; P.N. performed simulations; P.N., A.K.D., R.P., and H.R. analyzed data and wrote the paper. All authors contributed to editing and revisions of the paper.

DECLARATION OF INTERESTS

The authors declare no competing interests.

SUPPORTING CITATIONS

References (65–84) appear in the supporting material.

SUPPORTING MATERIAL

Supporting material can be found online at https://doi.org/10.1016/j.bpj.2025.09.029.

REFERENCES

1. Mueller, J., G. Szep, ..., M. Sixt. 2017. Load Adaptation of Lamellipodial Actin Networks. *Cell.* 171:188–200.e16.

2. Hu, Y.-L., S. Lu, …, S. Chien. 2014. FAK and paxillin dynamics at focal adhesions in the protrusions of migrating cells. *Sci. Rep.* 4:6024.
3. Nobes, C. D., and A. Hall. 1995. Rho, Rac, and Cdc42 GTPases regulate the assembly of multimolecular focal complexes associated with actin stress fibers, lamellipodia, and filopodia. *Cell.* 81:53–62.
4. Ridley, A. J., and A. Hall. 1992. Distinct patterns of actin organization regulated by the small GTP-binding proteins Rac and Rho. *Cold Spring Harbor Symp. Quant. Biol.* 57:661–671.
5. Zhang, J., and Y.-l. Wang. 2017. Centrosome defines the rear of cells during mesenchymal migration. *Mol. Biol. Cell.* 28:3240–3251.
6. Kopf, A., J. Renkawitz, …, M. Sixt. 2020. Microtubules control cellular shape and coherence in amoeboid migrating cells. *J. Cell Biol.* 219:e201907154.
7. Lavrsen, K., G. Rajendraprasad, …, M. Barisic. 2023. Microtubule detyrosination drives symmetry breaking to polarize cells for directed cell migration. *Proc. Natl. Acad. Sci. USA.* 120:e2300322120.
8. Siegrist, S. E., and C. Q. Doe. 2007. Microtubule-induced cortical cell polarity. *Genes Dev.* 21:483–496.
9. Li, R., and G. G. Gundersen. 2008. Beyond polymer polarity: how the cytoskeleton builds a polarized cell. *Nat. Rev. Mol. Cell Biol.* 9:860–873.
10. Henty-Ridilla, J. L., A. Rankova, …, B. L. Goode. 2016. Accelerated actin filament polymerization from microtubule plus ends. *Science.* 352:1004–1009.
11. Ueda, M., R. Gräf, …, U. Euteneuer. 1997. Centrosome positioning and directionality of cell movements. *Proc. Natl. Acad. Sci. USA.* 94:9674–9678.
12. Takesono, A., S. J. Heasman, …, A. J. Ridley. 2010. Microtubules Regulate Migratory Polarity through Rho/ROCK Signaling in T Cells. *PLoS One.* 5:e8774.
13. Kopf, A., and E. Kiermaier. 2021. Dynamic microtubule arrays in leukocytes and their role in cell migration and immune synapse formation. *Front. Cell Dev. Biol.* 9:635511.
14. Garcin, C., and A. Straube. 2019. Microtubules in cell migration. *Essays Biochem.* 63:509–520.
15. Zhang, J., W.-H. Guo, and Y.-L. Wang. 2014. Microtubules stabilize cell polarity by localizing rear signals. *Proc. Natl. Acad. Sci. USA.* 111:16383–16388.
16. Liang, E. I., E. J. Mah, …, M. A. Digman. 2017. Correlation of focal adhesion assembly and disassembly with cell migration on nanotopography. *Integr. Biol.* 9:145–155.
17. Hu, Y., J. Lu, …, H. Zhang. 2017. Regulation of focal adhesion turnover in SDF-1α-stimulated migration of mesenchymal stem cells in neural differentiation. *Sci. Rep.* 7:10013.
18. Ridley, A. J., and A. Hall. 1992. The small GTP-binding protein rho regulates the assembly of focal adhesions and actin stress fibers in response to growth factors. *Cell.* 70:389–399.
19. Raab, M., and D. E. Discher. 2017. Matrix rigidity regulates microtubule network polarization in migration. *Cytoskeleton.* 74:114–124.
20. Ishikawa-Ankerhold, H., J. Kroll, …, A. Müller-Taubenberger. 2022. Centrosome positioning in migrating dictyostelium cells. *Cells.* 11:1776.
21. Renkawitz, J., A. Kopf, …, M. Sixt. 2019. Nuclear positioning facilitates amoeboid migration along the path of least resistance. *Nature.* 568:546–550.
22. Mallick, A., A. Sarkar, and R. Paul. 2022. A force-balance model for centrosome positioning and spindle elongation during interphase and anaphase B. *Indian J. Phys.* 96:2667–2691.
23. Letort, G., F. Nédélec, …, M. Théry. 2016. Centrosome centering and decentering by microtubule network rearrangement. *Mol. Biol. Cell.* 27:2833–2843.
24. Oelz, D. B., U. del Castillo, …, A. Mogilner. 2018. Microtubule Dynamics, Kinesin-1 Sliding, and Dynein Action Drive Growth of Cell Processes. *Biophys. J.* 115:1614–1624.
25. Laan, L., S. Roth, and M. Dogterom. 2012. End-on microtubule-dynein interactions and pulling-based positioning of microtubule organizing centers. *Cell Cycle.* 11:3750–3757.
26. Zhu, J., A. Burakov, …, A. Mogilner. 2010. Finding the Cell Center by a Balance of Dynein and Myosin Pulling and Microtubule Pushing: A Computational Study. *Mol. Biol. Cell.* 21:4418–4427.
27. Larripa, K., and A. Mogilner. 2006. Transport of a 1D viscoelastic actin–myosin strip of gel as a model of a crawling cell. *Physica A.* 372:113–123.
28. Barnhart, E., K.-C. Lee, …, A. Mogilner. 2015. Balance between cell substrate adhesion and myosin contraction determines the frequency of motility initiation in fish keratocytes. *Proc. Natl. Acad. Sci. USA.* 112:5045–5050.
29. Flommersfeld, J., S. Stöberl, …, C. P. Broedersz. 2024. Geometry-Sensitive Protrusion Growth Directs Confined Cell Migration. *Phys. Rev. Lett.* 132:098401.
30. Fogelson, B., and A. Mogilner. 2018. Actin-Myosin Force Generation and Symmetry Breaking in the Model Contractile Fiber. *SIAM J. Appl. Math.* 78:1754–1777.
31. Abaurrea-Velasco, C., T. Auth, and G. Gompper. 2019. Vesicles with internal active filaments: self-organized propulsion controls shape, motility, and dynamical response. *New J. Phys.* 21:123024.
32. Abaurrea Velasco, C., S. Dehghani Ghahnaviyeh, …, G. Gompper. 2017. Complex self-propelled rings: a minimal model for cell motility. *Soft Matter.* 13:5865–5876.
33. Chugh, P., A. G. Clark, …, E. K. Paluch. 2017. Actin cortex architecture regulates cell surface tension. *Nat. Cell Biol.* 19:689–697.
34. Tinevez, J.-Y., U. Schulze, …, E. Paluch. 2009. Role of cortical tension in bleb growth. *Proc. Natl. Acad. Sci. USA.* 106:18581–18586.
35. Sadhukhan, S., C. Martinez-Torres, …, N. Gov. 2025. Modeling how lamellipodia-driven cells maintain persistent migration and interact with external barriers. *Phys. Rev. Res.* 7:013319.
36. Sadhu, R. K., S. Penič, …, N. S. Gov. 2021. Modelling cellular spreading and emergence of motility in the presence of curved membrane proteins and active cytoskeleton forces. *Eur. Phys. J. A.* 136:495.
37. Lavi, I., M. Piel, …, N. S. Gov. 2016. Deterministic patterns in cell motility. *Nat. Phys.* 12:1146–1152.
38. Brückner, D. B., M. Schmitt, …, C. P. Broedersz. 2022. Geometry Adaptation of Protrusion and Polarity Dynamics in Confined Cell Migration. *Phys. Rev. X.* 12:031041.
39. Dogterom, M., and G. H. Koenderink. 2019. Actin–microtubule crosstalk in cell biology. *Nat. Rev. Mol. Cell Biol.* 20:38–54.
40. Hornak, I., and H. Rieger. 2020. Stochastic Model of T Cell Repolarization during Target Elimination (I). *Biophys. J.* 118:1733–1748.
41. Hornak, I., and H. Rieger. 2022. Stochastic model of T cell repolarization during target elimination (II). *Biophys. J.* 121:1246–1265.
42. Sens, P. 2020. Stick-slip model for actin-driven cell protrusions, cell polarization, and crawling. *Proc. Natl. Acad. Sci. USA.* 117:24670–24678.
43. Stöberl, S., J. Flommersfeld, …, J. O. Rädler. 2024. Nuclear deformation and dynamics of migrating cells in 3D confinement reveal adaptation of pulling and pushing forces. *Sci. Adv.* 10:eadm9195.
44. Maniotis, A. J., C. S. Chen, and D. E. Ingber. 1997. Demonstration of mechanical connections between integrins, cytoskeletal filaments, and nucleoplasm that stabilize nuclear structure. *Proc. Natl. Acad. Sci. USA.* 94:849–854.
45. Lou, S. S., A. Diz-Muñoz, …, J. A. Theriot. 2015. Myosin light chain kinase regulates cell polarization independently of membrane tension or Rho kinase. *JCB (J. Cell Biol.).* 209:275–288.
46. Weier, A.-K., M. Homrich, …, E. Kiermaier. 2022. Multiple centrosomes enhance migration and immune cell effector functions of mature dendritic cells. *J. Cell Biol.* 221:e202107134.
47. Rich, A. M., and S. T. Hoffstein. 1981. Inverse correlation between neutrophil microtubule numbers and enhanced random migration. *J. Cell Sci.* 48:181–191.

48. Ogden, A., P. C. G. Rida, and R. Aneja. 2013. Heading off with the herd: how cancer cells might maneuver supernumerary centrosomes for directional migration. *Cancer Metastasis Rev.* 32:269–287.
49. Arcizet, D., S. Capito, …, D. Heinrich. 2012. Contact-controlled amoeboid motility induces dynamic cell trapping in 3D-microstructured surfaces. *Soft Matter.* 8:1473–1481.
50. Shaebani, M. R., R. Jose, …, F. Lautenschläger. 2020. Persistence-Speed Coupling Enhances the Search Efficiency of Migrating Immune Cells. *Phys. Rev. Lett.* 125:268102.
51. Glasgow, J. E., and R. P. Daniele. 1994. Role of microtubules in random cell migration : stabilization of cell polarity. *Cytoskeleton.* 27:88–96.
52. Ron, J. E., M. Crestani, …, N. S. Gov. 2024. Emergent seesaw oscillations during cellular directional decision-making. *Nat. Phys.* 20:501–511.
53. Wondergem, J. A. J., M. Mytiliniou, …, D. Heinrich. 2019. Chemotaxis and topotaxis add vectorially for amoeboid cell migration. Preprint at bioRxiv. https://doi.org/10.1101/735779.
54. Waterman-Storer, C. M., and E. D. Salmon. 1997. Actomyosin-based retrograde flow of microtubules in the lamella of migrating epithelial cells influences microtubule dynamic instability and turnover and is associated with microtubule breakage and treadmilling. *J. Cell Biol.* 139:417–434.
55. Oakes, P. W., T. C. Bidone, …, M. L. Gardel. 2018. Lamellipodium is a myosin-independent mechanosensor. *Proc. Natl. Acad. Sci. USA.* 115:2646–2651.
56. Oakes, P. W., D. C. Patel, …, J. X. Tang. 2009. Neutrophil morphology and migration are affected by substrate elasticity. *Blood.* 114:1387–1395.
57. Suraneni, P., B. Rubinstein, …, R. Li. 2012. The Arp2/3 complex is required for lamellipodia extension and directional fibroblast cell migration. *J. Cell Biol.* 197:239–251.
58. Euteneuer, U., and M. Schliwa. 1984. Persistent, directional motility of cells and cytoplasmic fragments in the absence of microtubules. *Nature.* 310:58–61.
59. Edelstein-Keshet, L., W. R. Holmes, …, M. Dutot. 2013. From simple to detailed models for cell polarization. *Philos. Trans. R. Soc. Lond. B Biol. Sci.* 368:20130003.
60. Espina, J. A., C. L. Marchant, and E. H. Barriga. 2022. Durotaxis: the mechanical control of directed cell migration. *FEBS J.* 289:2736–2754.
61. SenGupta, S., C. A. Parent, and J. E. Bear. 2021. The principles of directed cell migration. *Nat. Rev. Mol. Cell Biol.* 22:529–547.
62. Winkler, J., A. Abisoye-Ogunniyan, …, Z. Werb. 2020. Concepts of extracellular matrix remodelling in tumour progression and metastasis. *Nat. Commun.* 11:5120.
63. Ray, A., O. Lee, …, P. P. Provenzano. 2017. Anisotropic forces from spatially constrained focal adhesions mediate contact guidance directed cell migration. *Nat. Commun.* 8:14923.
64. Sadjadi, Z., D. Vesperini, …, H. Rieger. 2022. Ameboid cell migration through regular arrays of micropillars under confinement. *Biophys. J.* 121:4615–4623.
65. Liao, G., T. Nagasaki, and G. G. Gundersen. 1995. Low concentrations of nocodazole interfere with fibroblast locomotion without significantly affecting microtubule level: implications for the role of dynamic microtubules in cell locomotion. *J. Cell Sci.* 108:3473–3483.
66. Lee, S. Y., P. W. A. Schönhöfer, and S. C. Glotzer. 2023. Complex motion of steerable vesicular robots filled with active colloidal rods. *Sci. Rep.* 13:22773.
67. Koonce, M. P., and A. Khodjakov. 2002. Dynamic microtubules in Dictyostelium. *J. Muscle Res. Cell Motil.* 23:613–619.
68. Schliwa, M., K. B. Pryzwansky, and U. Euteneuer. 1982. Centrosome splitting in neutrophils: an unusual phenomenon related to cell activation and motility. *Cell.* 31:705–717.
69. Schulze, E., and M. Kirschner. 1986. Microtubule dynamics in interphase cells. *J. Cell Biol.* 102:1020–1031.
70. Ryder, M. I., R. N. Weinreb, and R. Niederman. 1988. Microtubule-granule relationships in motile human polymorphonuclear leukocytes. *Anat. Rec.* 221:679–686.
71. Murphy, P. 1976. Morphology and Cellular Physiology of Neutrophil Granulocytes. Springer US, Boston, MA, pp. 17–31.
72. Lammerding, J. 2011. Mechanics of the Nucleus. John Wiley & Sons, Ltd, pp. 783–807.
73. van Haren, J., and T. Wittmann. 2019. Microtubule Plus End Dynamics "-" Do We Know How Microtubules Grow? *Bioessays.* 41:1800194.
74. Zwetsloot, A. J., G. Tut, and A. Straube. 2018. Measuring microtubule dynamics. *Essays Biochem.* 62:725–735.
75. Dogterom, M., and S. Leibler. 1993. Physical Aspects of the Growth and Regulation of Microtubule Structures. *Phys. Rev. Lett.* 70:1347–1350.
76. Ron, J. E., P. Monzo, …, N. S. Gov. 2020. One-dimensional cell motility patterns. *Phys. Rev. Res.* 2:033237.
77. Kirschner, M., and T. Mitchison. 1986. Beyond self-assembly: From microtubules to morphogenesis. *Cell.* 45:329–342.
78. Walker, R. A., E. T. O'Brien, …, E. D. Salmon. 1988. Dynamic instability of individual microtubules analyzed by video light microscopy: rate constants and transition frequencies. *J. Cell Biol.* 107:1437–1448.
79. Vutukuri, H. R., M. Hoore, …, J. Vermant. 2020. Active particles induce large shape deformations in giant lipid vesicles. *Nature.* 586:52–56.
80. Wang, C., Y.-k. Guo, …, K. Chen. 2019. Shape transformation and manipulation of a vesicle by active particles. *J. Chem. Phys.* 150:044907.
81. Soheilypour, M., M. Peyro, …, M. R. K. Mofrad. 2015. Buckling Behavior of Individual and Bundled Microtubules. *Biophys. J.* 108:1718–1726.
82. Ezber, Y., V. Belyy, …, A. Yildiz. 2020. Dynein harnesses active fluctuations of microtubules for faster movement. *Nat. Phys.* 16:312–316.
83. Wang, H., Z. Jia, and Y. Fang. 2024. Chemo-mechanical model of cell polarization initiated by structural polarity. *Soft Matter.* 20:8407–8419.
84. Kim, D.-H., and D. Wirtz. 2013. Focal adhesion size uniquely predicts cell migration. *FASEB J.* 27:1351–1361.

Biophysical Journal, Volume *124*

Supplemental information

Modeling actin-microtubule crosstalk in migrating cells

Pinaki Nayak, Anil Kumar Dasanna, Raja Paul, and Heiko Rieger

Supplemental Information

SUPPLEMENTAL FIGURES AND LEGENDS

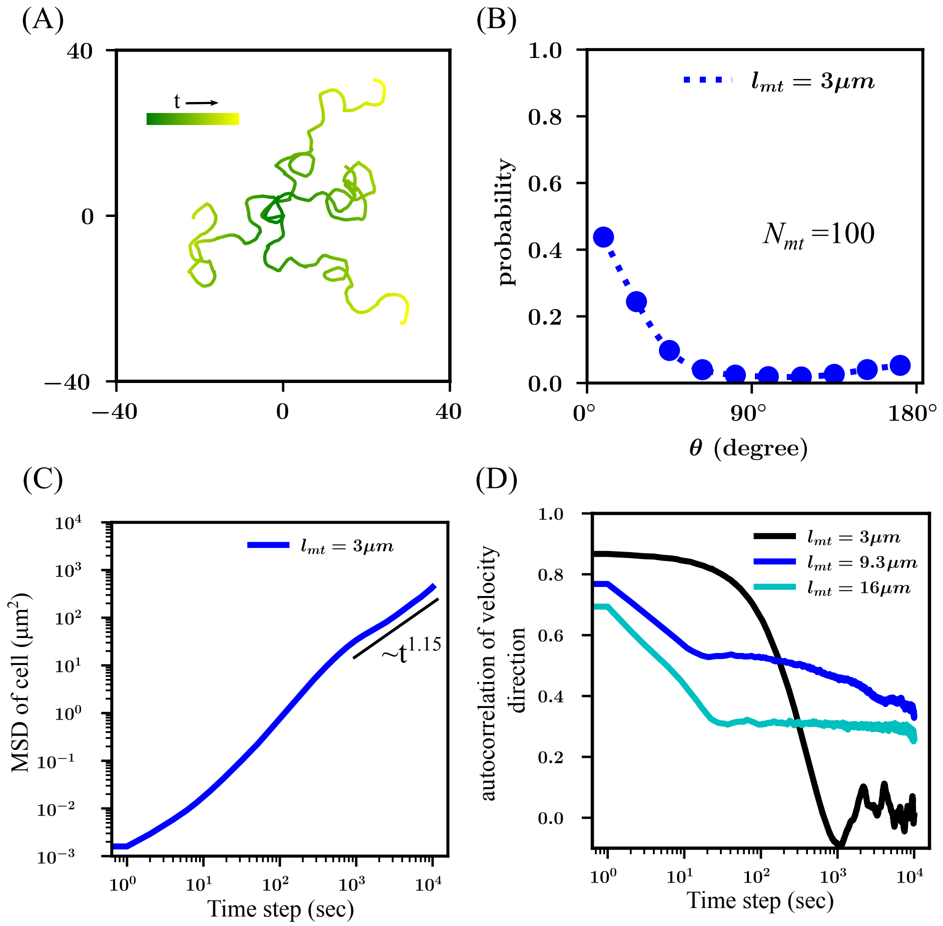

Figure S1. Short MTs frequently cause cells to change migration direction. (A) Trajectories of cell centroids with short average MT length of $l_{mt} = 3\mu m$ at an intermediate membrane stiffness of $k_{mem} = 3 \times 10^{-16}$J and MT stiffness of $k_{mt} = 5 \times 10^{-16}$J. **(B)** Probability distribution of angle between direction of cellular motion and nucleus to centrosome vector for $l_{mt} = 3\mu m$. **(C)** Mean square displacement of cells showing a $t^{1.15}$ dependence corresponding to super diffusive migration. **(D)** Velocity direction autocorrelation function shows velocity direction becomes uncorrelated with time for $l_{mt} = 3\mu m$.

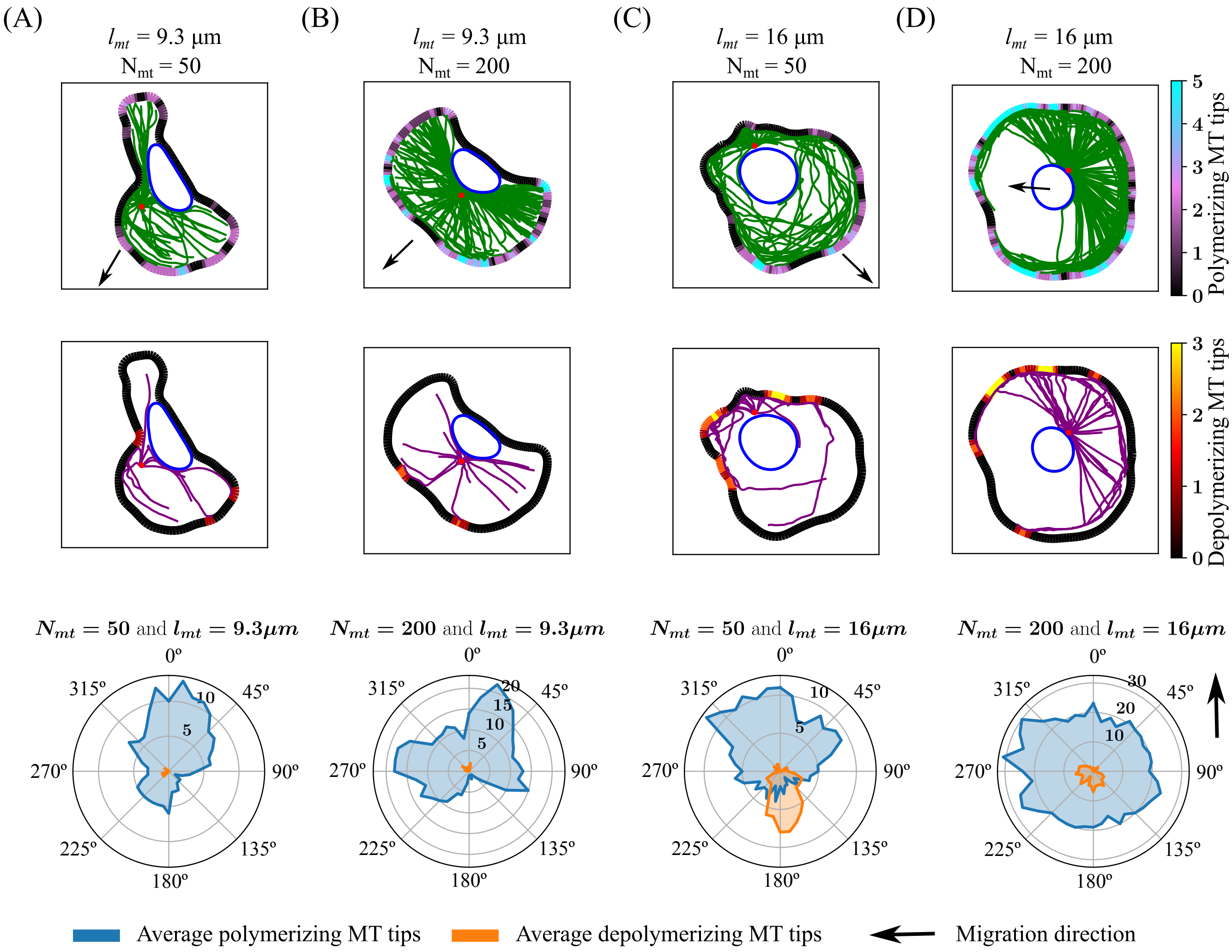

Figure S2. Distribution of growing and shrinking MT tips along the cell membrane : (A) Snapshots and distribution of growing and shrinking MT tips along the membrane for $N_{mt} = 50$ and $l_{mt} = 9.3\mu m$. **(B)** Snapshots and distribution of growing and shrinking MT tips along the membrane for $N_{mt} = 200$ and $l_{mt} = 9.3\mu m$. For regular MTs, the growing and shrinking MTs deposit polymerization and contraction signals mostly at the membrane region closer to the centrosome. **(C)** Snapshots and distribution of growing and shrinking MT tips along the membrane for $N_{mt} = 50$ and $l_{mt} = 16\mu m$. **(D)** Snapshots and distribution of growing and shrinking MT tips along the membrane for $N_{mt} = 200$ and $l_{mt} = 16\mu m$. For long MTs, the growing MT tips move to the region of the membrane away from the centrosome and shrinking MT tips are abundant along the membrane region closer to the centrosome.

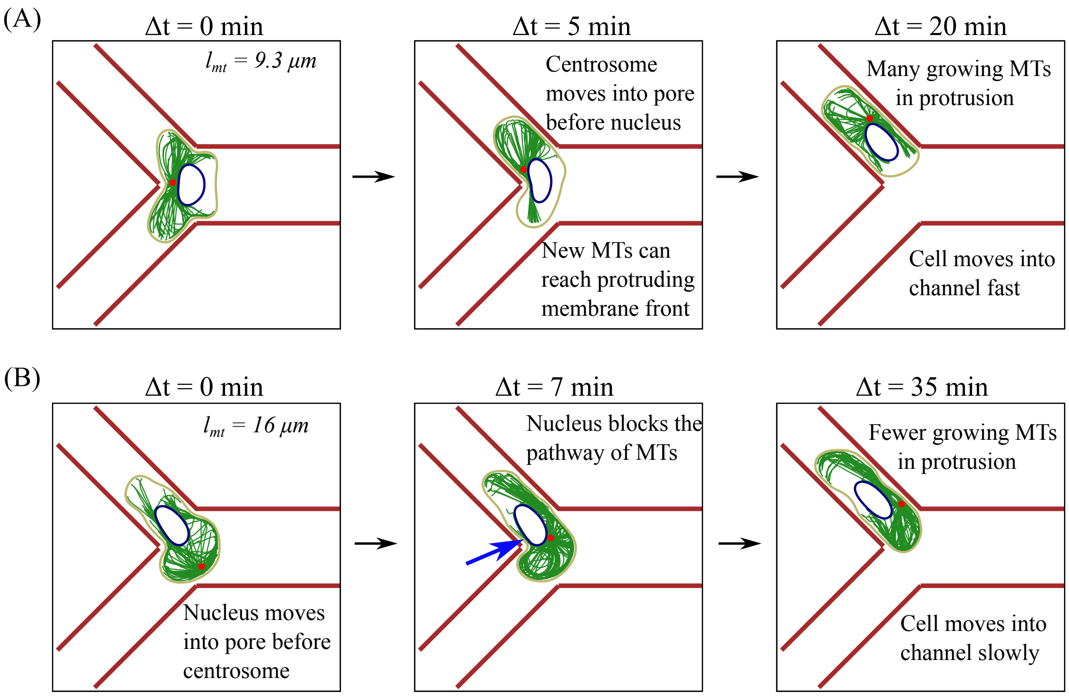

Figure S3. Migration of cell in Y channel : (A) Snapshots of cell moving into a symmetric Y channel with $l_{mt} = 9.3\mu m$. Centrosome is ahead of nucleus and new MT tips can reach protrusion tip without obstruction. (B) Snapshots of cell moving into a symmetric Y channel with $l_{mt} = 16\mu m$. Nucleus moves into the channel ahead of centrosome and obstructs new MT tips from reaching the protrusion tip.

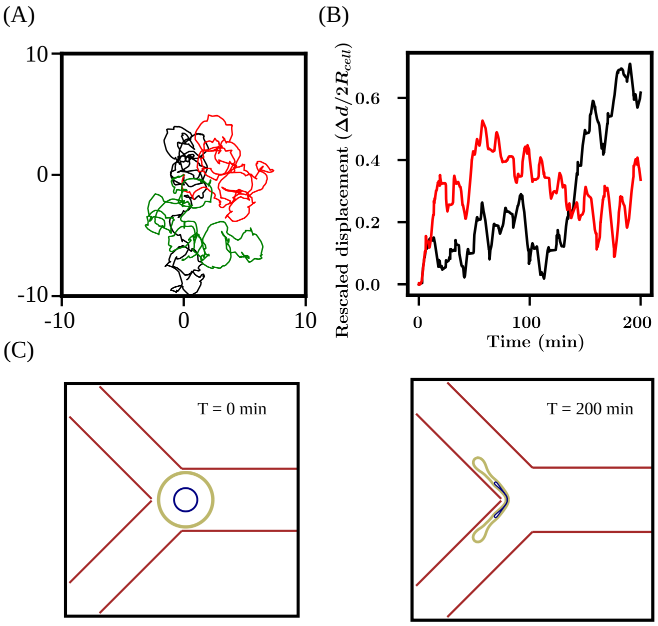

Figure S4. Very short or no MTs lead to migratory failure. (A) Typical trajectories of cell centroids with very short MTs ($l_{mt} = 1.0\ \mu m$) for $0 - 200\ min$. All trajectories start at the origin (0,0). **(B)** Cell centroid displacement from initial position scaled by $2R_{cell}$. **(C)** Initial and final configuration of cell without MTs at a Y junction channel. Loss of MTs leads to cell membrane collapse at Y junction.

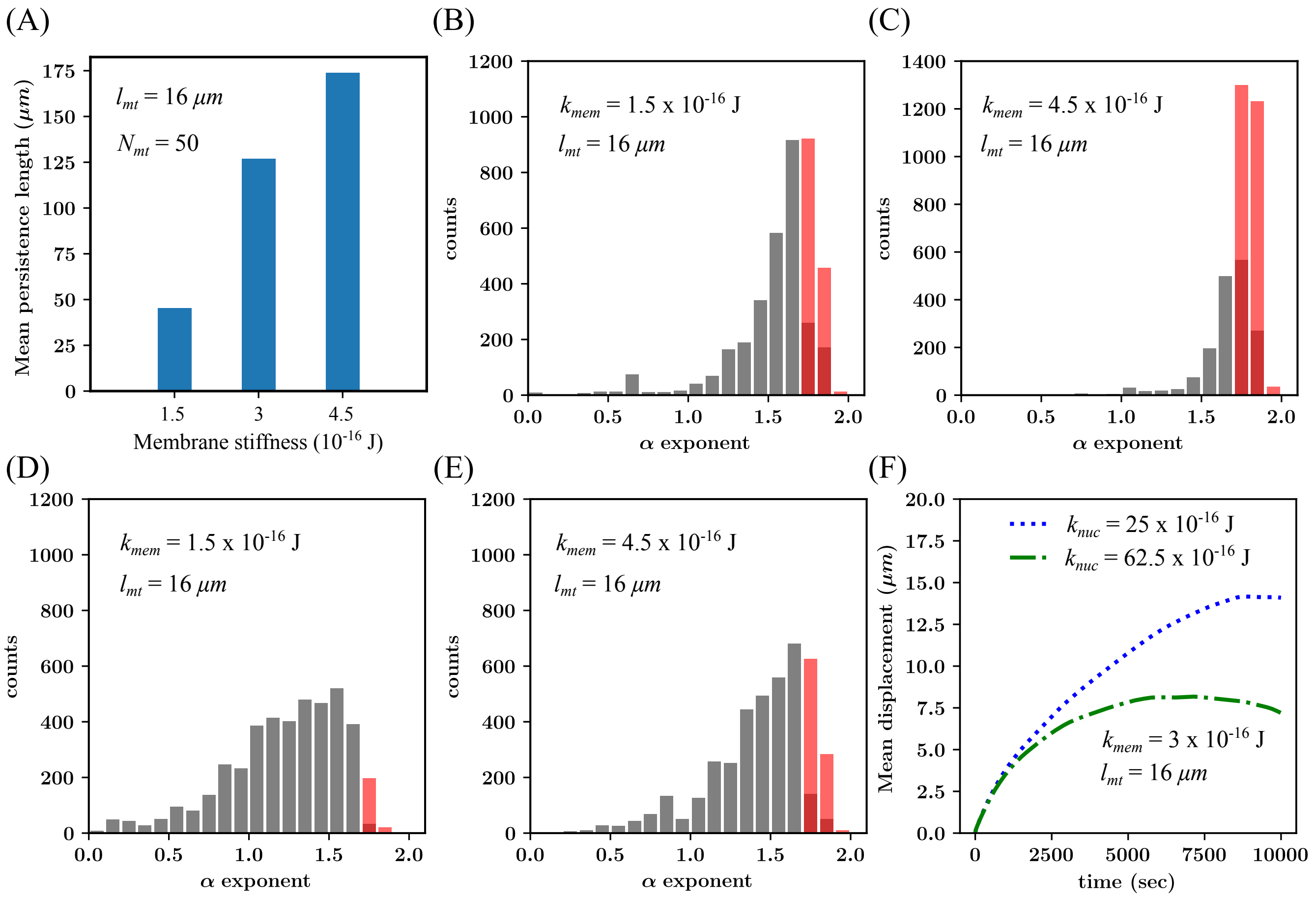

Figure S5. Membrane stiffness affects cell persistence. **(A)** Persistence length of cells with varying membrane stiffness for centrosome posterior to nucleus ($l_{mt} = 16\mu m$). **(B)** Distribution of local MSD exponent α for freely migrating cells with long MTs and $k_{mem} = 1.5 \times 10^{-16}$J. **(C)** Distribution of α for freely migrating cells with long MTs and $k_{mem} = 4.5 \times 10^{-16}$J. **(D)** Distribution of α for migrating cells in obstacle maze with long MTs and $k_{mem} = 1.5 \times 10^{-16}$J. **(E)** Distribution of α for migrating cells in obstacle maze with long MTs and $k_{mem} = 4.5 \times 10^{-16}$J. **(F)** Mean displacement of cells migrating in obstacle maze with long MTs and varying nucleus stiffness. All simulations were performed for $N_{mt} = 50$.

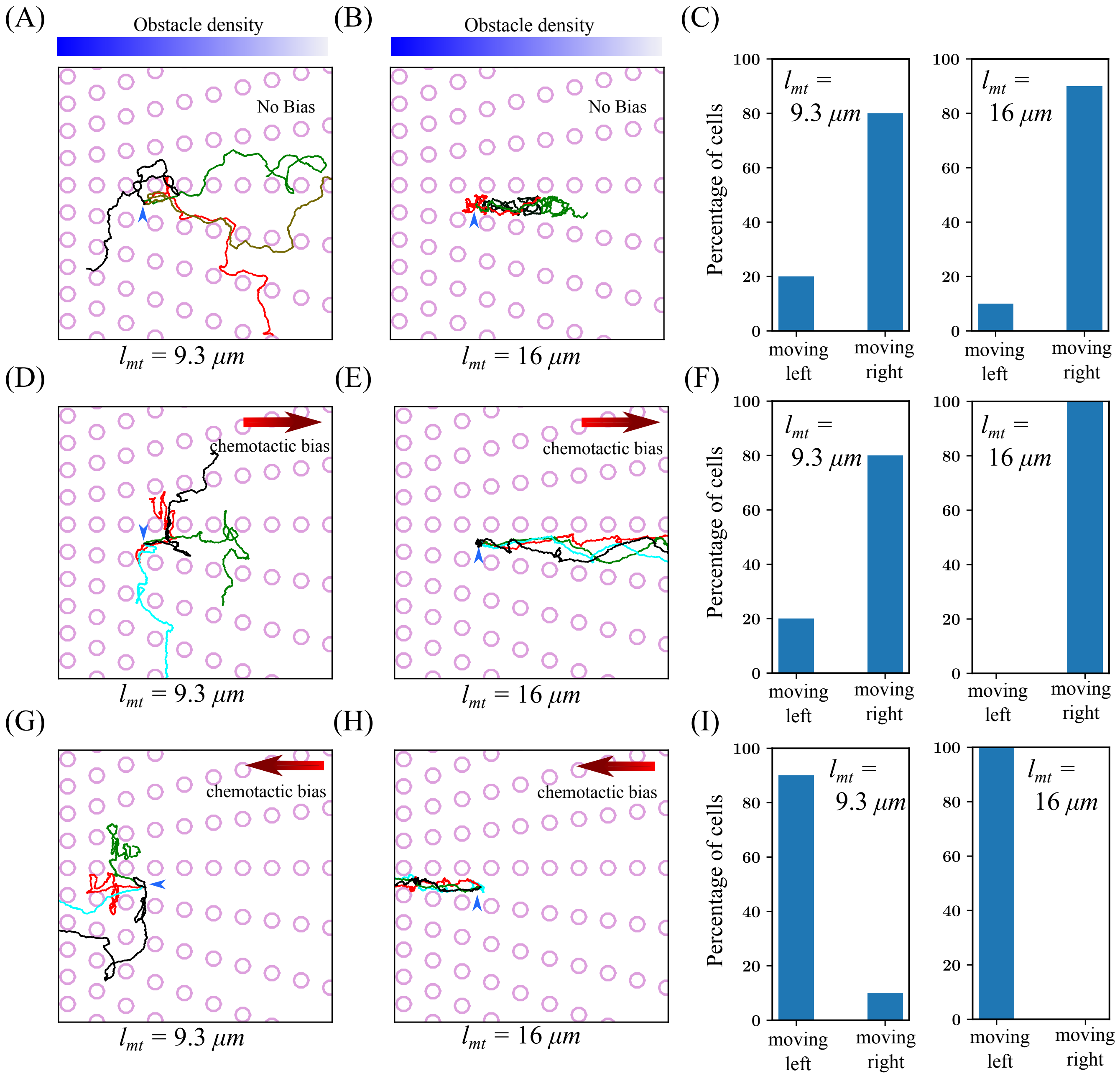

Figure S6. Cell migration in obstacle park with obstacle density gradient (A) Cell trajectories for cells with regular MTs and N_{mt} = 50, placed in an obstacle park with gradient obstacle density. **(B)** Cell trajectories for cells with long MTs in obstacle park with gradient obstacle density. **(C)** Percentage of cells moving left (towards higher obstacle density region) and cells moving right (lower obstacle density region) for regular and long MTs. **(D)** Cell trajectories for cells with regular MTs and N_{mt} = 50 in presence of a chemotactic bias towards the right (lower obstacle density region). **(E)** Cell trajectories for cells with long MTs and a chemotactic bias towards the right (lower obstacle density region). **(F)** Percentage of cells moving left and cells moving right for regular and long MTs in presence of chemotactic bias towards the right. **(G)** Cell trajectories for cells with regular MTs and a chemotactic bias along the left (higher obstacle density region). **(H)** Cell trajectories for cells with long MTs and a chemotactic bias along the left (higher obstacle density region). **(I)** Percentage of cells moving left and cells moving right for regular and long MTs, with a chemotactic bias towards the left (higher obstacle density region). Blue arrow indicates the initial position of the cell.

SUPPLEMENTAL TABLES

Symbol	Description	Value	Reference
N_m	Number of cell membrane beads	200	(1)
N_n	Number of nuclear membrane beads	200	(1)
N_{mt}	Number of MTs	50 - 200	(2–5)
N_{dyn}^m	Number of dynein motors on cell membrane	400	This study
N_{dyn}^n	Number of dynein motors on nuclear membrane	400,600	This study
R_{cell}	Initial radius of cell	7 μm	(6)
R_n	Initial radius of nucleus	3 μm	(7)
v_g	MT growth velocity	0.4 $\mu m s^{-1}$	(8, 9)
v_g	MT shrink velocity	0.8 $\mu m s^{-1}$	(9, 10)
f_c	MT catastrophe frequency	0.045-0.06285 s^{-1}	(10, 11)
f_r	MT rescue frequency	0.04 s^{-1}	(10, 11)
k_{mem}	Cell membrane stiffness	1.5-4.5 $\times 10^{-16}$J	(12, 13)
k_θ^m	Cell membrane bending rigidity	6.25 $\times 10^{-16}$J	(12)
l_0^m	Rest length of membrane spring attachments	0.22 μm	This study
ϵ_m	Strength of LJ interaction between membrane-nucleus	312.5 $\times 10^{-18}$J	This study
k_{nuc}	Nuclear membrane stiffness	$25 - 62.5 \times 10^{-16}$J	This study
k_θ^n	Nuclear membrane bending rigidity	3.125×10^{-16}J	This study
$2^{\frac{1}{6}}\sigma_m$	Cut-off distance for membrane-nucleus interaction	0.31μm	This study
k_{mt}	MT stretching stiffness	5 $\times 10^{-16}$J	(14)
k_θ^{mt}	MT bending rigidity	0.28 $\times 10^{-18}$J	(14)
l_0^{mt}	Rest length of MT spring attachments	0.2 μm	(15)
ϵ_{mt}	Strength of LJ interaction between MT-Cell/Nucleus	312.5 $\times 10^{-18}$J	This study
$2^{\frac{1}{6}}\sigma_{mt}$	Cut-off distance for MT-Cell/Nucleus LJ interaction	0.42μm	This study
k_d	Dynein spring attachment strength	0.125 $\times 10^{-18}$J	This study
v_{dyn}	Dynein walking speed on MTs	40nms^{-1}	(16)
d_{cut}	Dynein attachment cut-off distance	1μm	(15)
P_{dyn}	Dynein attachment probability	0.5	This study
P_{turn}	Dynein turnover probability	0.5	This study
k_c	Nucleus to cell membrane linear coupling strength	0.3125$\times 10^{-18}$J	This study
$l_p S_0 r_0$	Actin polymerization velocity in absence of polymerization signals	0.1$\mu m s^{-1}$	This study
C_p	Actin polymerization velocity increment per growing MT tip	0.1$\mu m s^{-1}$	This study
F_0	Cortical acto-myosin contraction force in absence of contraction signals	40-70 pN	This study
C_d	Cortical contraction force increment per shrinking MT tip	70 pN	This study
ζ_c	Effective coefficient of viscosity of cell membrane beads	6.25$\times 10^{-4} Nm^{-1} sec$	This study
ζ_n	Effective coefficient of viscosity of nucleus membrane beads	8$\times 10^{-4} Nm^{-1} sec$	This study
ζ_{mt}	Effective coefficient of viscosity of MT beads	1.5$\times 10^{-4} Nm^{-1} sec$	This study
ζ_{mt}	Effective coefficient of viscosity of centrosome	3$\times 10^{-4} Nm^{-1} sec$	This study

Table S1: **Parameter values used in simulations.** The characteristic length scale was chosen as $l_0 = 0.283\mu m$ to evaluate the spring constant energies ($k_{energy} = \frac{1}{2} k l_0^2$).

SUPPORTING MOVIES

Movies depicting cell migration in free and restricted environments.

Video S1 Persistent cell migration for $N_{mt} = 100$ and $l_{mt} = 9.3\mu m$; Persistent cell migration for $N_{mt} = 50$ and $l_{mt} = 16\mu m$.

Video S2 Diffusive cell migration for $N_{mt} = 100$ and $l_{mt} = 3\mu m$; Diffusive cell migration for $N_{mt} = 200$ and $l_{mt} = 16\mu m$.

Video S3 Cell migration in an obstacle park with obstacle spacing $d_{obs} = 20\mu m$ and obstacle radius $R_{obs} = 4\mu m$.

Video S4 Cell migration in an obstacle park with obstacle spacing $d_{obs} = 20\mu m$ and obstacle radius $R_{obs} = 5\mu m$.

Video S5 Cell migration in path choice device with 4 channels of width $2\mu m$, $4\mu m$, $6\mu m$, $8\mu m$.

SUPPLEMENTAL MATERIALS AND METHODS

Short MTs lead to loss of persistence

To test the effect of shortened MTs on cell polarization, cells were simulated with a short average MT length of $l_{mt} = 3\mu m$. The cell centroid trajectories revealed that the cell became increasingly prone to turning, leading to a loss of persistence (Fig. **S1 A**; see **Video S2**). The centrosome stayed ahead of the nucleus in the direction of migration (Fig. **S1 B**). The mean squared displacement of the cell centroid showed a scaling of 1.15, which corresponds to a super-diffusive regime (Fig. **S1 C**). Analysis of the velocity direction autocorrelation function indicated that cell migration direction was correlated at small time scales (Fig. **S1 D**). With increasing time, the correlation of cell velocity decayed steeply to zero, indicating that the cell was prone to changing the direction of propagation with time.

Distribution of MT signals at the membrane-cortex region

Within a cell containing regular MTs of length $l_{\mathrm{mt}} = 9.3\ \mu\mathrm{m}$, the membrane region near the centrosome receives the majority of both growing and shrinking MT tips (see Fig. **S2 A** and **S2 B**). Growing MT tips promote actin polymerization, leading to membrane protrusions, while shrinking tips transmit signals for actomyosin contraction. Because shrinking tips tend to retract from the membrane due to depolymerization, their numbers along the membrane are comparatively lower. This asymmetry leads to a higher concentration of actin polymerization activity near the centrosome, thereby establishing cell polarity. Under these conditions, the cell migrates with the centrosome positioned ahead of the nucleus. Increasing the number of MTs from $N_{\mathrm{mt}} = 50$ to $N_{\mathrm{mt}} = 200$ enhances the number of polymerizing MT tips near the centrosome. However, the number of shrinking tips does not increase significantly, as they continue to recede from the membrane due to depolymerization (compare Fig. **S2 A** and **S2 B**). As a result, cell polarization becomes more pronounced with a higher number of MTs for regular-length MTs.

For long MTs ($l_{\mathrm{mt}} = 16\ \mu\mathrm{m}$) and an intermediate MT count ($N_{\mathrm{mt}} = 50$–100), growing MT tips are predominantly located in membrane regions away from the centrosome (see Fig. **S2 C**), while shrinking tips are more concentrated near the centrosome. This distribution establishes a polarity in which actin polymerization is stronger away from the centrosome, and contractile activity is higher closer to it. Under these conditions, the cell migrates with the centrosome positioned behind the nucleus and exhibits a high persistence length. When the number of MTs is increased to $N_{\mathrm{mt}} = 200$, the region near the centrosome also receives a considerable number of growing MT tips (see Fig. **S2 D**), leading to additional actin polymerization signals in that area. This results in a reduction of cell polarity and decreased migration persistence.

MT dynamics at Y junction dictates channel selection time

When a cell with regular MTs reaches a Y-junction, the centrosome is generally positioned between the leading edge and the nucleus (see Fig. **S3 A**). Microtubules emanating from the centrosome can enter protrusions in either branch of the junction without obstruction. The winning protrusion is selected stochastically: more microtubules move into the selected protrusion, while the other loses microtubules and is subsequently retracted. As the cell advances into the selected branch, the centrosome remains ahead of the nucleus, allowing newly growing microtubules to readily reach the tip of the protrusion and deliver actin polymerization signals. This results in rapid cell passage through the chosen channel.

In cells with long MTs at a Y-junction, the centrosome is positioned behind the nucleus. Growing MTs must slide past the nucleus to reach the protrusion tips. One protrusion is selected stochastically, and the nucleus moves into the selected protrusion ahead of the centrosome (see Fig. **S3 B**). The nucleus then acts as a barrier for newly growing MTs attempting to reach the protrusion tip. As a result, the cell takes more time to fully enter the selected channel (see Fig. **6 C**).

Severely short MTs impair cell migration

The effect of MT depolymerization on the migration was investigated by simulating cells with average MT length severely shortened to $l_{mt} = 1.0\mu m$. Our results indicated that the cell failed to establish and maintain any front-back polarization with severely short MTs. Trajectories of cell centroids in Fig. **S4 A-B** indicate that the cell centroid did not have a net displacement in the order of the cell diameter throughout the simulation time. We interpret this as an overall failure of the cell migration. Events of migration failure due to severely shortened MTs have been demonstrated in nocodazole-treated cells that depolymerize MTs (17).

The functional consequence of complete MT depolymerization for cells migrating in restricted geometries was checked by simulating cells without MTs at a Y junction. A small velocity was given to each cell membrane bead directed inwards into the channel, depending on which channel the bead was in. Cells at a symmetric Y junction (channel width = $10\mu m$) failed to migrate into either channel. The cell collapsed at the Y junction in the absence of MTs (Fig. **S4 C**).

Stiffer cell membrane-actin cortex and softer nucleus improves persistence in posterior centrosome configuration

The influence of the cell membrane and underlying actin cortex stiffness on the migration of the cell was investigated by varying the membrane spring constant k_{mem} in the simulations. For cells with regular MTs ($l_{mt} = 9\mu m$), no significant change in migration characteristics were observed. However, cells with long MTs ($l_{mt} = 16\mu m$) were found to migrate more persistently in unrestricted geometries with a stiffer cell membrane (Fig. **S5 A**). Analysis of the local msd exponent α revealed that, in the centrosome posterior to nucleus configuration, a stiff cell membrane leads to more directed run phases of the cell (Fig. **S5 B, C**). A stiffer cell membrane and actin cortex help in faster retraction of the cell rear through enhanced membrane tension and result in improved directed locomotion of the cell. In obstacle parks, cells with long MTs ($l_{mt} = 16\mu m$) were found to show improved migration with a stiffer cell membrane and underlying actin cortex. This was indicated by the presence of higher counts of directed run phases for $k_{mem} = 4.5 \times 10^{-16}$J as compared with $k_{mem} = 1.5 \times 10^{-16}$J (Fig. **S5 D, E**). Finally, a softer nucleus was found to improve the capability of the cells to squeeze through narrow pores between obstacles in obstacle parks. For obstacle radius $R_{obs} = 5\mu m$ and spacing $\Delta d = 20\mu m$, cells having long MTs with $k_{mem} = 3 \times 10^{-16}$J and $k_{nuc} = 62.5 \times 10^{-16}$J found it difficult to move through the narrow pores between obstacles resulting in stuck configurations of the cell. However, when the nucleus membrane stiffness was reduced to $k_{nuc} = 25 \times 10^{-16}$J the ability of the cells to migrate through the narrow spacings between obstacles improved, resulting in increased average displacement of the cell centroid with time (Fig. **S5 F**).

Unbiased cells move towards less dense regions in obstacle parks with varying density

Finally, we investigated the behavior of migrating cells within obstacle parks with varying obstacle densities. Cells with regular and long MTs mostly moved towards the region with a sparse distribution of obstacles, with few cells moving toward the region densely packed with obstacles (Fig. **S6 A-C**). This suggested that cells can use their MT-actin crosstalk to navigate towards less restrictive regions. For cells with regular MTs, a greater percentage ($\approx 20\%$) of cells moved towards the denser region as compared with cells with long MTs ($\approx 10\%$). However, cells with regular microtubules were able to move more robustly within the obstacle park, as was also seen for regularly spaced obstacle parks (see Fig. **4**).

Next, we introduced a chemotactic bias on the cell by adding a small velocity to the cell membrane beads in the direction of the bias. The migration of cells with regular MTs was found to be hampered when the chemotactic bias was in the direction of sparsely placed obstacles (Fig. **S6 D**). This indicated that the polarization of the cell due to microtubule-delivered signals failed to align with the chemotactic bias. Therefore, the cell demonstrated a reduction in its ability to navigate through the narrow spaces between obstacles. However, most cells moved towards the sparse obstacle density region. For long MTs, all the cells moved towards the sparse obstacle density region with the migration becoming more robust (Fig. **S6 E, F**).
The direction of the chemotactic bias was then reversed towards the densely packed region. Cells with regular microtubules mostly moved towards the densely packed region (Fig. **S6 G**). A small percentage ($\approx 10\%$) of cells moved towards the sparsely packed region opposite to the applied chemotactic gradient. Cells with long MTs only moved towards the densely packed region, indicating that long MT cells can align themselves better to the applied chemotactic gradient (Fig. **S6 H, I**).

DETAILS OF THE MODEL

A mechanistic model of cell migration primarily involves membrane protrusion and retraction, which are coupled to the actomyosin cortex and actin cytoskeleton. This process includes actin polymerization, myosin-driven membrane contraction, polarization of actin filaments, focal adhesion kinetics, and membrane surface tension. Research has shown that MT

depolymerization regulates actomyosin contraction through the modulation of Rho GTPase signaling pathways (18). On the other hand, MT polymerization can regulate actin polymerization and the expansion of protrusions by transporting intracellular cargo and signaling molecules to the leading edge of migrating cells (18–20). We use these two observations as key components to develop a mechanistic whole-cell model of MT-actin crosstalk during cell migration.

We focus on two-dimensional mesenchymal cell migration, using the following basic model elements: 1) bead-spring loops to represent the semi-flexible boundary of the cell and its (circular) nucleus, 2) bead-spring semi-flexible polymers for dynamic MTs (MTs), which can grow and shrink at their plus ends through the addition and removal of beads, respectively. These MTs are anchored at the MT organizing center (MTOC), which we assume to coincide with the cell's centrosome, and 3) growing MTs exert pushing forces on the membrane when they come into contact, while dyneins, anchored at the cell and nucleus membranes, can attach to MTs and generate pulling forces on them. A sketch of these standard parts of our model (c.f. (21, 22)) is shown in Fig. **1**). As new model elements, we incorporate MT-actin crosstalk by introducing a regulatory cue that modulates increased actin polymerization at the cortex or membrane contraction due to myosin-II activity, which is locally delivered to the cortex at the MT tips. Shrinking MTs deliver cues for myosin activity, leading to local contraction, while growing MTs deliver cues for actin polymerization, resulting in local expansion. Instead of modeling the actin network explicitly, we represent local contraction and expansion through their effective action on the individual beads that represent the cell boundary. First, actin-MT crosstalk due to shrinking MTs leads to myosin-generated stress in the actin cortex, causing membrane retraction via the RhoA GEF LFc signaling pathway (18). We model this event chain by introducing an effective inward force on a membrane bead that is close to the tip of a shrinking MT. The net inward force on a membrane bead is proportional to the number of shrinking MT tips in the vicinity of the membrane bead (see Fig. **1**). Second, actin-MT crosstalk due to growing MTs leads to the formation of membrane protrusions or lamellipodia due to actin polymerization against a membrane. This induces a retrograde actin flow toward the nucleus, which is opposed by focal adhesions connecting actin filaments across the membrane to the substrate. To describe this sequence of events, we examine a model of lamellipodial protrusion guiding confined cell migration (23). In this model, the local actin polymerization force acting on a segment of the cell membrane is linked to both the actin polymerization rate and the average orientation of actin filaments near the membrane (23–26). Consequently, the actin polymerization force generates an outward-directed velocity component on the membrane beads, which is influenced by the polymerization signals transmitted from the growing microtubule (MT) tips to the actin cortex (c.f. Fig. **1**). To incorporate actin-MT crosstalk, we assume that the outward velocity imparted to the membrane bead along the outward normal is proportional to the number of growing MT tips near the membrane bead. Additionally, myosin-generated contraction forces, mediated by actin filaments between the protrusion's leading edge and the nucleus, counteract the expansion of the protrusion, resulting in an effective elastic coupling between the position of the nucleus and the protrusion edge (23). We model this elastic coupling by linking the beads of the nucleus boundary to those of the cell boundary with elastic springs, as illustrated in Fig. **1**.

Membrane dynamics

The cell and nuclear membrane are modeled as a closed chain of N_{mem} bead spring units (see Fig. **1 A**) (27, 28). Each bead is connected to two neighboring beads on either side via a spring, and an angular potential is considered between the angle formed by every three adjacent beads. The nuclear membrane beads also have a Lennard-Jones interaction with the cell membrane beads within a cut-off distance such that the interaction forces are always repulsive.

$$U^m_{sp} = \frac{1}{2}k_{mem}(l^m - l^m_0)^2 \tag{1}$$

$$U^m_\theta = \frac{1}{2}k^m_\theta(1 - \cos(\theta - \theta_0)) \tag{2}$$

$$U_{m-n} = 4\epsilon_m\left[\left(\frac{\sigma_m}{r_{m-n}}\right)^{12} - \left(\frac{\sigma_m}{r_{m-n}}\right)^6\right] \tag{3}$$

Here, l^m denotes the stretched length of the spring joining two adjacent membrane beads, l^m_0 denotes the rest length of the spring joining two adjacent membrane beads, and r_{m-n} denotes the distance between a membrane bead and nuclear membrane bead. The equation of motion for a cell or nuclear membrane bead then follows as,

$$\dot{\vec{r}}^{c/n}_i = \frac{\vec{F}_{pot}}{\zeta_{c/n}} + \sqrt{\frac{2k_bT}{\zeta_{c/n}}}\eta(t) \tag{4}$$

$\vec{F}_{pot}$ represents the sum total of all forces on the beads due to the potentials considered in Eq. 1-3. $\zeta_{c/n}$ is the coefficient of viscosity of the membrane/nucleus beads and $\eta(t)$ is a Gaussian noise with zero mean and $\langle\eta(t)\eta(t')\rangle = \delta(t - t')$.

MT dynamics

MTs are modeled as open-ended bead spring units with their minus ends anchored at the centrosome (14). The MT beads are connected to adjacent beads on either side via a spring, and an angular potential is considered between the angle formed between three adjacent beads in an MT. The dynamic instability of the MT is incorporated into the model by adding (or removing) beads at the plus end of a growing (or shrinking) MT at intervals of t_{mt} timesteps (see Fig. **1 B**). New beads are added to growing MTs in a given time step only if the new bead falls within the cell and outside the nucleus. The dynamic instability of MTs is governed by four parameters, namely, growth velocity (v_g), shrink velocity (v_s), catastrophe frequency (f_c), and rescue frequency (f_r) (10, 29). An increase in catastrophe frequency (f_c), or a decrease in rescue frequency (f_r), reduces the average MT length. Similarly, an increase in rescue frequency (f_r) or a decrease in catastrophe frequency (f_c) increases the average MT length in the cell.
The membrane beads and MT beads have Lennard-Jones interaction between them within a cut-off distance such that the interaction forces are always repulsive.

$$U_{sp}^{mt} = \frac{1}{2}k_{mt}(l^{mt} - l_0^{mt})^2 \tag{5}$$

$$U_{\theta}^{mt} = \frac{1}{2}k_{\theta}^{mt}(1 - \cos(\theta - \theta_0)) \tag{6}$$

$$U_{mt-m/n} = 4\epsilon_{mt}\left[\left(\frac{\sigma_{mt}}{r_{mt-m/n}}\right)^{12} - \left(\frac{\sigma_{mt}}{r_{mt-m/n}}\right)^{6}\right] \tag{7}$$

Here, l^{mt} denotes the stretched length of the spring joining two adjacent MT beads, l_0^{mt} denotes the rest length of the spring joining two adjacent MT beads, $r_{mt-m/n}$ denotes the distance between a MT bead and a membrane bead (or a nuclear membrane bead). The equation of motion for an MT bead reads,

$$\dot{\vec{r}}_i^{mt} = \frac{\vec{F}_{pot}^{mt}}{\zeta_{mt}} + \sqrt{\frac{2k_bT}{\zeta_{mt}}}\eta(t) \tag{8}$$

The parameters chosen for microtubule dynamics were such that microtubule polymerization did not lead to the formation of stable membrane protrusions.

Dynein dynamics

Dynein motors are placed randomly on membrane beads (or nuclear membrane beads) and scanned for MT beads near them. Upon encountering MT beads within a cut-off distance d_{cut}, dynein motors form spring-like bonds with them. After each time step, the dynein motors can shift to the adjoining MT bead toward the negative end of the MT if the adjoining bead is within the cutoff radius d_{cut}. The dynein walking speed on the MTs is given by v_{dyn}. Dynein pulling forces are modeled as simple spring forces between the membrane (or nuclear membrane) beads and MT beads.

$$U_d = \frac{1}{2}k_d d^2 \tag{9}$$

Where d is the distance between the centers of the membrane (or nuclear membrane) bead and the MT bead.

Actin dynamics

We modeled the effects of actin network dynamics as protrusive and contractile forces on the membrane beads. The protrusive forces caused by actin polymerization are considered to add a component to the velocity of the membrane beads directed in the outward normal direction (23). This outward component of the velocity depends on the average local orientation S_{act} of the actin filaments in the vicinity of the membrane bead and the local actin polymerization rate r_p. The net outward velocity added to the membrane beads is then $l_a S_{act} r_p$, where l_a is the diameter of individual actin filaments. Mechanotransduction of forces from the cell membrane to the nuclear membrane is known to happen due to the interconnection of actin filaments and intermediate filaments with the nuclear membrane (30, 31). Myosin motors walk on counter-oriented actin filaments in the crossover region between the cell membrane and the nuclear membrane to generate a contraction force (32). An increase in actin polymerization activity at the cell front increases the retrograde flow of actin. This results in more active myosin motors in the crossover region between the protrusion tip and the nucleus, leading to an increase in contraction forces. The strength of this

contractile force depends on the actin retrograde flow velocity v_i^r at the protruding membrane bead. To evaluate the net velocity change of the protruding membrane beads due to cortical actin activity, we follow (23), and equate the net change in bead velocity along the outward direction to the difference between the outward actin polymerization velocity and inward retrograde flow velocity.

$$\dot{r}_i^c = l_a S_{act} r_p - v_i^r \tag{10}$$

The retrograde flow velocity can be assumed to be driven by the membrane tension f_τ and the contraction force f_c at the membrane due to coupling with the nucleus through actin filaments and myosin motors. It can be shown that f_c and f_τ vary linearly with the length of the protrusion (23, 25, 26, 33).

$$\vec{v}_i^r = -\frac{k_c}{\zeta_c}(\vec{r}_i^c - \vec{r}_i^n) \tag{11}$$

where k_c represents the strength of the uniform linear coupling between the cell membrane beads and nuclear membrane beads. The protrusion length is the distance $(\vec{r}_i^c - \vec{r}_i^n)$ between the cell membrane bead and the nuclear membrane bead. The cell membrane attaches to the substrate through the formation of focal adhesions. The formation and dissolution of focal adhesions modulate the interaction between the membrane. This interaction is included in our model as an effective viscous drag on the membrane beads whose strength is given by the coefficient ζ_c (23, 34). We also consider a local contractile force F_c due to myosin activity in the cell cortex along the cell membrane that does not couple with the nucleus, directed opposite to the outward normal at the membrane. The final equation of motion for the membrane beads then reads as follows:

$$\dot{\vec{r}}_i^c = -\frac{k_c}{\zeta_c}(\vec{r}_i^c - \vec{r}_i^n) + l_p S_{act} r_p \hat{n}_i - \frac{F_c}{\zeta_c}\hat{n}_i + \frac{\vec{F_{pot}}}{\zeta_c} + \sqrt{\frac{2k_bT}{\zeta_c}}\eta(t) \tag{12}$$

$\hat{n}_i$ is a unit vector pointing along the outward normal along the membrane bead. F_{pot} is the sum of all the forces due to the interaction potentials between the membrane and the nucleus and MT beads. We assume that the values of S_{act} and r_p depend on actin polymerization cues that are supplied to the region near the membrane beads by polymerizing MTs and their values are calculated according to,

$$l_p S_{act} r_p = l_p S_0 r_0 + C_p n_{mt}^{pol} \tag{13}$$

Where $l_p S_0 r_0$ is the component of the outward polarization velocity in the absence of polarization cues delivered by the growing MT tip, n_{mt}^{pol} is the number of polymerizing MTs in the vicinity of the membrane bead and C_p is the component of protrusion velocity added per polymerizing MT. The strength of the contraction force F_c, due to myosin activity at the cortex, is considered to depend on contraction cues provided by depolymerizing MT tips near the membrane beads and varies according to :

$$F_c = F_0 + C_d n_{mt}^d \tag{14}$$

Where F_0 is the strength of the contraction force in the absence of any MT-supplied contraction cue. C_d is the net contraction force added per depolymerizing MT present near the membrane bead, and n_{mt}^d is the number of depolymerizing MTs near the membrane bead. The distance of the membrane bead from a MT tip, within which the dynamics of actin polymerization or acto-myosin contraction at the cortex can be influenced by the MT tip delivered signal, was chosen as $2 \times 1.12\sigma_{mt}$. Finally, the equation of motion of the nucleus beads reads,

$$\dot{\vec{r}}_i^n = -\frac{k_c}{\zeta_n}(\vec{r}_i^n - \vec{r}_i^c) + \frac{F_{pot}^n}{\zeta_n} + \sqrt{\frac{2k_bT}{\zeta_n}}\eta(t) \tag{15}$$

Where F_{pot}^n represents the sum of all the forces on the nucleus bead due to the steric and dynein interactions considered in our model.

SIMULATION METHOD

All simulations were initialized with a circular cell and nucleus and the centrosome was placed randomly within the cell. All MTs were initialized to be growing and their directions of growth were chosen randomly. The simulation time step was taken to be $0.1sec$. The beads were added or removed from the MT tips after each N_{pol} time step according to their growth or shrinkage state. The probabilities for MT catastrophe or rescue were calculated after every N_{pol} time steps and the MT state was updated

accordingly. N_{pol} was taken as 10 timesteps. The number of growing or shrinking MT tips near a membrane bead was checked at every time step and actin polymerization or cortical contraction signal strengths were calculated. Dynein motors were placed on the cell membrane and nuclear membrane beads randomly, and positions of attachments were searched on nearby MTs at every time step. Upon finding possible sites of attachments, dynein motor bonds were established between the membrane and MT beads with a certain probability P_{dyn}. Established dynein bonds were checked for turnover and bonds were detached according to the turnover probability P_{turn} at every time step. For the force dynamics, we considered a relaxation timestep of $0.001 sec$. Forces on every component in the model were calculated at every relaxation time step and their positions were updated accordingly.

The codes were developed in C using OpenMP. The data analysis and plots were done in Python and Gnuplot. The computational time for a single simulation running on 20 processors (Intel Xeon CPU, having a clock speed of 2 GHz and RAM 64 GB) was in the range of 10-20 hours.

DATA ANALYSIS

Persistence length

The local directional persistence, or the ability of the cell to maintain its direction of motion, is quantified as $p = cos\theta$ with θ being the angle between the instantaneous velocity directions at two time steps. The persistence length l_p can then be calculated from $p = e^{-\frac{d}{l_p}}$, where d is the displacement of the cell between the two time steps. The local persistence length l_p was evaluated at consecutive time steps throughout the motion of the cell and its average was calculated as the mean persistence length $\langle l_p \rangle$ (35).

Local mean square displacement and velocity standard deviation analysis of cell trajectories

Local mean square displacements were evaluated by considering a rolling time window of $N_t = 30$ points at consecutive time steps 300 seconds apart. At every time step t_i, the local mean square displacement ΔR_i^2 was evaluated as a function of the time lag $\tau_m = m\delta t$ as (36),

$$\Delta R_i^2(t_i, \tau_m) = \frac{1}{N_t - m + 1} \sum_{j=-\frac{N_t}{2}}^{j=\frac{N_t}{2}-m} \Big[\mathbf{R}(t_{i+j} + m\delta t) - \mathbf{R}(t_{i+j})\Big]^2 \tag{16}$$

where δt is the time between two rolling window points and $\mathbf{R}_i = (X_i, Y_i)$ are the coordinates of the cell centroid. The total duration of the rolling window is $T = N_t \delta t$.
The standard deviation of the velocity was calculated at consecutive time steps from the values of the velocity direction $\phi_i(t_i)$ as (36),

$$\Delta\phi(t_i) = \left[\frac{1}{N_t - m + 1} \sum_{j=-\frac{N_t}{2}}^{j=\frac{N_t}{2}-m} (\phi(t_{i+j} + m\delta t) - \phi(t_{i+j}))^2\right]^{\frac{1}{2}} \tag{17}$$

the value of m was chosen such that $m\delta t = T/4$.

The local mean square displacement ΔR_i^2 was assumed to scale with the time lag τ_m as,

$$\Delta R_i^2 = A\tau_m^{\alpha} \tag{18}$$

For $\alpha = 1$, the motion of the cell is completely diffusive, and for $\alpha = 2$, the motion of the cell is ballistic. We considered the motion of the cell in two separate phases. For $\alpha > 1.7$ and $\Delta\phi < 0.9$, the cell migration was considered to be in the directed motion phase; otherwise, the cell motion was considered to be in the random migration phase.

Evaluation of cell polarization by MT signals

To evaluate the distribution of MT tips along the membrane in Fig. **S2 B**, we counted the number of MT tips associated with membrane beads. Average MT numbers along the cell membrane were computed by identifying MT tips located near a

membrane bead, such that the bead lies within the range where polymerization and contraction signals can be transmitted. A rolling average was then applied over 60 consecutive snapshots spanning a 60-second interval. The data were plotted based on the angular position of each membrane bead relative to the direction of migration.

SUPPORTING REFERENCES

1. Lee, S. Y., P. W. A. Schönhöfer, and S. C. Glotzer, 2023. Complex motion of steerable vesicular robots filled with active colloidal rods. *Scientific Reports* 13:22773.

2. Koonce, M. P., M. P. Koonce, A. Khodjakov, and A. Khodjakov, 2002. Dynamic microtubules in Dictyostelium. *Journal of Muscle Research and Cell Motility* 23:613–619.

3. Ryder, M. I., R. N. Weinreb, and R. Niederman, 1988. Microtubule-granule relationships in motile human polymorphonuclear leukocytes. *Anatomical Record-advances in Integrative Anatomy and Evolutionary Biology* 221:679–686.

4. Schliwa, M., K. B. Pryzwansky, and U. Euteneuer, 1982. Centrosome splitting in neutrophils: an unusual phenomenon related to cell activation and motility. *Cell* 31:705–717.

5. Schulze, E., and M. Kirschner, 1986. Microtubule dynamics in interphase cells. *The Journal of cell biology* 102:1020–1031.

6. Murphy, P., 1976. Morphology and Cellular Physiology of Neutrophil Granulocytes, Springer US, Boston, MA, 17–31.

7. Lammerding, J., 2011. Mechanics of the Nucleus, John Wiley & Sons, Ltd, 783–807.

8. van Haren, J., and T. Wittmann, 2019. Microtubule Plus End Dynamics "-" Do We Know How Microtubules Grow? *BioEssays* 41:1800194.

9. Zwetsloot, A. J., G. Tut, and A. Straube, 2018. Measuring microtubule dynamics. *Essays in Biochemistry* 62:725–735.

10. Kirschner, M., and T. Mitchison, 1986. Beyond self-assembly: From microtubules to morphogenesis. *Cell* 45:329–342.

11. Walker, R. A., E. T. O'Brien, N. K. Pryer, M. F. Soboeiro, W. A. Voter, H. P. Erickson, and E. D. Salmon, 1988. Dynamic instability of individual microtubules analyzed by video light microscopy: rate constants and transition frequencies. *Journal of Cell Biology* 107:1437–1448.

12. Vutukuri, H. R., M. Hoore, C. Abaurrea-Velasco, L. van Buren, A. Dutto, T. Auth, D. A. Fedosov, G. Gompper, and J. Vermant, 2020. Active particles induce large shape deformations in giant lipid vesicles. *Nature* 586:52–56.

13. Wang, C., Y.-k. Guo, W.-d. Tian, and K. Chen, 2019. Shape transformation and manipulation of a vesicle by active particles. *The Journal of Chemical Physics* 150:044907.

14. Soheilypour, M., M. Peyro, S. Peter, and M. Mofrad, 2015. Buckling Behavior of Individual and Bundled Microtubules. *Biophysical Journal* 108:1718–1726.

15. Oelz, D. B., U. del Castillo, V. I. Gelfand, and A. Mogilner, 2018. Microtubule Dynamics, Kinesin-1 Sliding, and Dynein Action Drive Growth of Cell Processes. *Biophysical Journal* 115:1614–1624.

16. Ezber, Y., V. Belyy, S. Can, and A. Yildiz, 2020. Dynein harnesses active fluctuations of microtubules for faster movement. *Nature Physics* 16:312–316.

17. Liao, G., T. Nagasaki, and G. G. Gundersen, 1995. Low concentrations of nocodazole interfere with fibroblast locomotion without significantly affecting microtubule level: implications for the role of dynamic microtubules in cell locomotion. *Journal of Cell Science* 108:3473–3483.

18. Kopf, A., J. Renkawitz, R. Hauschild, I. Girkontaite, K. Tedford, J. Merrin, O. Thorn-Seshold, D. Trauner, H. Häcker, K. D. Fischer, E. Kiermaier, and M. Sixt, 2020. Microtubules control cellular shape and coherence in amoeboid migrating cells. *Journal of Cell Biology* 219:e201907154.

19. Dogterom, M., and G. H. Koenderink, 2019. Actin–microtubule crosstalk in cell biology. *Nature Reviews Molecular Cell Biology* 20:38–54.

20. Henty-Ridilla, J. L., A. Rankova, J. A. Eskin, K. Kenny, and B. L. Goode, 2016. Accelerated actin filament polymerization from microtubule plus ends. *Science* 352:1004–1009.

21. Hornak, I., and H. Rieger, 2020. Stochastic Model of T Cell Repolarization during Target Elimination (I). *Biophysical Journal* 118:1733–1748.

22. Hornak, I., and H. Rieger, 2022. Stochastic model of T cell repolarization during target elimination (II). *Biophysical Journal* 121:1246–1265.

23. Flommersfeld, J., S. Stöberl, O. Shah, J. O. Rädler, and C. P. Broedersz, 2024. Geometry-Sensitive Protrusion Growth Directs Confined Cell Migration. *Phys. Rev. Lett.* 132:098401.

24. Lavi, I., M. Piel, A.-M. Lennon-Duménil, R. Voituriez, and N. S. Gov, 2016. Deterministic patterns in cell motility. *Nature Physics* 12:1146–1152.

25. Sens, P., 2020. Stick–slip model for actin-driven cell protrusions, cell polarization, and crawling. *Proceedings of the National Academy of Sciences* 117:24670–24678.

26. Ron, J. E., P. Monzo, N. C. Gauthier, R. Voituriez, and N. S. Gov, 2020. One-dimensional cell motility patterns. *Phys. Rev. Res.* 2:033237.

27. Abaurrea-Velasco, C., T. Auth, and G. Gompper, 2019. Vesicles with internal active filaments: self-organized propulsion controls shape, motility, and dynamical response. *New Journal of Physics* 21:123024.

28. Wang, H., Z. Jia, and Y. Fang, 2024. Chemo-mechanical model of cell polarization initiated by structural polarity. *Soft Matter* 20:8407–8419.

29. Dogterom, M., and S. Leibler, 1993. Physical Aspects of the Growth and Regulation of Microtubule Structures. *Physical Review Letters* 70(9):1347–1350.

30. Stöberl, S., J. Flommersfeld, M. M. Kreft, M. Benoit, C. P. Broedersz, and J. O. Rädler, 2024. Nuclear deformation and dynamics of migrating cells in 3D confinement reveal adaptation of pulling and pushing forces. *Science advances* 10:eadm9195.

31. Maniotis, A. J., C. S. Chen, and D. E. Ingber, 1997. Demonstration of mechanical connections between integrins, cytoskeletal filaments, and nucleoplasm that stabilize nuclear structure. *Proceedings of the National Academy of Sciences* 94:849–854.

32. Lou, S. S., A. Diz-Muñoz, O. D. Weiner, D. A. Fletcher, and J. A. Theriot, 2015. Myosin light chain kinase regulates cell polarization independently of membrane tension or Rho kinase. *Journal of Cell Biology* 209:275–288.

33. Brückner, D. B., M. Schmitt, A. Fink, G. Ladurner, J. Flommersfeld, N. Arlt, E. Hannezo, J. O. Rädler, and C. P. Broedersz, 2022. Geometry Adaptation of Protrusion and Polarity Dynamics in Confined Cell Migration. *Phys. Rev. X* 12:031041.

34. Kim, D.-H., and D. Wirtz, 2013. Focal adhesion size uniquely predicts cell migration. *The FASEB Journal* 27:1351–1361.

35. Shaebani, M. R., R. Jose, L. Santen, L. Stankevicins, and F. Lautenschläger, 2020. Persistence-Speed Coupling Enhances the Search Efficiency of Migrating Immune Cells. *Phys. Rev. Lett.* 125:268102.

36. Arcizet, D., S. Capito, M. Gorelashvili, C. Leonhardt, M. Vollmer, S. Youssef, S. Rappl, and D. Heinrich, 2012. Contact-controlled amoeboid motility induces dynamic cell trapping in 3D-microstructured surfaces. *Soft Matter* 8:1473–1481.
